# Supplementary material for: Cost-effectiveness of providing university students with a mindfulness-based intervention to reduce psychological distress: economic evaluation of a pragmatic randomised controlled trial
Source: BMJ Open. 2023 Nov 23;13(11):e071724. doi: 10.1136/bmjopen-2023-071724 (PMC10668272; doi:10.1136/bmjopen-2023-071724)
Supplement: Supplementary data [file bmjopen-2023-071724supp001.pdf]

Cost-effectiveness of providing university students with a mindfulness-based intervention to reduce psychological distress: economic evaluation of a pragmatic randomised controlled trial

SUPPLEMENTARY FILE

Adam P Wagner, Julieta Galante, Géraldine Dufour, Garry Barton, Jan Stochl, Maris Vainre, and Peter B Jones

12/09/2023

Adam P Wagner, PhD, (Corresponding author) Senior Research Fellow, Norwich Medical School, University of East Anglia, Norwich Research Park, Norwich, NR4 7TJ, United Kingdom (second affiliation: National Institute for Health Research (NIHR) Applied Research Collaboration (ARC) East of England (EoE)). adam.wagner@uea.ac.uk

Julieta Galante, PhD, Honorary Senior Visiting Research Fellow, Department of Psychiatry, University of Cambridge, Douglas House, 18b Trumpington Road, Cambridge, CB2 8AH, United Kingdom (second affiliation: Contemplative Studies Centre, Melbourne School of Psychological Sciences, Faculty of Medicine, Dentistry, and Health Sciences, University of Melbourne). mjg231@cam.ac.uk phone (+44) 7986 172834

Géraldine Dufour, MA, Geraldine Dufour Therapeutic Consultations Ltd https://www.geraldinedufour.com/ (second affiliation: Associate Executive Member, European Association for International Education (EAIE)).

Garry Barton, PhD, Professor of Health Economics, Norwich Medical School, University of East Anglia, Norwich Research Park, Norwich, NR4 7TJ, United Kingdom.

Jan Stochl, PhD, Senior Research Associate, Department of Psychiatry, University of Cambridge, Cambridge Biomedical Campus, Box 189, Cambridge, CB2 0QQ, United Kingdom (second affiliation: Department of Kinanthropology, Charles University, Jose Martiho 31, 162 52 Prague, Czech Republic; third affiliation: NIHR ARC EoE).

Maris Vainre, MA, PhD Student, MRC Cognition and Brain Sciences Unit, University of Cambridge, 15 Chaucer Rd, Cambridge, CB2 7EF, United Kingdom (former affiliation when doing this work: NIHR ARC EoE). Maris.Vainre@mrc-cbu.cam.ac.uk

Peter B Jones, PhD, Professor of Psychiatry, Department of Psychiatry, University of Cambridge, Herchel Smith Building, Cambridge, CB2 0SZ, United Kingdom (second affiliation: NIHR ARC EoE).

Wagner AP, *et al. BMJ Open* 2023; 13:e071724. doi: 10.1136/bmjopen-2023-071724

6 Supplementary materials

6.1 Missingness by scale and time point

Table 6.1: Missingness (percentage) by outcomes at each time, for each arm and overall.

	Percentage missing		
Outcome	MSS+SAU	SAU	Overall
CORE-6D, T0	0	1	0
CORE-6D, T1	18	27	22
CORE-6D, T2	24	30	27
CORE-6D, T3	45	45	45
CORE-OM, T0	3	4	3
CORE-OM, T1	19	28	23
CORE-OM, T2	26	30	28
CORE-OM, T3	46	46	46
WEMWBS, T0	1	0	0
WEMWBS, T1	18	28	23
WEMWBS, T2	24	30	27
WEMWBS, T3	46	46	46

Wagner AP, *et al. BMJ Open* 2023; 13:e071724. doi: 10.1136/bmjopen-2023-071724

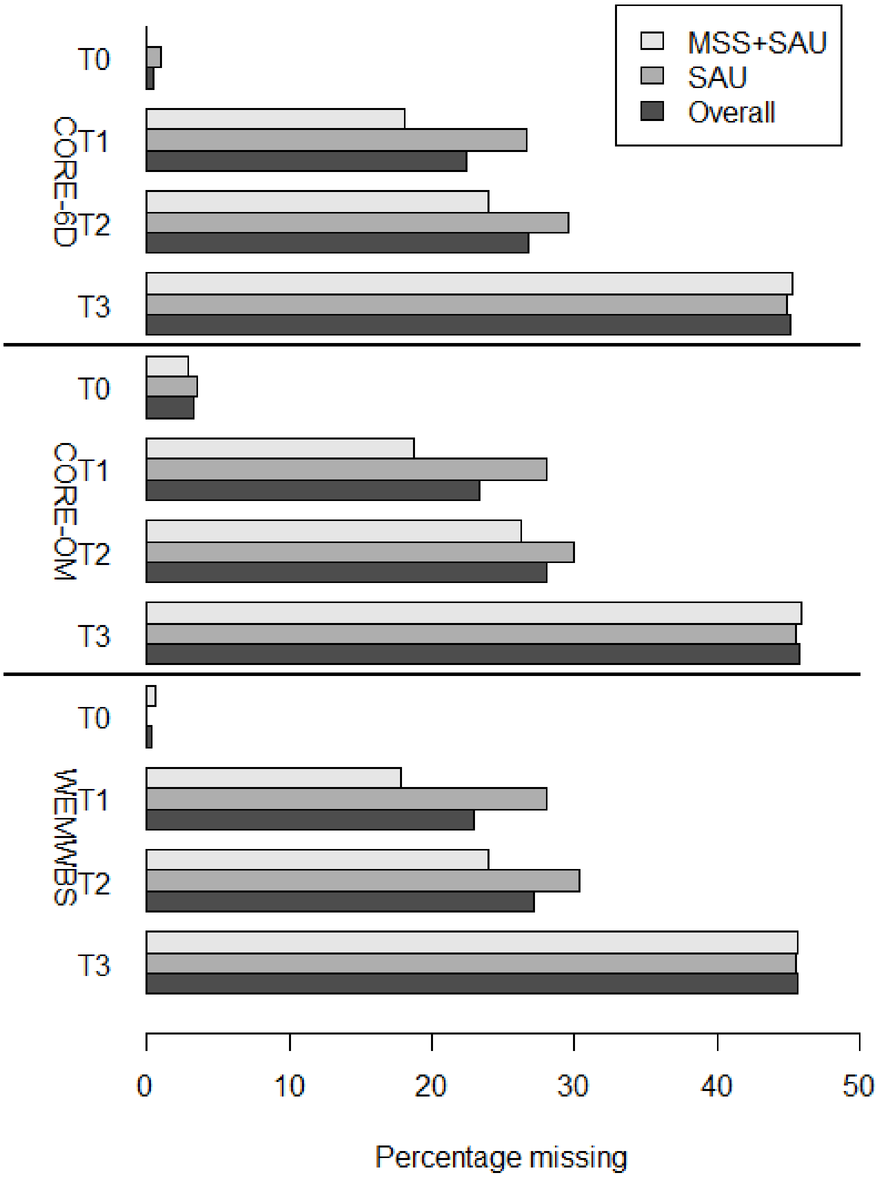

Figure 6.1: Missingness (percentage) by outcomes at each time, for each arm and overall.

6.2 Patterns of missingness and imputation model

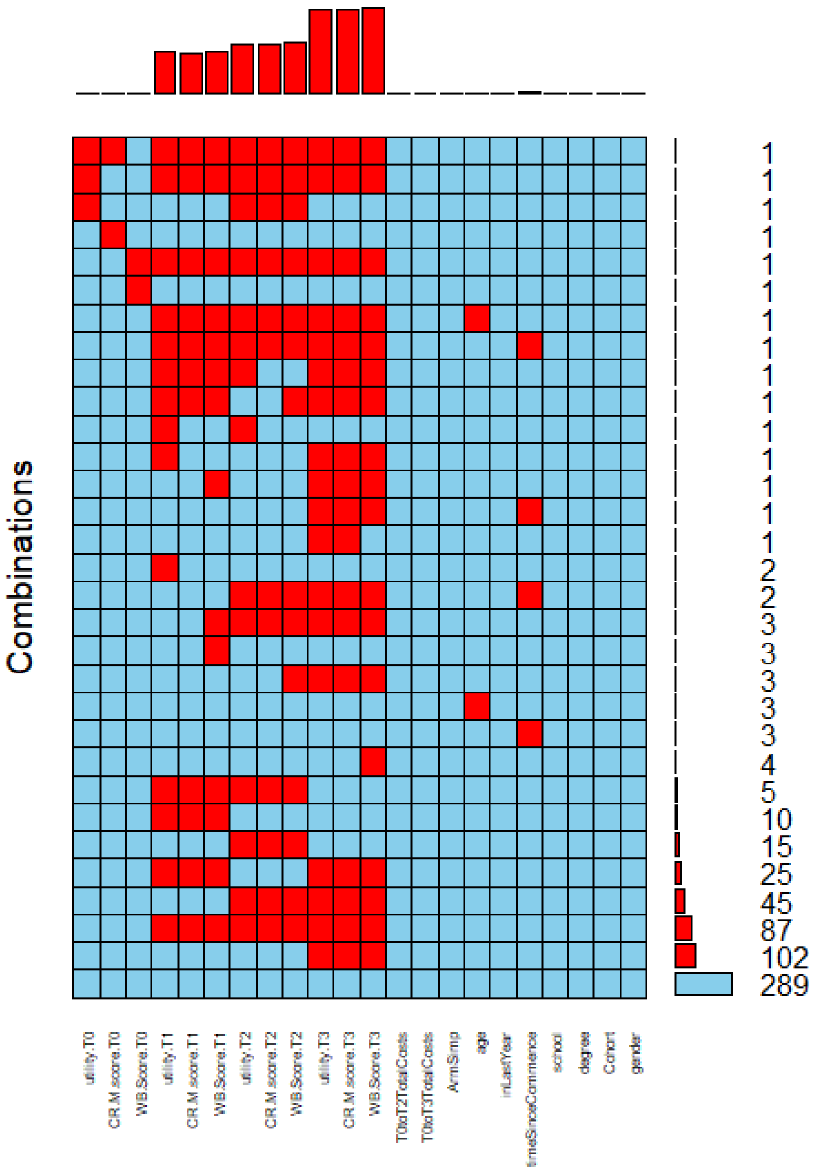

Figure 6.2: Patterns of missingness (red indicates missing) and variables included in imputation model.

6.3 Trial CONSORT diagram

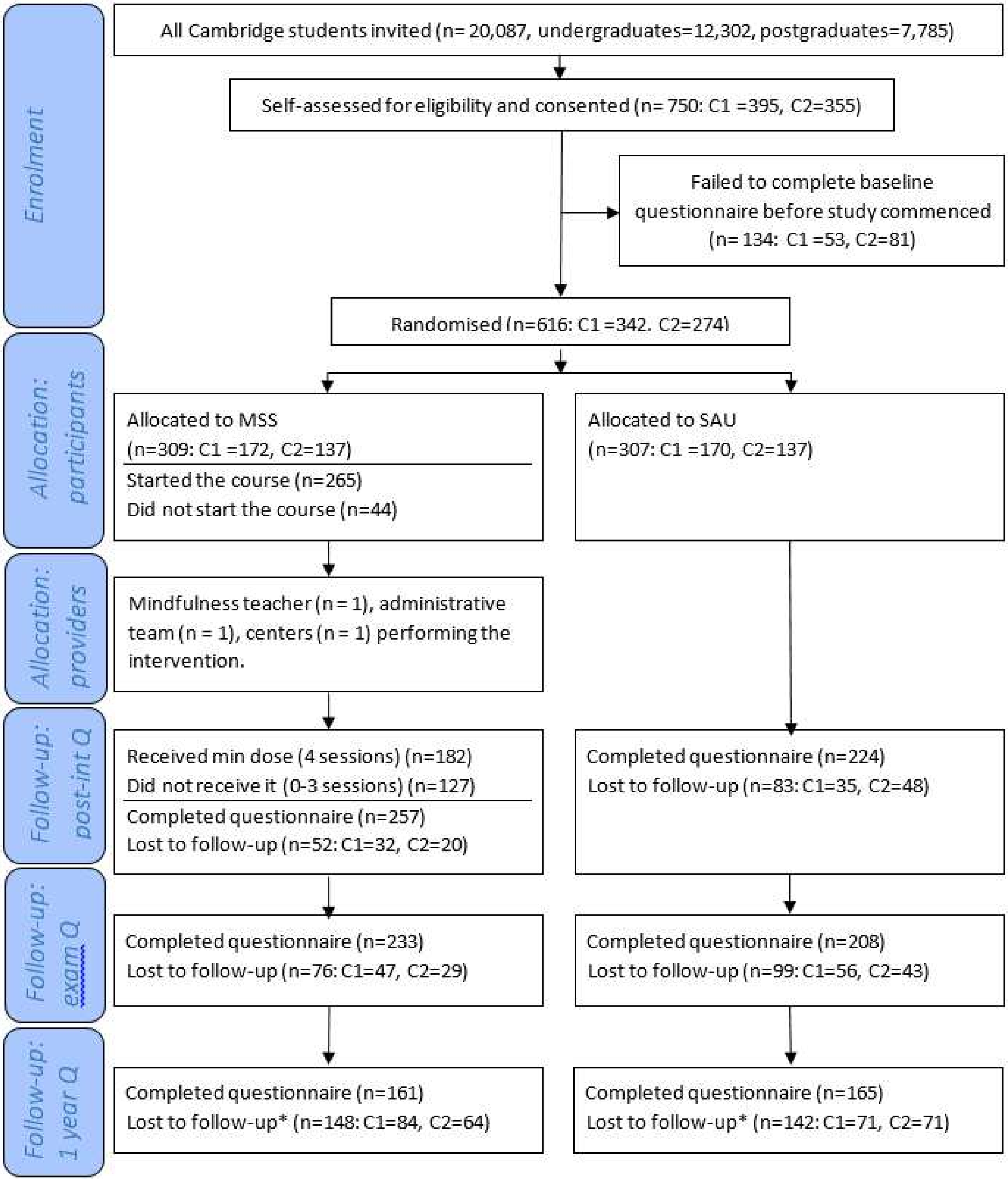

Figure 6.3: CONSORT 2010 diagram taken from Galante et al. (2021)

Wagner AP, *et al. BMJ Open* 2023; 13:e071724. doi: 10.1136/bmjopen-2023-071724

6.4 Resource use T0 to T2: Further detail

Table 6.2: Resource use across all participants, T0 to T2 (mean use per student)

	MSS+SAU (n=309)		SAU (n=307)		
Resource use T0 to T2	n/Mean	SD	n/Mean	SD	P-value
Assessment	0.09	0.30	0.11	0.33	0.5937
Counselling	0.30	1.31	0.47	1.70	0.1875
Client Contact by Phone or Email	0.00	0.00	0.00	0.00	-
MHA Assessment	0.01	0.08	0.01	0.08	0.9948
MHA Ongoing	0.00	0.06	0.03	0.36	0.2741
Pre Group	0.02	0.13	0.02	0.13	0.9918
Can't Work Group	0.01	0.08	0.00	0.00	0.1576
Returners - Anxiety	0.00	0.00	0.00	0.00	-
Returners - Self Compassion	0.00	0.00	0.00	0.00	-
Returners - Time Management	0.00	0.00	0.00	0.00	-
Workshop - Anxiety	0.01	0.10	0.01	0.08	0.6591
Workshop - CBT for self-help	0.00	0.06	0.00	0.06	0.9963
Workshop - Exam Preparation	0.00	0.06	0.00	0.06	0.9963
Workshop - Food and mood	0.01	0.11	0.00	0.00	0.0453
Workshop - Procrastination	0.01	0.11	0.02	0.13	0.7302
Workshop - Self-compassion	0.01	0.11	0.01	0.14	0.9934
Workshop - Sleep	0.00	0.00	0.00	0.00	-
Workshop - Social anxiety	0.00	0.00	0.00	0.00	-
Workshop - Panic attacks	0.01	0.08	0.00	0.00	0.1576
Assertiveness Group	0.01	0.08	0.00	0.06	0.5669
Bereavement Group	0.00	0.06	0.01	0.08	0.5601
M Phil Group	0.00	0.00	0.00	0.00	-
Managing Mood Group	0.00	0.06	0.00	0.00	0.3181
Perfectionism Group	0.00	0.00	0.00	0.06	0.3181
Post Graduate Group	0.00	0.00	0.00	0.00	-
Returners Group	0.01	0.08	0.00	0.06	0.5669
Self-esteem Group	0.00	0.06	0.00	0.06	0.9963
UG Group	0.00	0.06	0.00	0.00	0.3181

Wagner AP, *et al. BMJ Open* 2023; 13:e071724. doi: 10.1136/bmjopen-2023-071724

*Table 6.3: Resource use across participants *using* a particular service T0 to T2 (eg means here exclude participants not using that UCS offering)*

	MSS+SAU (n=309)		SAU (n=307)	
	Used (N)	Used mean	Used (N)	Used mean
Assessment	28	1.04	31	1.06
Counselling	27	3.48	37	3.86
Client Contact by Phone or Email	0	-	0	-
MHA Assessment	2	1.00	2	1.00
MHA Ongoing	1	1.00	2	4.00
Pre Group	5	1.00	5	1.00
Can't Work Group	2	1.00	0	-
Returners - Anxiety	0	-	0	-
Returners - Self Compassion	0	-	0	-
Returners - Time Management	0	-	0	-
Workshop - Anxiety	3	1.00	2	1.00
Workshop - CBT for self-help	1	1.00	1	1.00
Workshop - Exam Preparation	1	1.00	1	1.00
Workshop - Food and mood	4	1.00	0	-
Workshop - Procrastination	4	1.00	5	1.00
Workshop - Self-compassion	4	1.00	3	1.33
Workshop - Sleep	0	-	0	-
Workshop - Social anxiety	0	-	0	-
Workshop - Panic attacks	2	1.00	0	-
Assertiveness Group	2	1.00	1	1.00
Bereavement Group	1	1.00	2	1.00
M Phil Group	0	-	0	-
Managing Mood Group	1	1.00	0	-
Perfectionism Group	0	-	1	1.00
Post Graduate Group	0	-	0	-
Returners Group	2	1.00	1	1.00
Self-esteem Group	1	1.00	1	1.00
UG Group	1	1.00	0	-

Wagner AP, *et al. BMJ Open* 2023; 13:e071724. doi: 10.1136/bmjopen-2023-071724

6.5 Detailed unit costs

Table 6.4: Details of wage costs

Grade	Spine point	Salary (£)	Pension (£)	NI (£)	Apprent levy (£)	Total (£)	Rate (£/hr)
4	32	25,627	5,535	2,487	128	33,777	17.71
7	44	36,382	7,859	4,106	181	48,528	25.45

Wagner AP, *et al. BMJ Open* 2023; 13:e071724. doi: 10.1136/bmjopen-2023-071724

Table 6.5: Unit costs for each of the UCS offerings.

Type	Activity	Duration (mins)	Student contact (mins)	Occurrenc es	Group size	Total cost (£)	Unit cost (£)
Individual	Assessment	100	60	1	1	42.42	42.42
	Counselling	65	60	1	1	27.57	27.57
	Client Contact by Phone or Email	25	20	1	1	10.60	10.60
	MHA Assessment	100	60	1	1	42.42	42.42
	MHA Ongoing	65	60	1	1	27.57	27.57
	Pre Group	35	30	1	1	14.85	14.85
Workshop	Can't Work Group	135	120	1	6	57.26	9.54
	Returners - Anxiety	135	90	1	20	57.26	2.86
	Returners - Self Compassion	135	120	1	20	57.26	2.86
	Returners - Time Management	135	120	1	20	57.26	2.86
	Workshop - Anxiety	135	120	1	15	57.26	3.82
	Workshop - CBT for self-help	135	120	1	15	57.26	3.82
	Workshop - Exam Preparation	135	120	1	15	57.26	3.82
	Workshop - Food and mood	135	120	1	15	57.26	3.82
	Workshop - Procrastination	135	90	1	15	57.26	3.82
	Workshop - Self-compassion	135	120	1	15	57.26	3.82
	Workshop - Sleep	135	90	1	15	57.26	3.82
	Workshop - Social anxiety	135	120	1	15	57.26	3.82
	Workshop - Panic attacks	135	120	1	15	57.26	3.82
Group	Assertiveness Group	135	120	4	8	229.05	28.63
	Bereavement Group	135	120	6	8	343.57	42.95
	M Phil Group	135	120	5	8	286.31	35.79
	Managing Mood Group	135	120	6	8	343.57	42.95
	Perfectionism Group	135	120	5	8	286.31	35.79
	Post Graduate Group	135	120	14	8	801.67	100.21
	Returners Group	135	120	1	20	57.26	2.86
	Self-esteem Group	135	90	5	8	286.31	35.79
	UG Group	135	120	10	8	572.62	71.58

Wagner AP, *et al. BMJ Open* 2023; 13:e071724. doi: 10.1136/bmjopen-2023-071724

6.6 Costs T0 to T2: Further detail

Table 6.6: Mean costs per student across all participants, T0 to T2

	MSS+SAU (n=309)		SAU (n=307)		
UCS activity costs T0 to T2	n/Mean (£)	SD (£)	n/Mean (£)	SD (£)	P-value
Assessment	3.98	12.85	4.56	14.02	0.5937
Counselling	8.39	36.15	12.84	46.92	0.1875
Client Contact by Phone or Email	0.00	0.00	0.00	0.00	-
MHA Assessment	0.27	3.41	0.28	3.42	0.9948
MHA Ongoing	0.09	1.57	0.72	9.94	0.2741
Pre Group	0.24	1.88	0.24	1.88	0.9918
Can't Work Group	0.06	0.77	0.00	0.00	0.1576
Returners - Anxiety	0.00	0.00	0.00	0.00	-
Returners - Self Compassion	0.00	0.00	0.00	0.00	-
Returners - Time Management	0.00	0.00	0.00	0.00	-
Workshop - Anxiety	0.04	0.37	0.02	0.31	0.6591
Workshop - CBT for self-help	0.01	0.22	0.01	0.22	0.9963
Workshop - Exam Preparation	0.01	0.22	0.01	0.22	0.9963
Workshop - Food and mood	0.05	0.43	0.00	0.00	0.0453
Workshop - Procrastination	0.05	0.43	0.06	0.48	0.7302
Workshop - Self-compassion	0.05	0.43	0.05	0.53	0.9934
Workshop - Sleep	0.00	0.00	0.00	0.00	-
Workshop - Social anxiety	0.00	0.00	0.00	0.00	-
Workshop - Panic attacks	0.02	0.31	0.00	0.00	0.1576
Assertiveness Group	0.19	2.30	0.09	1.63	0.5669
Bereavement Group	0.14	2.44	0.28	3.46	0.5601
M Phil Group	0.00	0.00	0.00	0.00	-
Managing Mood Group	0.14	2.44	0.00	0.00	0.3181
Perfectionism Group	0.00	0.00	0.12	2.04	0.3181
Post Graduate Group	0.00	0.00	0.00	0.00	-
Returners Group	0.02	0.23	0.01	0.16	0.5669
Self-esteem Group	0.12	2.04	0.12	2.04	0.9963
UG Group	0.23	4.07	0.00	0.00	0.3181

6.7 Resource use T0 to T3

Table 6.7: Aggregated UCS resource use by arm between T0 to T2. Means are the amount of each activity by student. SD= standard deviation

	MSS+SAU (n=309)		SAU (n=307)		
Resource use T0 to T3	n/Mean	SD	n/Mean	SD	P-value
Used UCS services: N=	64		73		
Individual assessment sessions	0.15	0.39	0.17	0.40	0.6607
Individual counselling sessions	0.52	1.76	0.72	2.68	0.2773
Total other individual activity	0.04	0.20	0.08	0.54	0.1375
Total workshops	0.07	0.34	0.05	0.26	0.3611
Total groups	0.03	0.18	0.03	0.16	0.6428

Wagner AP, *et al. BMJ Open* 2023; 13:e071724. doi: 10.1136/bmjopen-2023-071724

Table 6.8: Resource use across all participants, T0 to T3 (mean use per student)

	MSS+SAU (n=309)		SAU (n=307)		
Resource T0 to T3	n/Mean	SD	n/Mean	SD	P-value
Assessment	0.15	0.39	0.17	0.40	0.6607
Counselling	0.52	1.76	0.72	2.68	0.2773
Client Contact by Phone or Email	0.00	0.00	0.00	0.06	0.3181
MHA Assessment	0.01	0.10	0.02	0.14	0.3104
MHA Ongoing	0.00	0.06	0.04	0.47	0.1454
Pre Group	0.02	0.15	0.02	0.14	0.7887
Can't Work Group	0.01	0.11	0.01	0.08	0.4169
Returners - Anxiety	0.00	0.00	0.00	0.00	-
Returners - Self Compassion	0.00	0.00	0.00	0.00	-
Returners - Time Management	0.00	0.00	0.00	0.00	-
Workshop - Anxiety	0.01	0.10	0.01	0.08	0.6591
Workshop - CBT for self-help	0.00	0.06	0.00	0.06	0.9963
Workshop - Exam Preparation	0.00	0.06	0.00	0.06	0.9963
Workshop - Food and mood	0.01	0.11	0.00	0.00	0.0453
Workshop - Procrastination	0.01	0.11	0.02	0.13	0.7302
Workshop - Self-compassion	0.01	0.11	0.01	0.14	0.9934
Workshop - Sleep	0.00	0.00	0.00	0.00	-
Workshop - Social anxiety	0.00	0.00	0.00	0.00	-
Workshop - Panic attacks	0.01	0.08	0.00	0.06	0.5669
Assertiveness Group	0.01	0.10	0.01	0.08	0.6591
Bereavement Group	0.00	0.06	0.01	0.08	0.5601
M Phil Group	0.00	0.00	0.00	0.00	-
Managing Mood Group	0.00	0.06	0.00	0.00	0.3181
Perfectionism Group	0.00	0.00	0.00	0.06	0.3181
Post Graduate Group	0.00	0.06	0.00	0.06	0.9963
Returners Group	0.01	0.08	0.00	0.06	0.5669
Self-esteem Group	0.00	0.06	0.00	0.06	0.9963
UG Group	0.00	0.06	0.00	0.00	0.3181

*Table 6.9: Resource use across participants *using* a particular service T0 to T3 (eg means here exclude participants not using that UCS offering)*

	MSS+SAU (n=309)		SAU (n=307)	
	Used (N)	Used mean	Used (N)	Used mean
Assessment	43	1.09	48	1.06
Counselling	41	3.93	49	4.51
Client Contact by Phone or Email	0	-	1	1.00
MHA Assessment	3	1.00	6	1.00
MHA Ongoing	1	1.00	3	4.33
Pre Group	7	1.00	6	1.00
Can't Work Group	4	1.00	2	1.00
Returners - Anxiety	0	-	0	-
Returners - Self Compassion	0	-	0	-
Returners - Time Management	0	-	0	-
Workshop - Anxiety	3	1.00	2	1.00
Workshop - CBT for self-help	1	1.00	1	1.00
Workshop - Exam Preparation	1	1.00	1	1.00
Workshop - Food and mood	4	1.00	0	-
Workshop - Procrastination	4	1.00	5	1.00
Workshop - Self-compassion	4	1.00	3	1.33
Workshop - Sleep	0	-	0	-
Workshop - Social anxiety	0	-	0	-
Workshop - Panic attacks	2	1.00	1	1.00
Assertiveness Group	3	1.00	2	1.00
Bereavement Group	1	1.00	2	1.00
M Phil Group	0	-	0	-
Managing Mood Group	1	1.00	0	-
Perfectionism Group	0	-	1	1.00
Post Graduate Group	1	1.00	1	1.00
Returners Group	2	1.00	1	1.00
Self-esteem Group	1	1.00	1	1.00
UG Group	1	1.00	0	-

6.8 Costs T0 to T3

Table 6.10: Mean cost (per student) incurred by the UCS, T0 to T3

	MSS+SAU (n=309)		SAU (n=307)		
Costs T0 to T3	n/Mean (£)	SD (£)	n/Mean (£)	SD (£)	P-value
Individual assessment sessions	6.45	16.72	7.05	16.89	0.6607
Individual counselling sessions	14.37	48.65	19.85	73.82	0.2773
Total other individual activity	0.84	5.65	2.32	15.71	0.1201
Total workshops	0.36	1.66	0.24	1.22	0.2991
Total groups	1.25	8.48	1.04	7.59	0.7448
MSS course	52.82	0.00	0.00	0.00	
Total	76.07	63.27	30.49	84.97	0.0000

Table 6.11: Mean costs per student across all participants, T0 to T3

	MSS+SAU (n=309)		SAU (n=307)		
UCS activity costs T0 to T3	n/Mean (£)	SD (£)	n/Mean (£)	SD (£)	P-value
Assessment	6.45	16.72	7.05	16.89	0.6607
Counselling	14.37	48.65	19.85	73.82	0.2773
Client Contact by Phone or Email	0.00	0.00	0.03	0.61	0.3181
MHA Assessment	0.41	4.17	0.83	5.88	0.3104
MHA Ongoing	0.09	1.57	1.17	12.85	0.1454
Pre Group	0.34	2.21	0.29	2.06	0.7887
Can't Work Group	0.12	1.08	0.06	0.77	0.4169
Returners - Anxiety	0.00	0.00	0.00	0.00	-
Returners - Self Compassion	0.00	0.00	0.00	0.00	-
Returners - Time Management	0.00	0.00	0.00	0.00	-
Workshop - Anxiety	0.04	0.37	0.02	0.31	0.6591
Workshop - CBT for self-help	0.01	0.22	0.01	0.22	0.9963
Workshop - Exam Preparation	0.01	0.22	0.01	0.22	0.9963
Workshop - Food and mood	0.05	0.43	0.00	0.00	0.0453
Workshop - Procrastination	0.05	0.43	0.06	0.48	0.7302
Workshop - Self-compassion	0.05	0.43	0.05	0.53	0.9934
Workshop - Sleep	0.00	0.00	0.00	0.00	-
Workshop - Social anxiety	0.00	0.00	0.00	0.00	-
Workshop - Panic attacks	0.02	0.31	0.01	0.22	0.5669
Assertiveness Group	0.28	2.81	0.19	2.31	0.6591
Bereavement Group	0.14	2.44	0.28	3.46	0.5601
M Phil Group	0.00	0.00	0.00	0.00	-
Managing Mood Group	0.14	2.44	0.00	0.00	0.3181
Perfectionism Group	0.00	0.00	0.12	2.04	0.3181
Post Graduate Group	0.32	5.70	0.33	5.72	0.9963
Returners Group	0.02	0.23	0.01	0.16	0.5669
Self-esteem Group	0.12	2.04	0.12	2.04	0.9963
UG Group	0.23	4.07	0.00	0.00	0.3181

6.9 CORE-6D utilities by arm and cohort

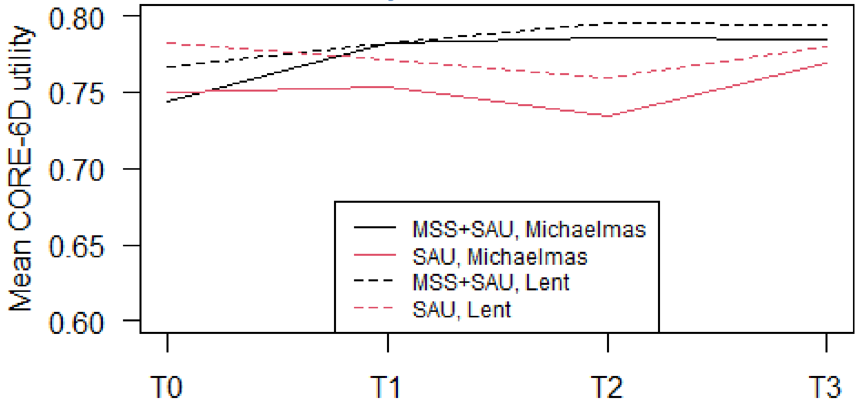

Figure 6.4: Mean (per student) CORE-6D utilities by arm and cohort (N within each group varies by time point – see Table 1).

6.10 Cost-effectiveness planes

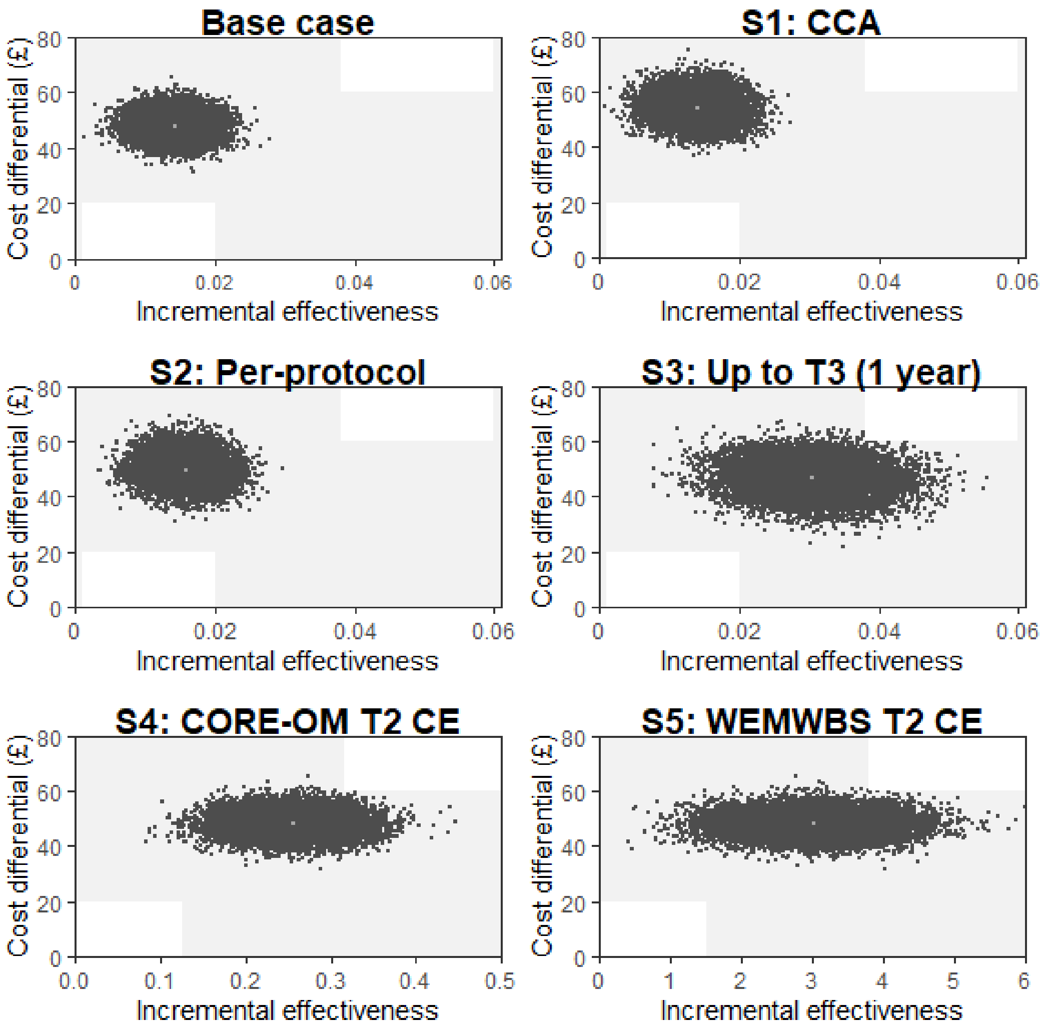

Figure 6.5: Cost-effectiveness planes resulting from the bootstrap re-sampling

Wagner AP, *et al. BMJ Open* 2023; 13:e071724. doi: 10.1136/bmjopen-2023-071724
