# Supplementary material for: Cost-effectiveness of providing university students with a mindfulness-based intervention to reduce psychological distress: economic evaluation of a pragmatic randomised controlled trial
Source: BMJ Open. 2023 Nov 23;13(11):e071724. doi: 10.1136/bmjopen-2023-071724 (PMC10668272; doi:10.1136/bmjopen-2023-071724)
Supplement: Author's manuscript [file bmjopen-2023-071724.draft_revisions.pdf]

BMJ Open is committed to open peer review. As part of this commitment we make the peer review history of every article we publish publicly available.

When an article is published we post the peer reviewers' comments and the authors' responses online. We also post the versions of the paper that were used during peer review. These are the versions that the peer review comments apply to.

The versions of the paper that follow are the versions that were submitted during the peer review process. They are not the versions of record or the final published versions. They should not be cited or distributed as the published version of this manuscript.

BMJ Open is an open access journal and the full, final, typeset and author-corrected version of record of the manuscript is available on our site with no access controls, subscription charges or pay-per-view fees (http://bmjopen.bmj.com).

If you have any questions on BMJ Open's open peer review process please email info.bmjopen@bmj.com

BMJ Open

Cost-effectiveness of providing university students with a mindfulness-based intervention to increase resilience to stress: economic evaluation of a pragmatic randomised controlled trial

Journal:	*BMJ Open*
Manuscript ID	bmjopen-2023-071724
Article Type:	Original research
Date Submitted by the Author:	19-Jan-2023

Complete List of Authors:	Wagner, Adam; NIHR Applied Research Collaboration (ARC) East of England (EoE), ; University of East Anglia, Norwich Medical School<br>Galante, Julieta; University of Cambridge, Department of Psychiatry; University of Melbourne, Contemplative Studies Centre, Melbourne School of Psychological Sciences, Faculty of Medicine, Dentistry, and Health Sciences<br>Dufour, Géraldine; Therapeutic Consultations Ltd ; European Association for International Education<br>Barton, Garry; University of East Anglia, Norwich Medical School<br>Stochl, Jan; University of Cambridge, Department of Psychiatry; Charles University, Department of Kinanthropology<br>Vainre, Maris; Cambridge University, MRC Cognition and Brain Sciences Unit<br>Jones, Peter; University of Cambridge, Department of Psychiatry; NIHR Applied Research Collaboration (ARC) East of England (EoE)

Keywords:	HEALTH ECONOMICS, MENTAL HEALTH, PSYCHIATRY

SCHOLARONE™
Manuscripts

I, the Submitting Author has the right to grant and does grant on behalf of all authors of the Work (as defined in the below author licence), an exclusive licence and/or a non-exclusive licence for contributions from authors who are: i) UK Crown employees; ii) where BMJ has agreed a CC-BY licence shall apply, and/or iii) in accordance with the terms applicable for US Federal Government officers or employees acting as part of their official duties; on a worldwide, perpetual, irrevocable, royalty-free basis to BMJ Publishing Group Ltd ("BMJ") its licensees and where the relevant Journal is co-owned by BMJ to the co-owners of the Journal, to publish the Work in this journal and any other BMJ products and to exploit all rights, as set out in our licence.

The Submitting Author accepts and understands that any supply made under these terms is made by BMJ to the Submitting Author unless you are acting as an employee on behalf of your employer or a postgraduate student of an affiliated institution which is paying any applicable article publishing charge ("APC") for Open Access articles. Where the Submitting Author wishes to make the Work available on an Open Access basis (and intends to pay the relevant APC), the terms of reuse of such Open Access shall be governed by a Creative Commons licence – details of these licences and which Creative Commons licence will apply to this Work are set out in our licence referred to above.

Other than as permitted in any relevant BMJ Author's Self Archiving Policies, I confirm this Work has not been accepted for publication elsewhere, is not being considered for publication elsewhere and does not duplicate material already published. I confirm all authors consent to publication of this Work and authorise the granting of this licence.

For peer review only

Cost-effectiveness of providing university students with a mindfulness-based intervention to increase resilience to stress: economic evaluation of a pragmatic randomised controlled trial

Adam P Wagner, Julieta Galante, Géraldine Dufour, Garry Barton, Jan Stochl, Maris Vainre, and Peter B Jones

19/01/2023

Adam P Wagner, PhD, (Corresponding author) Senior Research Fellow, Norwich Medical School, University of East Anglia, Norwich Research Park, Norwich, NR4 7TJ, United Kingdom (second affiliation: National Institute for Health Research (NIHR) Applied Research Collaboration (ARC) East of England (EoE)). adam.wagner@uea.ac.uk

Julieta Galante, PhD, Honorary Senior Visiting Research Fellow, Department of Psychiatry, University of Cambridge, Douglas House, 18b Trumpington Road, Cambridge, CB2 8AH, United Kingdom (second affiliation: Contemplative Studies Centre, Melbourne School of Psychological Sciences, Faculty of Medicine, Dentistry, and Health Sciences, University of Melbourne). mjg231@cam.ac.uk phone (+44) 7986 172834

Géraldine Dufour, MA, Geraldine Dufour Therapeutic Consultations Ltd https://www.geraldinedufour.com/ (second affiliation: Associate Executive Member, European Association for International Education (EAIE)).

Garry Barton, PhD, Professor of Health Economics, Norwich Medical School, University of East Anglia, Norwich Research Park, Norwich, NR4 7TJ, United Kingdom.

Jan Stochl, PhD, Senior Research Associate, Department of Psychiatry, University of Cambridge, Cambridge Biomedical Campus, Box 189, Cambridge, CB2 0QQ, United Kingdom (second affiliation: Department of Kinanthropology, Charles University, Jose Martiho 31, 162 52 Prague, Czech Republic; third affiliation: NIHR ARC EoE).

Maris Vainre, MA, PhD Student, MRC Cognition and Brain Sciences Unit, University of Cambridge, 15 Chaucer Rd, Cambridge, CB2 7EF, United Kingdom (former affiliation when doing this work: NIHR ARC EoE). Maris.Vainre@mrc-cbu.cam.ac.uk

Peter B Jones, PhD, Professor of Psychiatry, Department of Psychiatry, University of Cambridge, Herchel Smith Building, Cambridge, CB2 0SZ, United Kingdom (second affiliation: NIHR ARC EoE).

Word count: 4,196

For peer review only

Abstract

Objective: Increasing numbers of young people attending university has raised concerns about the capacity of student mental health services to support them. We conducted a randomised controlled trial (RCT) to explore whether provision of an 8-week mindfulness course adapted for university students (Mindfulness Skills for Students – MSS), compared to university mental health support as usual (SAU), increased resilience to stress. Here, we conduct an economic evaluation of MSS+SAU compared to SAU.

Design and setting: Economic evaluation conducted alongside a pragmatic, parallel, single-blinded randomised controlled trial (RCT) comparing provision of MSS+SAU to SAU.

Participants: 616 university students randomised.

Primary and secondary outcome measures: The primary economic evaluation assessed the cost per quality-adjusted life year (QALY) gained from the perspective of the university counselling service. Costs relate to staff time required to deliver counselling service offerings. QALYs were derived from the CORE-6D preference based tool, which uses responses to six items of the Clinical Outcomes in Routine Evaluation Outcome Measure (CORE-OM; the study's primary clinical outcome measure).

Results: It was estimated to cost £1584.48 (2022 prices) to deliver an MSS course to 30 students, £52.82 per student. Both costs(adjusted mean difference: £48.04, 95%CI £39.92-£56.16) and QALYs (adjusted mean difference: 0.014, 95%CI 0.008-0.021) were significantly higher in the MSS arm compared to SAU. The incremental cost-effectiveness ratio (ICER) was £3,355, with a very high (99.99%) probability of being cost-effective at a willingness-to-pay threshold of £20,000 per QALY.

Conclusions: MSS leads to significantly improved outcomes at a moderate additional cost. The ICER of £3,355 per QALY suggests that MSS is cost-effective when compared to the UK's National Institute for Health and Care Excellence (NICE) thresholds of £20,000 per QALY.

Trial registration: Australian and New Zealand Clinical Trials Registry, ACTRN12615001160527

286/300 words

Strengths and limitations of this study

- Contrasting with many evaluations of mindfulness, a key strength of this study/economic evaluation is that it drew on data from a large ($n > 600$) RCT that followed-up participants for up to 12 months.
- A key limitation is the narrow costing perspective adopted – the university counselling service – that does not capture all costs of mental health support and will miss wider impacts.
- Additionally, we have focused exclusively on wage costs (including on-costs) – we have not considered wider overheads or other resources (such as room hire), subsequently underestimating costs.

For peer review only - http://bmjopen.bmj.com/site/about/guidelines.xhtml

1 Background

University counselling services in the UK have seen a rising trend in the proportion of students asking for mental health support [1]; indeed, it has been noted that the increase in student numbers seeking counselling is greater than the growth of overall student numbers [2]. Similar issues have been noted in the university sector beyond England [3,4]. Thus, an effective preventative intervention is needed to address this growing demand and need.

A widely used working definition of mindfulness is `the awareness that emerges through paying attention on purpose, in the present moment, and non-judgmentally to the unfolding of experience moment by moment' [5]. It is popular among students [6], and some of the popularity may be because it seeks to teach skills, rather than directly addressing mental health issues – it is non-stigmatising [7].

Given the growing demand for counselling services and limited university funding, it is important to have evidence for the effectiveness and cost implications of associated interventions [8]. Thus, when the University of Cambridge's (UoC's) University Counselling Service (UCS) developed the 'Mindfulness Skills for Students' (MSS) programme, they evaluated it in the Mindful Student Study: a large randomised controlled trial (RCT) [9]. It has been demonstrated that, compared to support as usual (SAU), MSS + SAU reduces students' psychological distress during the academic examination period, three to six months after randomisation [10]. MSS participants were followed up for 12 months post randomisation: compared to support as usual (SAU), MSS + SAU continued to have small but significant reductions in distress at this time [11]. Here, we conduct an economic evaluation of MSS to explore its costs.

2 Method

2.1 Participants

The Mindful Student Study was a pragmatic randomised trial at the University of Cambridge (UoC) in the United Kingdom (UK). Students aged 18 or older who self-assessed as having no severe mental illness or crisis underwent 1:1 randomisation to receive either: an eight week mindfulness course adapted for university students plus support as usual (MSS+SAU); or SAU alone. Further detail on the trial can be found in [9] and [10].

Students were recruited in two cohorts: at the beginning of the autumn term (Michaelmas) term and at the beginning of the spring (Lent) term. Both cohorts were followed-up at: point of recruitment (T0); after the delivery of the mindfulness courses (SAU having *not* received the course) (T1); during the exam term (T2); and 12 months after recruitment (T3). The date of exam term follow-up, T2, was common across cohorts, but the date of other follow-up points differed by cohort: the relationships are depicted in Figure 1.

This study was approved by the Cambridge Psychology Research Ethics committee on August 25, 2015 (reference: PRE.2015.060). The study is registered with the Australia and New Zealand Clinical Trials Registry (ACTRN12615001160527). The Health Economic

For peer review only - http://bmjopen.bmj.com/site/about/guidelines.xhtml

Analysis Plan (HEAP) that this economic evaluation follows was pre-specified and is available online [12].

2.2 Costs

Costs were estimated from the perspective of the UoC's operating budget (primarily covering salaries of staff; excludes room hire) - hereafter referred to as the `UCS perspective'. Use of SAU UCS offerings were extracted from the UCS record system (consequently, there is no missing data on UCS service use) following participant agreements and strict confidentiality of access protocols .

At the time of this evaluation, routine UCS services were categorised into three broad groups: 'individual' sessions providing support to one student (includes assessment sessions); 'workshops' - one-off offerings that more than one student attends; and 'groups' - a number of sessions attended by multiple students (where a student stops attending a group, their place cannot be used by another student). In consultation with the then UCS head (GD), who consulted the UCS senior management team, we determined the usual staff grade delivering each offering and the associated typical time taken to prepare, deliver and record (e.g. completing notes etc) each of these session types. To calculate the cost to the UCS of a given offering, we multiplied the time taken to prepare, deliver and record it by the hourly rate of employing staff at the required grade. For workshops and groups, the cost per student (unit cost) of an offering is determined by dividing its total cost by the number of students typically attending each offering (details given in Supplementary materials Section 6.5).

The total cost of delivering a course of mindfulness (the intervention) to 30 students (maximum capacity of a course) was determined through consultation with the then UCS head (GD) and the course teacher. Accordingly, the activities involved in delivering a course were identified, along with the total time, and grade of staff, delivering each activity. Total times were multiplied by appropriate hourly rates of staff delivering each course activity and summed to give the total cost of delivering a Mindfulness course. The cost per student (unit cost) of delivering the course was determined by dividing this total cost by the capacity of each class ($n = 30$ students). This cost was ascribed to each of the students in the MSS+SAU arm (multiple courses were being delivered in parallel for each cohort of MSS+SAU). This cost was irrespective of student attendance: the nature of the mindfulness course is such that a student cannot join part way through, so a non-attending student cannot be `replaced'; this is in line with other UCS groups.

Hourly cost of employing staff were taken from the UoC Human Resources website and include on-costs (such as pension and national insurance contribution – see Supplementary materials Section 6.5) but no further overheads. They were the rates as applied from March 2022, and so can be considered to apply to the 2022/2023 financial year. Costs are reported in pound sterling.

For peer review only - http://bmjopen.bmj.com/site/about/guidelines.xhtml

2.3 Outcomes

The primary outcome measure was the participant completed Clinical Outcomes in Routine Evaluation Outcome Measure (CORE-OM), a 34-item generic questionnaire designed to evaluate efficacy and effectiveness across multiple disciplines delivering psychological therapies [13]. The CORE-OM has been found to have strong convergent validity, internal and test-retest reliability and sensitivity to change [14], and has been extensively used with UK university students [15]. Each CORE-OM item is scored on a five-point scale ranging from 0 (not at all) to 4 (most or all the time). The corresponding sum-total score ranges 0–136; this is usually divided by the number of completed items to form a total mean score. A higher mean-score indicates more distress. CORE-OM was collected at each of the data collection points (T0-T3); a comparison of CORE-OM between arms during the exam term (T2) was the primary (clinical effectiveness) outcome [9,10].

The CORE-6D is a preference-based tool to derive mental health specific health state utility, based on a subset of six questions/dimensions from the CORE-OM [16]: using time trade-off methods, [17] a sample of the general public were surveyed for their preferences for different health states as described by the CORE-6D [18]. Scores generated from the survey are converted to an index score, that can be used as a valuation of all health states represented by the CORE-6D. Here, utility scores were calculated for T0-T3 using this valuation. In line with the primary clinical-effectiveness outcome, the base-case (primary) economic analysis uses quality adjusted life years (QALYs) from T0 to T2 as outcome; these are calculated via the area under the curve approach [19] with linear interpolation between points. Given different periods between cohorts in which to accrue QALYs (see Figure 1: Michaelmas cohort – 227 days; Lent – 147 days), QALYs are reported separately for each cohort (and subsequent regressions adjust for cohort). Additionally, we also calculated QALYs from T0 to T3.

The Warwick-Edinburgh Mental Wellbeing Scale (WEMWBS) was used at each time point to measure a broad conception of subjective well-being [20]. The WEMWBS has 14 items, each scored from one ('none of the time') to five ('all of the time'). A higher total score (ranging from 14-70) indicates higher wellbeing. The WEMWBS has good validity, internal consistency and test-retest reliability in samples of both UK students and general population [21].

2.4 Analyses

To reduce the risk of bias [22] and improve precision [23], we sought to address missing data, rather than conducting a complete case analysis (CCA). Following recommendations for within trial analyses of cost-effectiveness, patterns of missing data were explored to understand the potential missing data mechanisms [23]. Resource use information was complete as it came from internal UCS records. Where missingness occurred on the outcome scales, it nearly always meant that *no* items had been completed (see Supplementary materials Section 7.1). Thus, we focused on summary measures of the outcomes (e.g. utilities, CORE-OM mean-score and WEMWBS total) in further missing data considerations. Details of missingness by outcome at each time point, overall and by arm, are given in Section 6.1: missingness increased by time point, and was between 5-10%

For peer review only - http://bmjopen.bmj.com/site/about/guidelines.xhtml

higher in the SAU at T1 and T2, but were similar at T3. As advised in [23], we explored associations between missingness and both i) baseline variables (see Section 7.2) and observed outcomes (see Section 7.2.1). The associations indicated that the data was not missing completely at random (MCAR) – meaning that CCA would not be appropriate. In line with the analysis devised in [9] and conducted in [10], we assumed the data was missing at random (MAR), and used multiple-imputation (MI) to create fifty datasets (following the rule-of-thumb from [24] that the number of data-sets should equal the percentage of missing data). Note that the level of missing data at T3 ($\approx 45\%$) is notably higher than at T2 ($\approx 27\%$), with the latter time point being the most crucial for the base case which draws data from T0-T2 (the impact of imputing missing data is explored in the sensitivity analyses - see below).

The R package *mice* [25] was used to conduct MI, separately for each arm (as advised in [23]) using the significant ($p < 0.1$) predictors of missingness and those variables included in the final regression models (as advised in [24]): see Supplementary materials Section 6.2 for details. The separate data-sets were combined using Rubin's rules [26].

Costs and outcomes were analysed simultaneously with bivariate regression models, allowing for correlations between costs and outcomes to be incorporated (in contrast to separate regressions). Such regressions are generally robust to skewed data [27]. Data was analysed using an intention-to-treat (ITT) approach: participants were analysed in the group to which they were randomised. All regressions (both cost and outcome) included covariates for: arm; sex; and cohort. Outcome regressions additionally included the baseline measure of the corresponding outcome (or utility, when analysing QALYs).

The estimated coefficient of arm in these regressions allowed the incremental cost-effectiveness ratio (ICER) to be estimated [28]: in this context, this corresponded to the mean incremental cost divided by the mean incremental effect (for the QALYs, COME-OM and WEMWBS outcomes) for MSS + SAU compared to SAU. However, were either alternative – MSS + SAU or SAU – both less costly and more effective, that alternative would be categorised as 'dominant' and the ICER would not be calculated. Otherwise, the ICER can be used to measure whether the extra cost associated with MSS + SAU is considered to constitute value for money. In other contexts, such as economic evaluations of new interventions for use in the United Kingdom's (UK's) National Health Service (NHS), the National Institute for Health and Care Excellence (NICE; [29]) uses a cost-effectiveness threshold value (λ) of £20,000-30,000 per QALY [30]. There is no agreed threshold for the perspective of costs to the UCS.

Uncertainty in the mean incremental costs and mean incremental effect (for each outcome), which determines estimated levels of cost-effectiveness, were explored using bootstrap re-sampling [31]: 200 replications were taken from each of the 50 imputations (in line with [23]), with re-sampling stratified by sex and cohort. From the replicates, cost-effectiveness planes were constructed for the base case and each scenario; each plane (one per outcome) displays the estimated mean incremental cost and mean incremental effect for each replicate [32], with location and spread of points showing uncertainty. Additionally, cost-effectiveness acceptability curves (CEACs) were constructed for the base case and each

For peer review only - http://bmjopen.bmj.com/site/about/guidelines.xhtml

scenario, and show the probability of MSS + SAU being cost-effective compared to SAU at a range of willingness-to-pay thresholds [33].

In the base-case (primary) economic analysis, the QALYs and costs up to T2 – in line with the primary clinical-effectiveness outcome in [10] – are considered. To check the robustness of the corresponding conclusions, a number of sensitivity analyses [28] were conducted in which the assumptions of the base-case analysis were varied:

1. CCA – repeats the base case, but only including participants for whom we have complete data; investigates the impact of MI;

2. Per-protocol analysis – investigates how results differ if we only consider those attending at least 50% (four out of eight) of the mindfulness sessions;

3. Conduct evaluation at 12 months – extend the base-case analysis to include costs and benefits up to T3, investigating whether benefits continue beyond the examination term;

4. Conduct a cost-effectiveness analysis (CEA) for CORE-OM – as for the base-case, but measure benefits in terms of CORE-OM at T2 (e.g., calculate the cost per CORE-OM point);

5. Conduct a CEA for WEMWBS – as for the base-case, but measure benefits in terms of WEMWBS at T2 (e.g., calculate the cost per WEMWBS point).

All analysis were conducted in *R* [34]. No costs or benefits were considered beyond 12 months, so no discounting was undertaken [28,30].

2.5 Patient and Public Involvement statement

Study plans were reviewed by a group comprising representatives from the UCS, the university Academic Division, student representatives and college tutors. A focus group with students who had completed mindfulness courses taught before the trial was conducted to consult about study plans prior to submission for ethics approval.

Subsequently, an advisory reference group was put together comprising student representatives, members of the UCS and other student welfare staff. This group met approximately three times per year during the trial. Study researchers attended these meetings and presented updates. The group also advised on appropriate dissemination strategies.

For peer review only - http://bmjopen.bmj.com/site/about/guidelines.xhtml

3 Results

3.1 Participants

A total of 616 students were recruited to the trial during the academic year 2016/2017 (see Figure 1): 342 were recruited during September 2016 (Michaelmas cohort), 172 to MSS + SAU and 170 to SAU; 274 were during January 2017 (Lent cohort), 137 students to each arm. A CONSORT diagram of participants is given in Section 6.3. Both arms (see Table 1) had an approximate equal age of 23 years, but MSS + SAU had proportionately fewer females: 61% versus 65%, a non-significant difference. Other variables (including: ethnic origin; disability; degree level; year of study and department) were well balanced across arms of the trial (see [10]).

3.2 Resource use

The UCS has a wide range of offerings: use of the most common individual offerings (individual assessment and individual counselling sessions) and aggregates across the different types of UCS offerings between T0 and T2 are compared by arm in Table 2. Relatively small proportions in each arm used routine UCS offerings: 16% in MSS + SAU and 18% in SAU, a non-significant difference ($p = 0.5199$). Compared to SAU, the MSS + SAU arm used on average fewer routine UCS offerings, but none of the differences were significant ($p > 0.18$). More detailed resource use comparisons between arms are given in Supplementary materials Section 6.4.

3.3 Unit costs

Hourly costs (including employer on-costs) of employing grade 4 and 7 staff respectively are (see Supplementary materials Section 6.5 for detailed information): £17.71 and £25.45. Unit costs for all UCS offerings are given in Section 6.5. The two most commonly delivered UCS activities are individual assessment and counselling sessions, both delivered by grade 7 staff, with estimated unit costs per student respectively of: £42.42 and £27.57. The resources and costs involved in delivering a course (for 30 students) of mindfulness are given in Table 3; the resulting cost (£52.82) per student is included as a cost for all MSS+SAU students.

3.4 Costs

Aggregate costings per student for UCS and mindfulness course use from T0 to T2 by arm are given in Table 4; detailed costing by each activity are given in Supplementary materials Section 6.6. Excluding costs of the mindfulness course, costs are lower in the MSS + SAU arm, but not significantly so ($p > 0.18$). Overall, mean costs in the MSS + SAU arm (£66.91) are significantly ($p < 0.0001$) higher than in the SAU (£19.42), and this is primarily driven by the costs of the mindfulness course.

For peer review only - http://bmjopen.bmj.com/site/about/guidelines.xhtml

Table 1: Univariate comparisons of variables and outcomes by arm. Utilities and QALYs separated by cohort given differing durations to accrue QALYs.

		MSS+SAU (n=309)			SAU (n=307)			
		n	n/Mean	SD	n	n/Mean	SD	P-value
	Gender female (n)	309	187		307	201		0.2115
	Age	306	23	5	306	23	6	0.9219
Michaelmas cohort (172, 170)	Utility T0	172	0.744	0.114	168	0.750	0.121	0.6656
	Utility T1	138	0.782	0.093	135	0.754	0.115	0.0257
	Utility T2	126	0.786	0.098	122	0.735	0.127	0.0005
	Utility T3	92	0.784	0.100	103	0.769	0.122	0.3244
	QALYs T0-T2	116	0.483	0.045	109	0.464	0.058	0.0064
	QALYs T0-T3	86	0.778	0.071	88	0.749	0.094	0.0237
Lent cohort (137, 137)	Utility T0	137	0.767	0.114	136	0.782	0.104	0.2415
	Utility T1	115	0.783	0.101	90	0.771	0.107	0.4313
	Utility T2	109	0.796	0.111	94	0.759	0.112	0.0205
	Utility T3	77	0.794	0.092	66	0.779	0.120	0.4044
	QALYs T0-T2	102	0.316	0.030	85	0.312	0.032	0.3513
	QALYs T0-T3	70	0.853	0.066	60	0.828	0.081	0.0595
CORE-OM mean score	T0	309	1.0	0.5	305	1.0	0.5	0.4102
	T1	255	0.9	0.5	227	1.0	0.5	0.0022
	T2	237	0.9	0.5	216	1.1	0.6	0.0000
	T3	169	0.8	0.5	169	0.9	0.6	0.0312
WEMWBS sum score	T0	307	48.0	8.6	307	48.6	8.5	0.3900
	T1	254	49.6	8.9	221	46.9	9.0	0.0009
	T2	235	48.9	9.0	214	46.4	9.1	0.0031
	T3	168	51.1	9.6	167	48.8	8.9	0.0243

For peer review only - http://bmjopen.bmj.com/site/about/guidelines.xhtml

Table 2: Aggregated UCS resource use by arm between T0 to T2. Means are the amount of each activity by student. SD= standard deviation

Resource use T0 to T2	MSS+SAU (n=309) n/Mean	MSS+SAU (n=309) SD	SAU (n=307) n/Mean	SAU (n=307) SD	P-value
Used UCS services: N=	49		55		
Individual assessment sessions	0.09	0.30	0.11	0.33	0.5937
Individual counselling sessions	0.30	1.31	0.47	1.70	0.1875
Total other individual activity	0.03	0.18	0.05	0.41	0.3637
Total workshops	0.07	0.33	0.04	0.25	0.2748
Total groups	0.03	0.16	0.02	0.14	0.5978

Table 3: Resources and costs used to deliver a course of mindfulness, with capacity for 30 students.

Activities	Times per course	Duration	Staff grade	Cost (£)
Administrative support	1	23.25 hours	4	407
Mindfulness teacher project management	1	20.8 hours	7	509
Venue preparation	8	60 mins	7	204
Pre-session individual student contact time	8	15 mins	7	51
Group session - 1st	1	90 mins	7	38
Group session - 2nd-8th	7	75 mins	7	223
Post-session individual student contact time	8	15 mins	7	51
Post-session note recording	8	15 mins	7	51
Venue `tidy-up'	8	15 mins	7	51
Total				1,584
Total by student (n=30)				52.82

For peer review only - http://bmjopen.bmj.com/site/about/guidelines.xhtml

Table 4: Mean cost (per student) incurred by the UCS. SD= standard deviation

	MSS+SAU (n=309)		SAU (n=307)		
Costs T0 to T2	n/Mean (£)	SD (£)	n/Mean (£)	SD (£)	P-value
Individual assessment sessions	3.98	12.85	4.56	14.02	0.5937
Individual counselling sessions	8.39	36.15	12.84	46.92	0.1875
Total other individual activity	0.60	5.00	1.24	11.33	0.3712
Total workshops	0.30	1.49	0.16	0.94	0.1781
Total groups	0.83	6.12	0.62	4.77	0.6288
MSS course	52.82	0.00	0.00	0.00	
Total	66.91	46.28	19.42	56.35	0.0000

3.5 Outcomes

Unadjusted outcome comparisons are reported in Table 1.

Given the different periods each cohort has to accrue QALYs, we consider utilities and QALYs separately by cohort. Utilities are plotted by arm and cohort in Supplementary materials Figure 6.4 (within each cohort/arm group, N varies by time point – see Table 1). Irrespective of cohort, utilities for MSS + SAU (black) are higher (better) at T1-T3 than SAU (red); they are significantly higher at T2 (the primary outcome time-point) for both cohorts ($p < 0.02$). Compared to SAU, MSS + SAU accrued more QALYs by both T2 and T3, but the means were significantly ($p = 0.01$ and $p = 0.02$ respectively) higher in the Michaelmas cohort; in the Lent cohort, MSS+SAU arm approached significantly ($p = 0.06$) higher QALYs at T3.

At baseline (T0), CORE-OM mean scores were similar between arms, but for all subsequent points (T1-T3), MSS + SAU had significantly ($p < 0.03$) lower (better) mean scores than SAU. Mean WEMWBS sum-scores were similar at baseline (T0), but MSS + SAU scores were all significantly ($p < 0.02$) better (higher) than SAU (T1-T3).

3.6 Cost-effectiveness analysis

Estimates of the adjusted mean incremental costs and mean incremental outcomes (QALYs, CORE-OM improvement or WEMWBS improvement) generated from the bivariate regressions are shown in Table 5, alongside the corresponding ICERs (note that CORE-OM scores have been reversed, so direction of improvement aligns with other outcomes). CEACs for the base-case and scenarios S1-5 are shown in Figure 2.

For peer review only - http://bmjopen.bmj.com/site/about/guidelines.xhtml

In the base-case and all scenarios, MSS+SAU achieves significantly (95% CIs exclude zero) higher (better) outcomes, but is also significantly more expensive, than SAU. In this setting, there are no established thresholds for what ICERs would be considered cost-effective. However, at NICE threshold of £20,000 per QALY, used in economic evaluations for the NHS, MSS+SAU would be considered cost-effective for the base-case and scenarios S1-3 (where outcomes are measured in terms of QALYs).

The uncertainty surrounding cost-effectiveness conclusions are depicted in the cost-effectiveness planes in Supplementary materials Section 6.10. All bootstrap replicates fell in the north-east quadrant for the base case and all scenarios S1-5: this means that when evaluating uncertainty using bootstrap methods, *all* bootstrap replicates indicated the MSS+SAU was both more costly and more effective than SAU.

The probability of cost-effectiveness, illustrated by the CEACs (Figure 2), rapidly moves to near certainty as the willingness-to-pay thresholds exceeds £6,000 per outcome unit (base case and S1-S3: QALYs; S4: CORE-OM unit; S5: WEMWBS unit). Considering the base case and scenarios S1-S3 at the NICE thresholds noted above, there is near certainty of MSS+SAU being more cost-effective than SAU: there is very little probability of making the wrong decision about cost-effectiveness at this threshold.

Table 5: UCS costs (£), outcomes (base case and S1-S3: QALYs; S4: CORE-OM unit; S5: WEMWBS unit) and ICER for base-case and scenarios, where SAU is the reference. Costs and outcomes adjusted for sex and cohort; additionally, outcomes adjusted by corresponding baseline outcome measurement. CORE-OM scores reversed to align direction of improvement with other outcomes: an increase in CORE-OM here indicates an improvement (reduction) on the original scale. Incre. dif. = incremental difference. CI = confidence interval.

	Costs (£)			Outcome			
Scenario	Incre. dif.	95% CI		Incre. dif.	95% CI		ICER
Base case	48	40	56	0.0143	0.0079	0.0207	3,355
S1: CCA	54	45	63	0.0140	0.0073	0.0208	3,842
S2: Per-protocol	50	40	60	0.0160	0.0090	0.0229	3,112
S3: Up to T3 (1 year)	47	35	58	0.0305	0.0179	0.0432	1,525
S4: CORE-OM T2 CE	48	40	56	0.3	0.3	0.2	188
S5: WEMWBS T2 CE	48	40	56	3.0	1.6	4.5	16

For peer review only - http://bmjopen.bmj.com/site/about/guidelines.xhtml

4 Discussion

4.1 Main findings

There is strong evidence from here and elsewhere [10,11] that MSS leads to significantly improved outcomes. These improved outcomes come at a moderate cost: a mean additional cost of around £50 per user if mindfulness is delivered as it was here, with 30 participants per group. There are no agreed thresholds for this setting to determine whether the gains and additional costs would be considered cost-effective; however, compared to NICE thresholds (eg £20,000/QALY), MSS+SAU would be considered cost-effective compared to SAU. The uncertainty around this decision again depends on the chosen threshold: at NICE thresholds, there is near certainty that MSS+SAU is cost-effective; also, near certainty is reached for what most would likely consider low willingness-to-pay values (see Figure 2).

4.2 Comparison with other studies

Systematic reviews of mindfulness based interventions for students have broadly been in favour of mindfulness improving student mental health [35–37]. Evaluations of such interventions have generally been in RCTs, but have been noted to vary in quality, typically with a high risk of bias, limited follow-up and with little consideration of safety [35–37]. In contrast, the RCT from which this economic evaluation draws data has a lower than average risk of bias, followed students for up to 12 months, and reported on safety [10].

Turning to economic evaluations of mindfulness based interventions, we are unaware of any others in university settings. Recent systematic reviews of economic evaluations [38,39] of mindfulness-related interventions show a near exclusive focus on clinical populations, particularly for relapse prevention in major depression. These reviews consider a range of intervention which incorporate mindfulness training to varying degrees; most comparable to this trial's intervention are mindfulness-based cognitive therapy (MBCT) and mindfulness-based stress reduction (MBSR). Both reviews conclude similarly for MBCT that 'findings are inconclusive because of the presence of very positive results in some trials … and modest results in others' (p.143) [38]. There was only one economic evaluation of an MBSR intervention [40], with an ICER of $22,200/QALY (international dollars at 2014 prices) which the authors considered within the threshold of cost-effectiveness.

We are aware of only two articles that explore the economic impact of mindfulness outside of clinical populations [41,42]. Dongel et al. [41] conducted a 'cost-effectiveness analysis and return-on-investment analysis comparing mindfulness based work site intervention to usual practice' (p.550) for governmental research institute employees. Usual practice 'dominated' the mindfulness intervention: for all outcomes considered (work engagement, general vitality and work ability) scores were statistically significant in favour of usual practice, and costs lower for this group from both employer and societal perspectives. Accordingly, the return on investment was negative. These negative findings are suggested to be due to the 'participants' low compliance with some of the intervention components'

For peer review only - http://bmjopen.bmj.com/site/about/guidelines.xhtml

(p.555). MSS participants' intervention attendance was not dissimilar to theirs, although MSS participants appear to have been slightly more compliant with homework [10].

Over five years, Klatt et al. [42] compares healthcare costs (to insurers) and utilisation by university staff who received one of two interventions (one of them mindfulness) compared to matched controls: compared to controls, costs were lower for both of the interventions. There was no economic evaluation in the original trial [43], but the interventions were suggested to be 'low-cost' [43] while unlikely to be funded by the insurer [42].

4.3 Study strengths and weaknesses

Contrasting with many studies in this area (see Section 4.2, a key strength of this economic evaluation is that it drew on data from a large ($n > 600$) RCT that followed-up participants for up to 12 months. Additionally, to our knowledge, it is the only economic evaluation of a mindfulness intervention for students. The choice of measures used allowed QALYs to be calculated.

A key weakness of the study is the very narrow costing perspective adopted – that of the UCS. This perspective likely captures the majority of costs of directly delivering the intervention, but may well miss wider impacts. A natural perspective to adopt would be that of the university, but collecting required information for such a wide and multi-domain perspective (for example, details on tuition fees and use of other student support services) would be very challenging.

A further weakness is that in costing we have focused exclusively on the costs of staff time, as measured by salary plus 'on-costs (e.g. such as employers' pension and national insurance costs – see £ Section 6.5). We have not considered wider overheads or other resources (such as room hire), which will underestimate costs. In contrast to the NHS (for instance [44]), we are not aware of any pre-existing cost sources. A potentially key cost exclusion, particularly given the group based nature of the intervention, is the cost of room hire. While the UCS' operating budget does not incur a cost for room hire, there is of course a potential 'opportunity cost', by which we mean that if the rooms were not being used for MSS, they could potentially be used for something else, such as teaching. We would expect the opportunity cost to be lower where there is less 'competition' for rooms - for example, if mindfulness were delivered in the evenings, when rooms are not required for teaching.

The primary analysis was a cost-utility analysis [28] where the ICER corresponds to cost per additional QALY. Interpreting the ICER is difficult in this sector as there are no established cost-effectiveness thresholds (see Section 2.4). As argued by [45], policy makers may benefit from a different analysis such as cost-benefit analysis (CBA) which 'is a comparison of interventions and their consequences in which both costs and resulting benefits (health outcomes and others) are expressed in monetary terms' [46]. Alternatives can then compared using the net monetary benefit 'which is the difference between the benefit of each treatments (expressed in monetary units) less the cost of each' [46]. However, valuing benefits in monetary terms as required by CBA is demanding, and arguably particularly so in this setting where benefits occur in many diverse domains.

For peer review only - http://bmjopen.bmj.com/site/about/guidelines.xhtml

5 Conclusions

This economic evaluation has shown MSS+SAU accrue more benefits than SAU, but these increased benefits come at a greater cost. The main driver of these increased costs were the costs of delivering the MSS course. Whether these benefits are considered to be worth the additional cost depends on the cost-effectiveness threshold adopted - this is not established for the university sector. Compared to the NICE threshold for the NHS, MSS+SAU would be considered highly cost-effective compared to SAU.

Word count: 4,196

Contributorship statement

GD conceived the mindfulness intervention pilot on behalf of the UCS and concurrent research into the effectiveness of the provision. PBJ and GD applied for funding for the underlying randomised controlled trial. JG and PBJ produced an initial draft of the overall study protocol that was revised through discussion with all the authors. APW and GB produced an initial draft of the health economic plan which was revised through discussion with all authors. JG, GD, MV, and PBJ did the study, with input from the other authors. APW led the health economic analysis, with input from JG, GB & JS. APW led drafting of this article, which was read, reviewed & approved by the other authors. PBJ is study guarantor.

Competing interests

The authors have no conflicts of interest to declare.

Funding

This is a summary of research funded by the University of Cambridge Vice-Chancellor's Endowment Fund, the University Counselling Service and the National Institute for Health and Care Research (NIHR) Applied Research Collaboration East of England (ARC EoE) programme. The views expressed are those of the authors and not necessarily those of the University of Cambridge, NHS, NIHR or Department of Health and Social Care.

Data sharing statement

De-identified individual participant data and dictionary are available for researchers upon request from the corresponding author after approval of a proposal, with a signed data access agreement.

Ethics approval statement

This study was approved by the Cambridge Psychology Research Ethics committee on August 25, 2015 (reference: PRE.2015.060).

Acknowledgements

We thank the study participants, the mindfulness teacher Elizabeth English for her development of the intervention independently of the researchers, the administrative and senior management team at the UCS, Alice Benton, and Emma Howarth.

For peer review only - http://bmjopen.bmj.com/site/about/guidelines.xhtml

References

1 Mair D. Short-term counselling in higher education—context, theory and practice. Abingdon: Routledge 2016. 7–26.

2 Thorley C. Not by degrees: Improving student mental health in the UK's universities. 2017. https://www.ippr.org/research/publications/not-by-degrees

3 Rith-Najarian LR, Boustani MM, Chorpita BF. A systematic review of prevention programs targeting depression, anxiety, and stress in university students. *Journal of Affective Disorders* 2019;**257**:568–84. http://www.sciencedirect.com/science/article/pii/S016503271832754X

4 Lattie EG, Lipson SK, Eisenberg D. Technology and college student mental health: Challenges and opportunities. *Frontiers in psychiatry* 2019;**10**:246–6. https://www.ncbi.nlm.nih.gov/pmc/articles/PMC6476258/

5 Kabat-Zinn J. Mindfulness-based interventions in context: Past, present, and future. *Clinical Psychology: Science and Practice* 2003;**10**:144–56. doi:10.1093/clipsy.bpg016

6 Mindfulness for Students. Mindfulness courses at UK universities. 2018. http://mindfulnessforstudents.co.uk/mindfulness-courses-at-uk-universities

7 Mindfulness All-Party Parliamentary Group. Mindful Nation UK Report: The Mindfulness Initiative. 2015. https://www.themindfulnessinitiative.org/mindful-nation-report

8 Dufour G. Current challenges in student mental health and counselling provision: How practice-based research can help demonstrate effectiveness. *Counselling and Psychotherapy Research* 2020;1–6. doi:10.1002/capr.12310

9 Galante J, Dufour G, Benton A, *et al.* Protocol for the mindful student study: A randomised controlled trial of the provision of a mindfulness intervention to support university students' well-being and resilience to stress. *BMJ Open* 2016;**6**:e012300. doi:10.1136/bmjopen-2016-012300

10 Galante J, Dufour G, Vainre M, *et al.* A mindfulness-based intervention to increase resilience to stress in university students (the mindful student study): A pragmatic randomised controlled trial. *The Lancet Public Health* 2018;**3**:e72–81. doi:10.1016/s2468-2667(17)30231-1

11 Galante J, Stochl J, Dufour G, *et al.* Effectiveness of providing university students with a mindfulness-based intervention to increase resilience to stress: 1-year follow-up of a pragmatic randomised controlled trial. *Journal of Epidemiology & Community Health* 2021;**75**:151–60. doi:10.1136/jech-2020-214390

12 Wagner AP, Barton G. Mindfulness student study (MSS): Health economic analysis plan (HEAP). University of East Anglia; Internet 2018.

For peer review only - http://bmjopen.bmj.com/site/about/guidelines.xhtml

https://www.anzctr.org.au/Steps11and12/369222-(Uploaded-23-08-2019-20-02-35)-Study-related document.pdf

13 CORE System Group. CORE system user manual. 2015. http://www.coreims.co.uk/index.html

14 Evans C, Connell J, Barkham M, *et al.* Towards a standardised brief outcome measure: Psychometric properties and utility of the CORE-OM. *The British journal of psychiatry : the journal of mental science* 2002;**180**:51–60.

15 Connell J, Barkham M, Mellor-Clark J. The effectiveness of UK student counselling services: An analysis using the CORE system. *British Journal of Guidance & Counselling* 2008;**36**:1–8. doi:10.1080/03069880701715655

16 York Health Economics Consortium. Preference-based measures. 2016. https://yhec.co.uk/glossary/preference-based-measures/

17 Mavranezouli I, Brazier JE, Rowen D, *et al.* Estimating a preference-based index from the clinical outcomes in routine evaluation-outcome measure (CORE-OM): Valuation of CORE-6D. *Medical decision making : an international journal of the Society for Medical Decision Making* 2013;**33**:381–95. https://www.ncbi.nlm.nih.gov/pmc/articles/PMC4107796/

18 Mavranezouli I, Brazier JE, Young TA, *et al.* Using rasch analysis to form plausible health states amenable to valuation: The development of CORE-6D from a measure of common mental health problems (CORE-OM). *Quality of life research* 2011;**20**:321–33.

19 Manca A, Hawkins N, Sculpher MJ. Estimating mean QALYs in trial-based cost-effectiveness analysis: The importance of controlling for baseline utility. *Health economics* 2005;**14**:487–96.

20 Stewart-Brown S, Janmohamed K. Warwick-Edinburgh Mental Well-Being Scale (WEMWBS). User guide: version 1. 2008. http://www.mentalhealthpromotion.net/resources/user-guide.pdf

21 Tennant R, Hiller L, Fishwick R, *et al.* The warwick-edinburgh mental well-being scale (WEMWBS): Development and UK validation. *Health and quality of life outcomes* 2007;**5**:63.

22 Noble SM, Hollingworth W, Tilling K. Missing data in trial-based cost-effectiveness analysis: The current state of play. *Health Econ* 2012;**21**:187–200. doi:10.1002/hec.1693

23 Faria R, Gomes M, Epstein D, *et al.* A guide to handling missing data in cost-effectiveness analysis conducted within randomised controlled trials. *PharmacoEconomics* 2014;**32**:1157–70.

24 White IR, Royston P, Wood AM. Multiple imputation using chained equations: Issues and guidance for practice. *Statist Med* 2011;**30**:377–99. doi:10.1002/sim.4067

For peer review only - http://bmjopen.bmj.com/site/about/guidelines.xhtml

25 van Buuren S, Groothuis-Oudshoorn K. mice: Multivariate imputation by chained equations in r. *Journal of Statistical Software* 2011;**45**:1–67. https://www.jstatsoft.org/v45/i03/

26 Little RJA, Rubin DB. *Statistical analysis with missing data*. 2nd ed. Hoboken, NJ: : Wiley 2002.

27 Willan AR, Briggs AH, Hoch JS. Regression methods for covariate adjustment and subgroup analysis for non-censored cost-effectiveness data. *Health economics* 2004;**13**:461–75.

28 Drummond MF, Sculpher MJ, Claxton K, *et al. Methods for the economic evaluation of health care programmes*. 4th ed. Oxford University Press, Oxford 2015.

29 NICE. NICE - the national institute for HEalth and care excellence. 2020.

30 NICE. *Guide to the methods of technology appraisal*. National Institute of Health; Clincal Excellence, London 2013.

31 Briggs AH, O'Brien BJ, Blackhouse G. Thinking outside the box: Recent advances in the analysis and presentation of uncertainty in cost-effectiveness studies. *Annual review of public health* 2002;**23**:377–401.

32 Black WC. The CE plane: A graphic representation of cost-effectiveness. *Medical decision making : an international journal of the Society for Medical Decision Making* 1990;**10**:212–4.

33 Fenwick E, O'Brien BJ, Briggs A. Cost-effectiveness acceptability curves–facts, fallacies and frequently asked questions. *Health economics* 2004;**13**:405–15.

34 R Core Team. *R: A language and environment for statistical computing*. Vienna, Austria: : R Foundation for Statistical Computing 2019. https://www.R-project.org/

35 Chiodelli R, Mello LTN de, Jesus SN de, *et al.* Mindfulness-based interventions in undergraduate students: A systematic review. *Journal of American College Health* 2020;1–0. doi:10.1080/07448481.2020.1767109

36 Breedvelt JJF, Amanvermez Y, Harrer M, *et al.* The effects of meditation, yoga, and mindfulness on depression, anxiety, and stress in tertiary education students: A meta-analysis. *Frontiers in psychiatry* 2019;**10**:193–3. https://www.ncbi.nlm.nih.gov/pmc/articles/PMC6491852/

37 Dawson AF, Brown WW, Anderson J, *et al.* Mindfulness-based interventions for university students: A systematic review and meta-analysis of randomised controlled trials. *Applied psychology Health and well-being* 2019.

38 Duarte R, Lloyd A, Kotas E, *et al.* Are acceptance and mindfulness-based interventions "value for money"? Evidence from a systematic literature review. *British Journal of Clinical Psychology* 2019;**58**:187–210. doi:10.1111/bjc.12208

For peer review only - http://bmjopen.bmj.com/site/about/guidelines.xhtml

39 Feliu-Soler A, Cebolla A, McCracken LM, *et al.* Economic impact of third-wave cognitive behavioral therapies: A systematic review and quality assessment of economic evaluations in randomized controlled trials. *Behavior Therapy* 2018;**49**:124–47. http://www.sciencedirect.com/science/article/pii/S000578941730076X

40 Lengacher CA, Kip KE, Reich RR, *et al.* A cost-effective mindfulness stress reduction program: A randomized control trial for breast cancer survivors. *Nursing economic* 2015;**33**:210–8, 232.

41 Dongen JM van, Berkel J van, Boot CRL, *et al.* Long-term cost-effectiveness and return-on-investment of a mindfulness-based worksite intervention: Results of a randomized controlled trial. *Journal of occupational and environmental medicine* 2016;**58**:550–60.

42 Klatt MD, Sieck C, Gascon G, *et al.* A healthcare utilization cost comparison between employees receiving a worksite mindfulness or a diet/exercise lifestyle intervention to matched controls 5 years post intervention. *Complementary therapies in medicine* 2016;**27**:139–44.

43 Malarkey WB, Jarjoura D, Klatt M. Workplace based mindfulness practice and inflammation: A randomized trial. *Brain, behavior, and immunity* 2013;**27**:145–54.

44 Curtis L, Burns A, editors. *Unit costs of health and social care 2019*. Canterbury: : Personal Social Services Research Unit, University of Kent 2018. doi:10.22024/UniKent/01.02.79286

45 Tudor Edwards R, Bryning L, Crane R. Design of economic evaluations of mindfulness-based interventions: Ten methodological questions of which to be mindful. *Mindfulness* 2015;**6**:490–500. https://www.ncbi.nlm.nih.gov/pmc/articles/PMC4432017/

46 York Health Economics Consortium. Cost-benefit analysis. 2016. https://yhec.co.uk/glossary/cost-benefit-analysis/

47 Moher D, Hopewell S, Schulz KF, *et al.* CONSORT 2010 explanation and elaboration: Updated guidelines for reporting parallel group randomised trials. *BMJ* 2010;**340**:c869. doi:10.1136/bmj.c869

For peer review only - http://bmjopen.bmj.com/site/about/guidelines.xhtml

Figure captions

Figure 1: Participant timeline. Reproduced from Galante et al. (2016) [9].

Figure 2: Cost-effectiveness acceptability curves (CEACs) for the base-case and scenario analyses: each curve shows the probability of cost-effectiveness at a particular willingness-to-pay (λ) threshold per unit of outcome (QALYs: base case, S1-3; CORE-OM point: S4; WEMWBS point: S5).

For peer review only

For peer review only - http://bmjopen.bmj.com/site/about/guidelines.xhtml

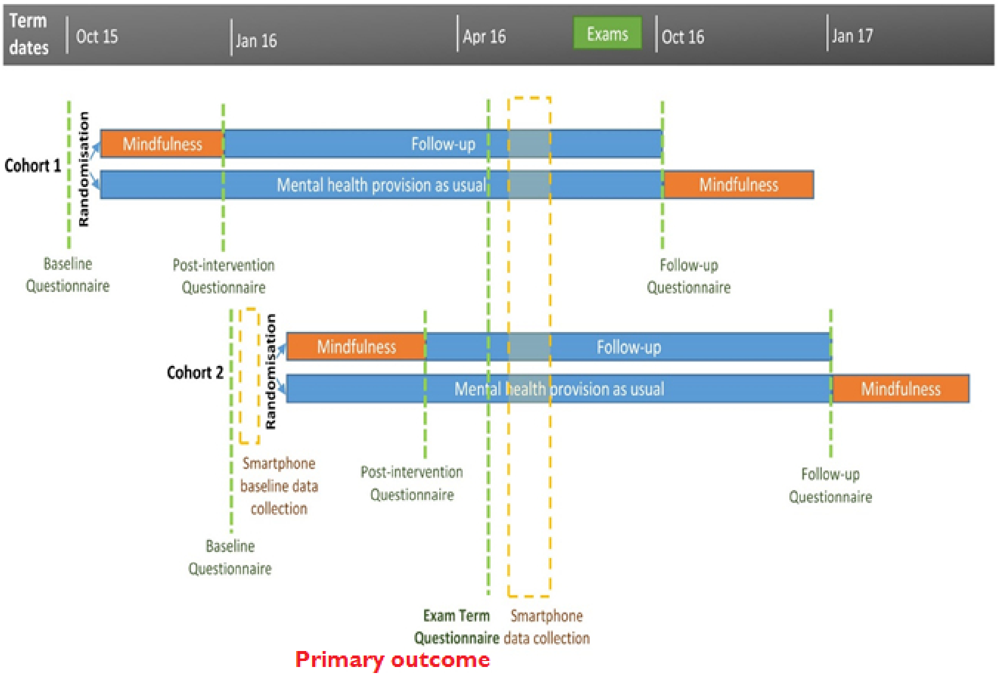

Participant timeline. Reproduced from Galante et al. (2016) [9]

451x306mm (38 x 38 DPI)

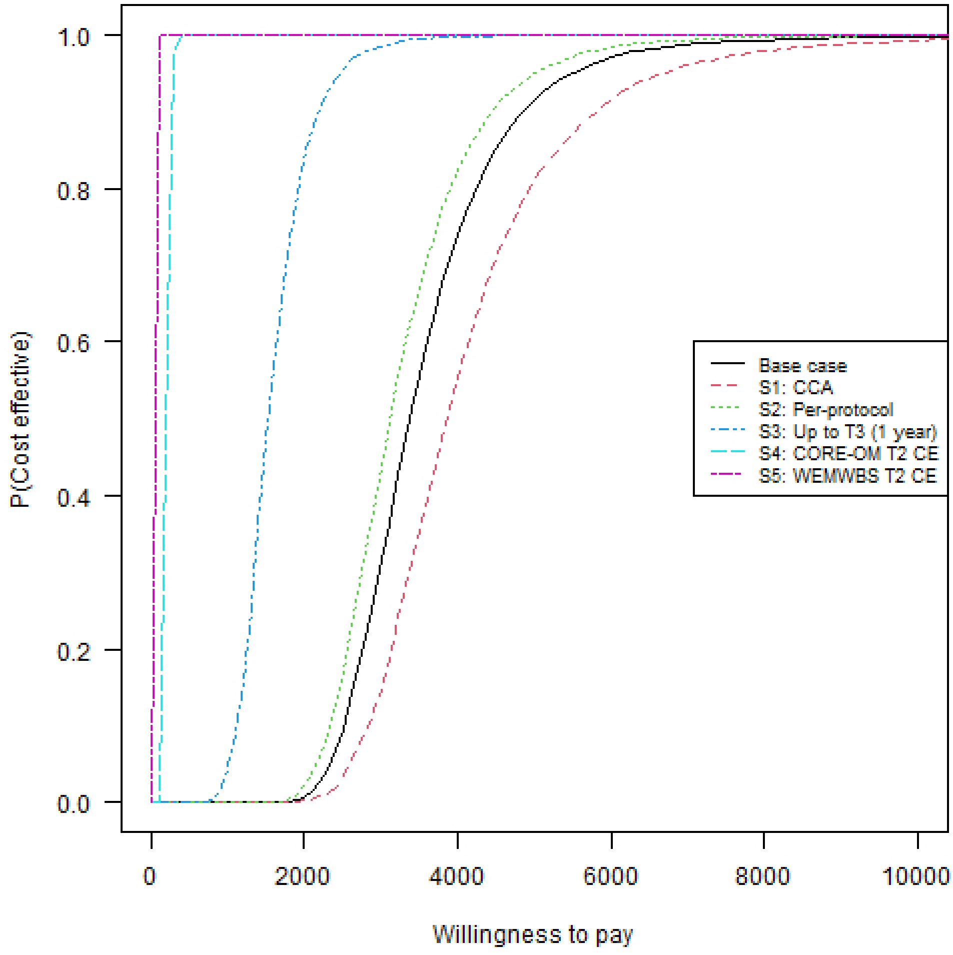

Cost-effectiveness acceptability curves (CEACs) for the base-case and scenario analyses: each curve shows the probability of cost-effectiveness at a particular willingness-to-pay (λ) threshold per unit of outcome (QALYs: base case, S1-3; CORE-OM point: S4; WEMWBS point: S5).

169x169mm (72 x 72 DPI)

Cost-effectiveness of providing university students with a mindfulness-based intervention to increase resilience to stress: economic evaluation of a pragmatic randomised controlled trial

SUPPLEMENTARY MATERIALS

Adam P Wagner, Julieta Galante, Géraldine Dufour, Garry Barton, Jan Stochl, Maris Vainre, and Peter B Jones

19/01/2023

Adam P Wagner, PhD, (Corresponding author) Senior Research Fellow, Norwich Medical School, University of East Anglia, Norwich Research Park, Norwich, NR4 7TJ, United Kingdom (second affiliation: National Institute for Health Research (NIHR) Applied Research Collaboration (ARC) East of England (EoE)). adam.wagner@uea.ac.uk

Julieta Galante, PhD, Honorary Senior Visiting Research Fellow, Department of Psychiatry, University of Cambridge, Douglas House, 18b Trumpington Road, Cambridge, CB2 8AH, United Kingdom (second affiliation: Contemplative Studies Centre, Melbourne School of Psychological Sciences, Faculty of Medicine, Dentistry, and Health Sciences, University of Melbourne). mjg231@cam.ac.uk phone (+44) 7986 172834

Géraldine Dufour, MA, Geraldine Dufour Therapeutic Consultations Ltd https://www.geraldinedufour.com/ (second affiliation: Associate Executive Member, European Association for International Education (EAIE)).

Garry Barton, PhD, Professor of Health Economics, Norwich Medical School, University of East Anglia, Norwich Research Park, Norwich, NR4 7TJ, United Kingdom.

Jan Stochl, PhD, Senior Research Associate, Department of Psychiatry, University of Cambridge, Cambridge Biomedical Campus, Box 189, Cambridge, CB2 0QQ, United Kingdom (second affiliation: Department of Kinanthropology, Charles University, Jose Martiho 31, 162 52 Prague, Czech Republic; third affiliation: NIHR ARC EoE).

Maris Vainre, MA, PhD Student, MRC Cognition and Brain Sciences Unit, University of Cambridge, 15 Chaucer Rd, Cambridge, CB2 7EF, United Kingdom (former affiliation when doing this work: NIHR ARC EoE). Maris.Vainre@mrc-cbu.cam.ac.uk

Peter B Jones, PhD, Professor of Psychiatry, Department of Psychiatry, University of Cambridge, Herchel Smith Building, Cambridge, CB2 0SZ, United Kingdom (second affiliation: NIHR ARC EoE).

For peer review only - http://bmjopen.bmj.com/site/about/guidelines.xhtml

6 Supplementary materials

6.1 Missingness by scale and time point

Table 6.1: Missingness (percentage) by outcomes at each time, for each arm and overall.

	Percentage missing		
Outcome	MSS+SAU	SAU	Overall
CORE-6D, T0	0	1	0
CORE-6D, T1	18	27	22
CORE-6D, T2	24	30	27
CORE-6D, T3	45	45	45
CORE-OM, T0	3	4	3
CORE-OM, T1	19	28	23
CORE-OM, T2	26	30	28
CORE-OM, T3	46	46	46
WEMWBS, T0	1	0	0
WEMWBS, T1	18	28	23
WEMWBS, T2	24	30	27
WEMWBS, T3	46	46	46

For peer review only - http://bmjopen.bmj.com/site/about/guidelines.xhtml

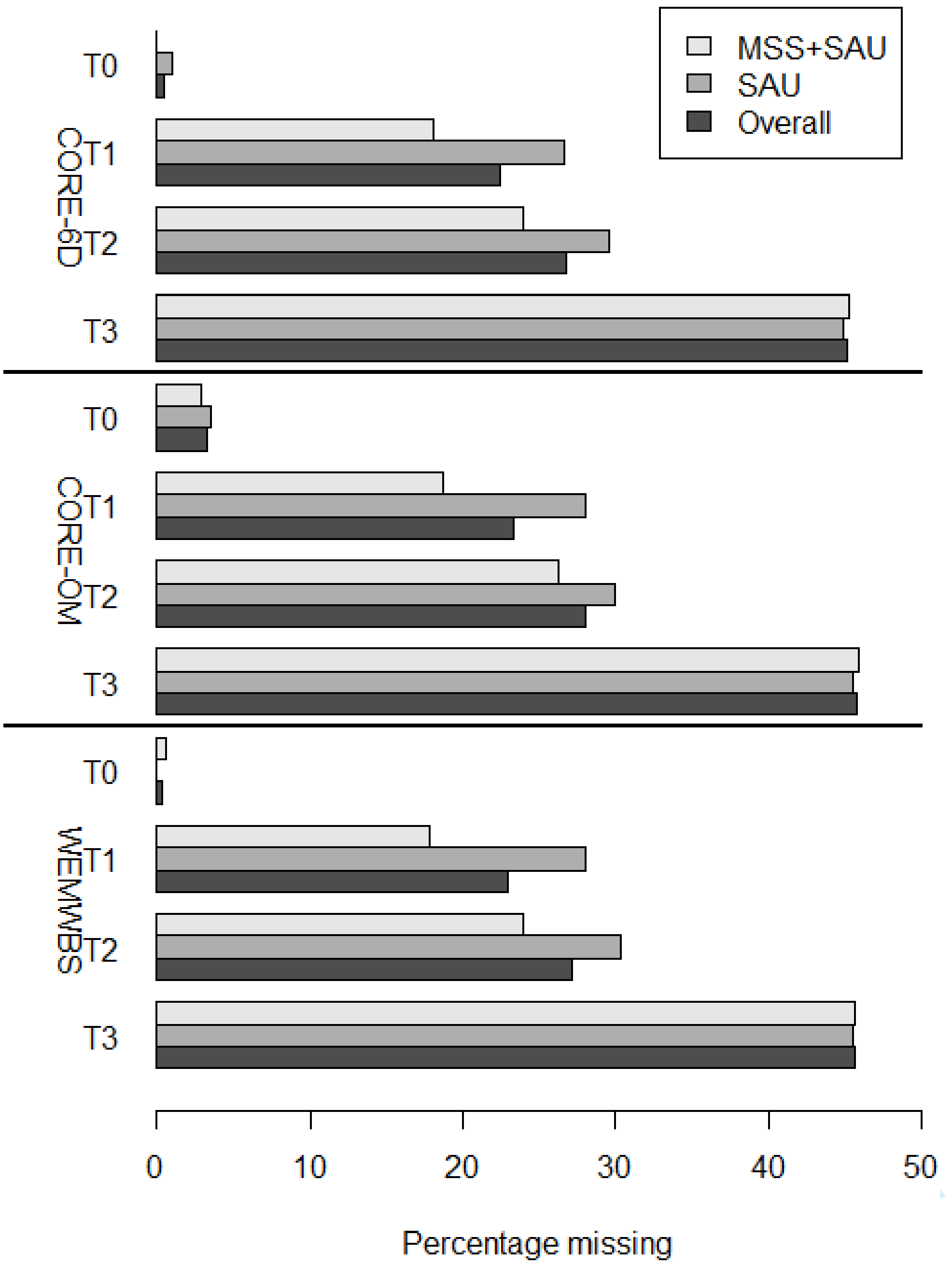

Figure 6.1: Missingness (percentage) by outcomes at each time, for each arm and overall.

6.2 Patterns of missingness and imputation model

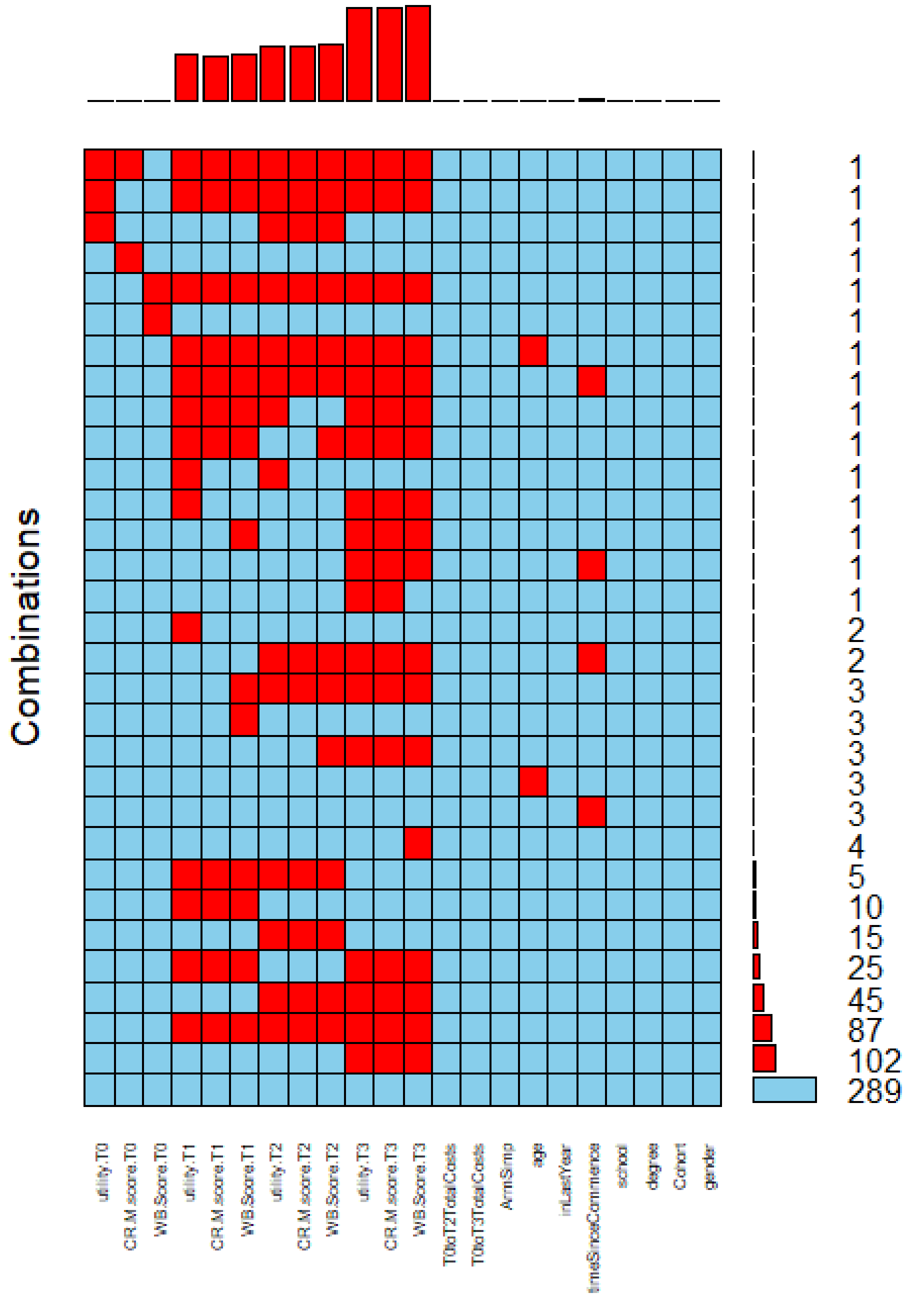

Figure 6.2: Patterns of missingness (red indicates missing) and variables included in imputation model.

6.3 Trial CONSORT diagram

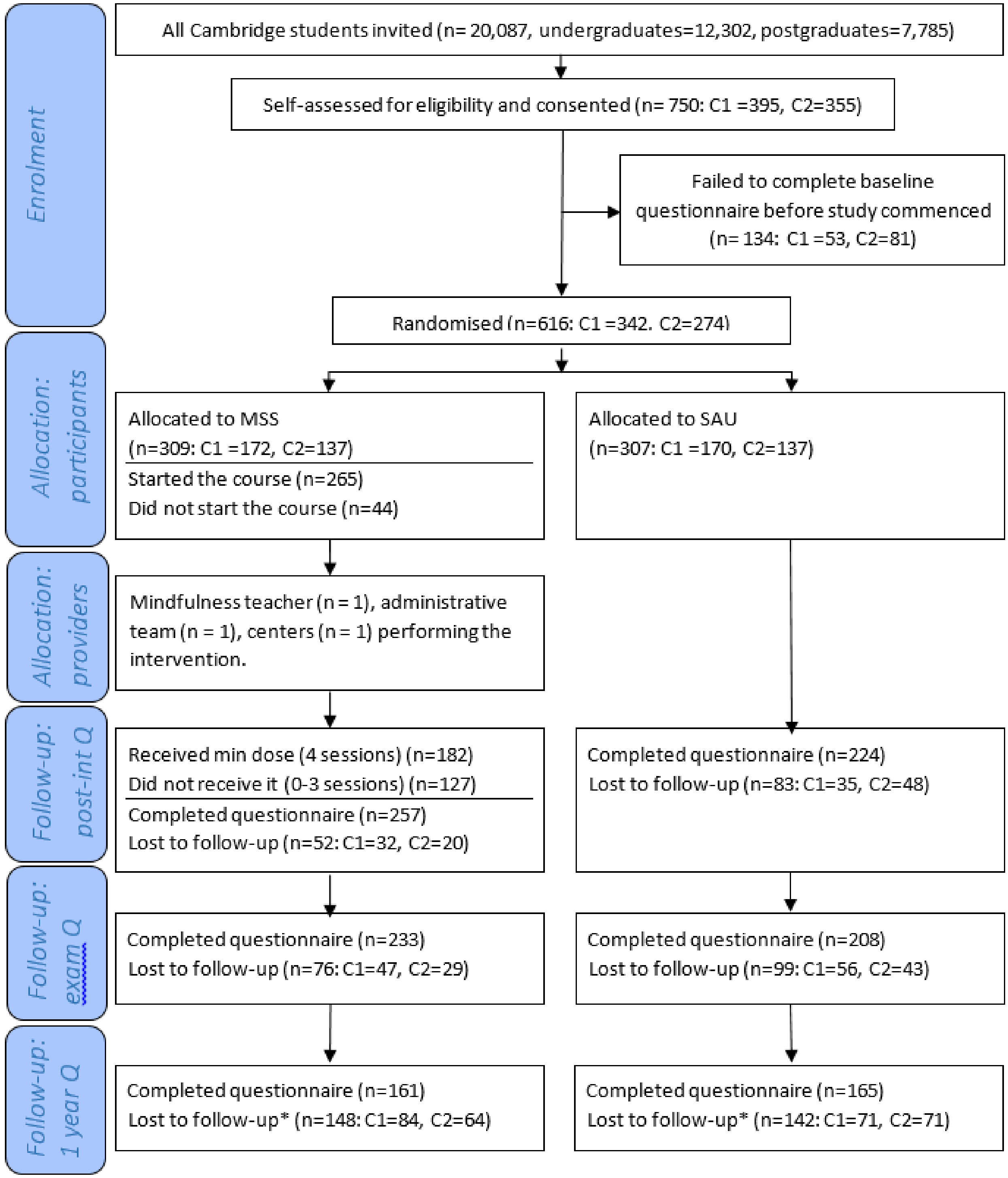

Figure 6.3: CONSORT 2010 [47] diagram taken from Galante et al. (2021) [11]

6.4 Resource use T0 to T2: Further detail

Table 6.2: Resource use across all participants, T0 to T2 (mean use per student)

	MSS+SAU (n=309)		SAU (n=307)		
Resource use T0 to T2	n/Mean	SD	n/Mean	SD	P-value
Assessment	0.09	0.30	0.11	0.33	0.5937
Counselling	0.30	1.31	0.47	1.70	0.1875
Client Contact by Phone or Email	0.00	0.00	0.00	0.00	-
MHA Assessment	0.01	0.08	0.01	0.08	0.9948
MHA Ongoing	0.00	0.06	0.03	0.36	0.2741
Pre Group	0.02	0.13	0.02	0.13	0.9918
Can't Work Group	0.01	0.08	0.00	0.00	0.1576
Returners - Anxiety	0.00	0.00	0.00	0.00	-
Returners - Self Compassion	0.00	0.00	0.00	0.00	-
Returners - Time Management	0.00	0.00	0.00	0.00	-
Workshop - Anxiety	0.01	0.10	0.01	0.08	0.6591
Workshop - CBT for self-help	0.00	0.06	0.00	0.06	0.9963
Workshop - Exam Preparation	0.00	0.06	0.00	0.06	0.9963
Workshop - Food and mood	0.01	0.11	0.00	0.00	0.0453
Workshop - Procrastination	0.01	0.11	0.02	0.13	0.7302
Workshop - Self-compassion	0.01	0.11	0.01	0.14	0.9934
Workshop - Sleep	0.00	0.00	0.00	0.00	-
Workshop - Social anxiety	0.00	0.00	0.00	0.00	-
Workshop - Panic attacks	0.01	0.08	0.00	0.00	0.1576
Assertiveness Group	0.01	0.08	0.00	0.06	0.5669
Bereavement Group	0.00	0.06	0.01	0.08	0.5601
M Phil Group	0.00	0.00	0.00	0.00	-
Managing Mood Group	0.00	0.06	0.00	0.00	0.3181
Perfectionism Group	0.00	0.00	0.00	0.06	0.3181
Post Graduate Group	0.00	0.00	0.00	0.00	-
Returners Group	0.01	0.08	0.00	0.06	0.5669
Self-esteem Group	0.00	0.06	0.00	0.06	0.9963
UG Group	0.00	0.06	0.00	0.00	0.3181

Table 6.3: Resource use across participants *using* a particular service T0 to T2 (eg means here exclude participants not using that UCS offering)

	MSS+SAU (n=309)		SAU (n=307)	
	Used (N)	Used mean	Used (N)	Used mean
Assessment	28	1.04	31	1.06
Counselling	27	3.48	37	3.86
Client Contact by Phone or Email	0	-	0	-
MHA Assessment	2	1.00	2	1.00
MHA Ongoing	1	1.00	2	4.00
Pre Group	5	1.00	5	1.00
Can't Work Group	2	1.00	0	-
Returners - Anxiety	0	-	0	-
Returners - Self Compassion	0	-	0	-
Returners - Time Management	0	-	0	-
Workshop - Anxiety	3	1.00	2	1.00
Workshop - CBT for self-help	1	1.00	1	1.00
Workshop - Exam Preparation	1	1.00	1	1.00
Workshop - Food and mood	4	1.00	0	-
Workshop - Procrastination	4	1.00	5	1.00
Workshop - Self-compassion	4	1.00	3	1.33
Workshop - Sleep	0	-	0	-
Workshop - Social anxiety	0	-	0	-
Workshop - Panic attacks	2	1.00	0	-
Assertiveness Group	2	1.00	1	1.00
Bereavement Group	1	1.00	2	1.00
M Phil Group	0	-	0	-
Managing Mood Group	1	1.00	0	-
Perfectionism Group	0	-	1	1.00
Post Graduate Group	0	-	0	-
Returners Group	2	1.00	1	1.00
Self-esteem Group	1	1.00	1	1.00
UG Group	1	1.00	0	-

For peer review only - http://bmjopen.bmj.com/site/about/guidelines.xhtml

6.5 Detailed unit costs

Table 6.4: Details of wage costs

Grade	Spine point	Salary (£)	Pension (£)	NI (£)	Apprent levy (£)	Total (£)	Rate (£/hr)
4	32	25,627	5,535	2,487	128	33,777	17.71
7	44	36,382	7,859	4,106	181	48,528	25.45

For peer review only

For peer review only - http://bmjopen.bmj.com/site/about/guidelines.xhtml

Table 6.5: Unit costs for each of the UCS offerings.

Type	Activity	Duration (mins)	Student contact (mins)	Occurrences	Group size	Total cost (£)	Unit cost (£)
Individual	Assessment	100	60	1	1	42.42	42.42
	Counselling	65	60	1	1	27.57	27.57
	Client Contact by Phone or Email	25	20	1	1	10.60	10.60
	MHA Assessment	100	60	1	1	42.42	42.42
	MHA Ongoing	65	60	1	1	27.57	27.57
	Pre Group	35	30	1	1	14.85	14.85
Workshop	Can't Work Group	135	120	1	6	57.26	9.54
	Returners - Anxiety	135	90	1	20	57.26	2.86
	Returners - Self Compassion	135	120	1	20	57.26	2.86
	Returners - Time Management	135	120	1	20	57.26	2.86
	Workshop - Anxiety	135	120	1	15	57.26	3.82
	Workshop - CBT for self-help	135	120	1	15	57.26	3.82
	Workshop - Exam Preparation	135	120	1	15	57.26	3.82
	Workshop - Food and mood	135	120	1	15	57.26	3.82
	Workshop - Procrastination	135	90	1	15	57.26	3.82
	Workshop - Self-compassion	135	120	1	15	57.26	3.82
	Workshop - Sleep	135	90	1	15	57.26	3.82
	Workshop - Social anxiety	135	120	1	15	57.26	3.82
	Workshop - Panic attacks	135	120	1	15	57.26	3.82
Group	Assertiveness Group	135	120	4	8	229.05	28.63
	Bereavement Group	135	120	6	8	343.57	42.95
	M Phil Group	135	120	5	8	286.31	35.79
	Managing Mood Group	135	120	6	8	343.57	42.95
	Perfectionism Group	135	120	5	8	286.31	35.79
	Post Graduate Group	135	120	14	8	801.67	100.21
	Returners Group	135	120	1	20	57.26	2.86
	Self-esteem Group	135	90	5	8	286.31	35.79
	UG Group	135	120	10	8	572.62	71.58

For peer review only - http://bmjopen.bmj.com/site/about/guidelines.xhtml

6.6 Costs T0 to T2: Further detail

Table 6.6: Mean costs per student across all participants, T0 to T2

	MSS+SAU (n=309)		SAU (n=307)		
UCS activity costs T0 to T2	n/Mean (£)	SD (£)	n/Mean (£)	SD (£)	P-value
Assessment	3.98	12.85	4.56	14.02	0.5937
Counselling	8.39	36.15	12.84	46.92	0.1875
Client Contact by Phone or Email	0.00	0.00	0.00	0.00	-
MHA Assessment	0.27	3.41	0.28	3.42	0.9948
MHA Ongoing	0.09	1.57	0.72	9.94	0.2741
Pre Group	0.24	1.88	0.24	1.88	0.9918
Can't Work Group	0.06	0.77	0.00	0.00	0.1576
Returners - Anxiety	0.00	0.00	0.00	0.00	-
Returners - Self Compassion	0.00	0.00	0.00	0.00	-
Returners - Time Management	0.00	0.00	0.00	0.00	-
Workshop - Anxiety	0.04	0.37	0.02	0.31	0.6591
Workshop - CBT for self-help	0.01	0.22	0.01	0.22	0.9963
Workshop - Exam Preparation	0.01	0.22	0.01	0.22	0.9963
Workshop - Food and mood	0.05	0.43	0.00	0.00	0.0453
Workshop - Procrastination	0.05	0.43	0.06	0.48	0.7302
Workshop - Self-compassion	0.05	0.43	0.05	0.53	0.9934
Workshop - Sleep	0.00	0.00	0.00	0.00	-
Workshop - Social anxiety	0.00	0.00	0.00	0.00	-
Workshop - Panic attacks	0.02	0.31	0.00	0.00	0.1576
Assertiveness Group	0.19	2.30	0.09	1.63	0.5669
Bereavement Group	0.14	2.44	0.28	3.46	0.5601
M Phil Group	0.00	0.00	0.00	0.00	-
Managing Mood Group	0.14	2.44	0.00	0.00	0.3181
Perfectionism Group	0.00	0.00	0.12	2.04	0.3181
Post Graduate Group	0.00	0.00	0.00	0.00	-
Returners Group	0.02	0.23	0.01	0.16	0.5669
Self-esteem Group	0.12	2.04	0.12	2.04	0.9963
UG Group	0.23	4.07	0.00	0.00	0.3181

For peer review only - http://bmjopen.bmj.com/site/about/guidelines.xhtml

6.7 Resource use T0 to T3

Table 6.7: Aggregated UCS resource use by arm between T0 to T2. Means are the amount of each activity by student. SD= standard deviation

	MSS+SAU (n=309)		SAU (n=307)		
Resource use T0 to T3	n/Mean	SD	n/Mean	SD	P-value
Used UCS services: N=	64		73		
Individual assessment sessions	0.15	0.39	0.17	0.40	0.6607
Individual counselling sessions	0.52	1.76	0.72	2.68	0.2773
Total other individual activity	0.04	0.20	0.08	0.54	0.1375
Total workshops	0.07	0.34	0.05	0.26	0.3611
Total groups	0.03	0.18	0.03	0.16	0.6428

For peer review only

For peer review only - http://bmjopen.bmj.com/site/about/guidelines.xhtml

Table 6.8: Resource use across all participants, T0 to T3 (mean use per student)

	MSS+SAU (n=309)		SAU (n=307)		
Resource T0 to T3	n/Mean	SD	n/Mean	SD	P-value
Assessment	0.15	0.39	0.17	0.40	0.6607
Counselling	0.52	1.76	0.72	2.68	0.2773
Client Contact by Phone or Email	0.00	0.00	0.00	0.06	0.3181
MHA Assessment	0.01	0.10	0.02	0.14	0.3104
MHA Ongoing	0.00	0.06	0.04	0.47	0.1454
Pre Group	0.02	0.15	0.02	0.14	0.7887
Can't Work Group	0.01	0.11	0.01	0.08	0.4169
Returners - Anxiety	0.00	0.00	0.00	0.00	-
Returners - Self Compassion	0.00	0.00	0.00	0.00	-
Returners - Time Management	0.00	0.00	0.00	0.00	-
Workshop - Anxiety	0.01	0.10	0.01	0.08	0.6591
Workshop - CBT for self-help	0.00	0.06	0.00	0.06	0.9963
Workshop - Exam Preparation	0.00	0.06	0.00	0.06	0.9963
Workshop - Food and mood	0.01	0.11	0.00	0.00	0.0453
Workshop - Procrastination	0.01	0.11	0.02	0.13	0.7302
Workshop - Self-compassion	0.01	0.11	0.01	0.14	0.9934
Workshop - Sleep	0.00	0.00	0.00	0.00	-
Workshop - Social anxiety	0.00	0.00	0.00	0.00	-
Workshop - Panic attacks	0.01	0.08	0.00	0.06	0.5669
Assertiveness Group	0.01	0.10	0.01	0.08	0.6591
Bereavement Group	0.00	0.06	0.01	0.08	0.5601
M Phil Group	0.00	0.00	0.00	0.00	-
Managing Mood Group	0.00	0.06	0.00	0.00	0.3181
Perfectionism Group	0.00	0.00	0.00	0.06	0.3181
Post Graduate Group	0.00	0.06	0.00	0.06	0.9963
Returners Group	0.01	0.08	0.00	0.06	0.5669
Self-esteem Group	0.00	0.06	0.00	0.06	0.9963
UG Group	0.00	0.06	0.00	0.00	0.3181

For peer review only - http://bmjopen.bmj.com/site/about/guidelines.xhtml

Table 6.9: Resource use across participants *using* a particular service T0 to T3 (eg means here exclude participants not using that UCS offering)

	MSS+SAU (n=309)		SAU (n=307)	
	Used (N)	Used mean	Used (N)	Used mean
Assessment	43	1.09	48	1.06
Counselling	41	3.93	49	4.51
Client Contact by Phone or Email	0	-	1	1.00
MHA Assessment	3	1.00	6	1.00
MHA Ongoing	1	1.00	3	4.33
Pre Group	7	1.00	6	1.00
Can't Work Group	4	1.00	2	1.00
Returners - Anxiety	0	-	0	-
Returners - Self Compassion	0	-	0	-
Returners - Time Management	0	-	0	-
Workshop - Anxiety	3	1.00	2	1.00
Workshop - CBT for self-help	1	1.00	1	1.00
Workshop - Exam Preparation	1	1.00	1	1.00
Workshop - Food and mood	4	1.00	0	-
Workshop - Procrastination	4	1.00	5	1.00
Workshop - Self-compassion	4	1.00	3	1.33
Workshop - Sleep	0	-	0	-
Workshop - Social anxiety	0	-	0	-
Workshop - Panic attacks	2	1.00	1	1.00
Assertiveness Group	3	1.00	2	1.00
Bereavement Group	1	1.00	2	1.00
M Phil Group	0	-	0	-
Managing Mood Group	1	1.00	0	-
Perfectionism Group	0	-	1	1.00
Post Graduate Group	1	1.00	1	1.00
Returners Group	2	1.00	1	1.00
Self-esteem Group	1	1.00	1	1.00
UG Group	1	1.00	0	-

For peer review only - http://bmjopen.bmj.com/site/about/guidelines.xhtml

6.8 Costs T0 to T3

Table 6.10: Mean cost (per student) incurred by the UCS, T0 to T3

Costs T0 to T3	MSS+SAU (n=309)		SAU (n=307)		P-value
	n/Mean (£)	SD (£)	n/Mean (£)	SD (£)	
Individual assessment sessions	6.45	16.72	7.05	16.89	0.6607
Individual counselling sessions	14.37	48.65	19.85	73.82	0.2773
Total other individual activity	0.84	5.65	2.32	15.71	0.1201
Total workshops	0.36	1.66	0.24	1.22	0.2991
Total groups	1.25	8.48	1.04	7.59	0.7448
MSS course	52.82	0.00	0.00	0.00	
Total	76.07	63.27	30.49	84.97	0.0000

review only

For peer review only - http://bmjopen.bmj.com/site/about/guidelines.xhtml

Table 6.11: Mean costs per student across all participants, T0 to T3

UCS activity costs T0 to T3	MSS+SAU (n=309)		SAU (n=307)		P-value
	n/Mean (£)	SD (£)	n/Mean (£)	SD (£)	
Assessment	6.45	16.72	7.05	16.89	0.6607
Counselling	14.37	48.65	19.85	73.82	0.2773
Client Contact by Phone or Email	0.00	0.00	0.03	0.61	0.3181
MHA Assessment	0.41	4.17	0.83	5.88	0.3104
MHA Ongoing	0.09	1.57	1.17	12.85	0.1454
Pre Group	0.34	2.21	0.29	2.06	0.7887
Can't Work Group	0.12	1.08	0.06	0.77	0.4169
Returners - Anxiety	0.00	0.00	0.00	0.00	-
Returners - Self Compassion	0.00	0.00	0.00	0.00	-
Returners - Time Management	0.00	0.00	0.00	0.00	-
Workshop - Anxiety	0.04	0.37	0.02	0.31	0.6591
Workshop - CBT for self-help	0.01	0.22	0.01	0.22	0.9963
Workshop - Exam Preparation	0.01	0.22	0.01	0.22	0.9963
Workshop - Food and mood	0.05	0.43	0.00	0.00	0.0453
Workshop - Procrastination	0.05	0.43	0.06	0.48	0.7302
Workshop - Self-compassion	0.05	0.43	0.05	0.53	0.9934
Workshop - Sleep	0.00	0.00	0.00	0.00	-
Workshop - Social anxiety	0.00	0.00	0.00	0.00	-
Workshop - Panic attacks	0.02	0.31	0.01	0.22	0.5669
Assertiveness Group	0.28	2.81	0.19	2.31	0.6591
Bereavement Group	0.14	2.44	0.28	3.46	0.5601
M Phil Group	0.00	0.00	0.00	0.00	-
Managing Mood Group	0.14	2.44	0.00	0.00	0.3181
Perfectionism Group	0.00	0.00	0.12	2.04	0.3181
Post Graduate Group	0.32	5.70	0.33	5.72	0.9963
Returners Group	0.02	0.23	0.01	0.16	0.5669
Self-esteem Group	0.12	2.04	0.12	2.04	0.9963
UG Group	0.23	4.07	0.00	0.00	0.3181

For peer review only - http://bmjopen.bmj.com/site/about/guidelines.xhtml

6.9 CORE-6D utilities by arm and cohort

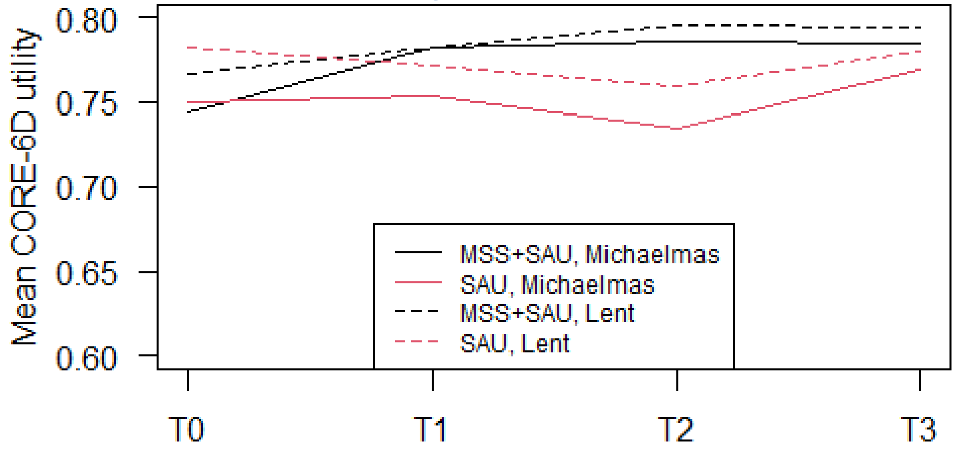

Figure 6.4: Mean (per student) CORE-6D utilities by arm and cohort (N within each group varies by time point – see Table 1).

For peer review only - http://bmjopen.bmj.com/site/about/guidelines.xhtml

6.10 Cost-effectiveness planes

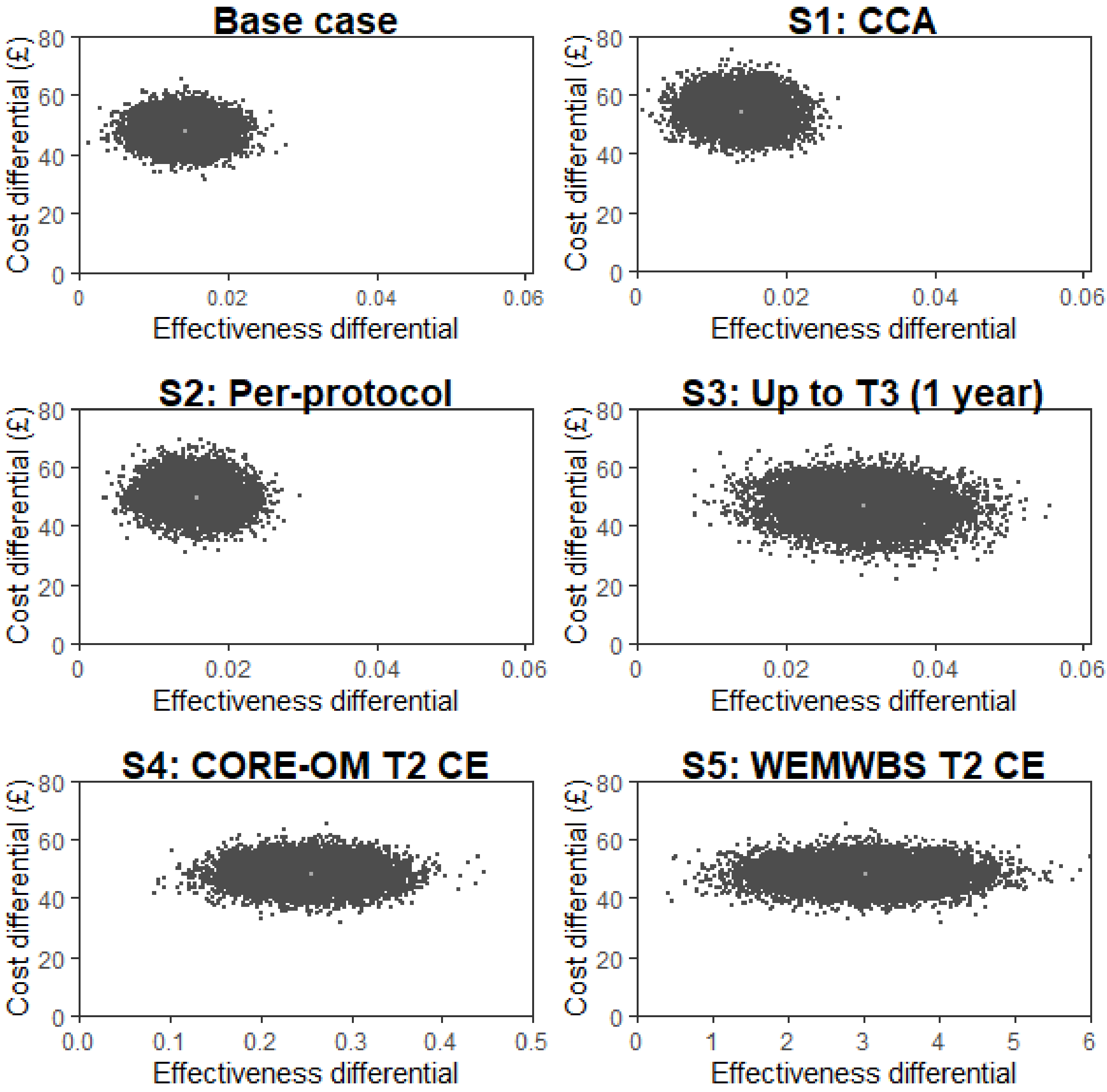

Figure 6.5: Cost-effectiveness planes resulting from the bootstrap re-sampling

For peer review only - http://bmjopen.bmj.com/site/about/guidelines.xhtml

Husereau D, Drummond M, Petrou S, Carswell C, Moher D, Greenberg D, et al. Consolidated Health Economic Evaluation Reporting Standards (CHEERS)--explanation and elaboration: a report of the ISPOR Health Economic Evaluation Publication Guidelines Good Reporting Practices Task Force. Value Health. 2013;16(2):231-50.

CHEERS checklist. Unless indicated otherwise, page numbers relate to the main text file.

Section/item	Item No	Recommendation	Reported on page/section
**Title and abstract**			
Title	1	Identify the study as an economic evaluation or use more specific terms such as "cost-effectiveness analysis", and describe the interventions compared.	p.1
Abstract	2	Provide a structured summary of objectives, perspective, setting, methods (including study design and inputs), results (including base case and uncertainty analyses), and conclusions.	p.2
**Introduction**			
Background and objectives	3	Provide an explicit statement of the broader context for the study.	p.3, Section 1
		Present the study question and its relevance for health policy or practice decisions.	p.3, Section 1
**Methods**			
Target population and subgroups	4	Describe characteristics of the base case population and subgroups analysed, including why they were chosen.	p.3, Section 2.1
Setting and location	5	State relevant aspects of the system(s) in which the decision(s) need(s) to be made.	p.3, Section 2.1
Study perspective	6	Describe the perspective of the study and relate this to the costs being evaluated.	p.4, Section 2.2
Comparators	7	Describe the interventions or strategies being compared and state why they were chosen.	p.3, Section 2.1
Time horizon	8	State the time horizon(s) over which costs and consequences are being evaluated and say why appropriate.	p.5-7, Section 2.4
Discount rate	9	Report the choice of discount rate(s) used for costs and outcomes and say why appropriate.	Noted not applicable, p.7, Section 2.4
Choice of health outcomes	10	Describe what outcomes were used as the measure(s) of benefit in the evaluation	p.5, Section 2.3

For peer review only - http://bmjopen.bmj.com/site/about/guidelines.xhtml

		and their relevance for the type of analysis performed.	
Measurement of effectiveness	11a	*Single study-based estimates:* Describe fully the design features of the single effectiveness study and why the single study was a sufficient source of clinical effectiveness data.	p.4, Section 2.3
	11b	*Synthesis-based estimates:* Describe fully the methods used for identification of included studies and synthesis of clinical effectiveness data.	Not applicable
Measurement and valuation of preference based outcomes	12	If applicable, describe the population and methods used to elicit preferences for outcomes.	(Not elicited in this study, but relevant details p.5, Section 2.3)
Estimating resources and costs	13a	*Single study-based economic evaluation:* Describe approaches used to estimate resource use associated with the alternative interventions. Describe primary or secondary research methods for valuing each resource item in terms of its unit cost. Describe any adjustments made to approximate to opportunity costs.	p.4, Section 2.2, p.8, Section 3.3, Table 2-4<br><br>Supplementary materials
	13b	*Model-based economic evaluation:* Describe approaches and data sources used to estimate resource use associated with model health states. Describe primary or secondary research methods for valuing each resource item in terms of its unit cost. Describe any adjustments made to approximate to opportunity costs.	Not applicable
Currency, price date, and conversion	14	Report the dates of the estimated resource quantities and unit costs. Describe methods for adjusting estimated unit costs to the year of reported costs if necessary. Describe methods for converting costs into a common currency base and the exchange rate.	As applicable, p.4, Section 2.2
Choice of model	15	Describe and give reasons for the specific type of decision-analytical model used. Providing a figure to show model structure is strongly recommended.	Not applicable
Assumptions	16	Describe all structural or other assumptions underpinning the decision-analytical model.	Not applicable
Analytical methods	17	Describe all analytical methods supporting the evaluation. This could include methods for dealing with skewed, missing, or censored data; extrapolation methods; methods for pooling data;	p.5-7, Section 2.4<br><br>Supplementary materials

For peer review only - http://bmjopen.bmj.com/site/about/guidelines.xhtml

		approaches to validate or make adjustments (such as half cycle corrections) to a model; and methods for handling population heterogeneity and uncertainty.	
**Results**			
Study parameters	18	Report the values, ranges, references, and, if used, probability distributions for all parameters. Report reasons or sources for distributions used to represent uncertainty where appropriate. Providing a table to show the input values is strongly recommended.	Not applicable
Incremental costs and outcomes	19	For each intervention, report mean values for the main categories of estimated costs and outcomes of interest, as well as mean differences between the comparator groups. If applicable, report incremental cost-effectiveness ratios.	p.8-11, Sections 3.2-3.5 Tables 2-5<br><br>Supplementary materials
Characterising uncertainty	20a	*Single study-based economic evaluation:* Describe the effects of sampling uncertainty for the estimated incremental cost and incremental effectiveness parameters, together with the impact of methodological assumptions (such as discount rate, study perspective).	p.11, Section 3.6 Figure 2<br><br>Supplementary materials
	20b	*Model-based economic evaluation:* Describe the effects on the results of uncertainty for all input parameters, and uncertainty related to the structure of the model and assumptions.	Not applicable
Characterising heterogeneity	21	If applicable, report differences in costs, outcomes, or cost-effectiveness that can be explained by variations between subgroups of patients with different baseline characteristics or other observed variability in effects that are not reducible by more information.	Not applicable
**Discussion**			
Study findings, limitations, generalisability, and current knowledge	22	Summarise key study findings and describe how they support the conclusions reached. Discuss limitations and the generalisability of the findings and how the findings fit with current knowledge.	p.13-15 Sections 4-5
**Other**			
Source of funding	23	Describe how the study was funded and the role of the funder in the identification, design, conduct, and reporting of the	p.15

For peer review only - http://bmjopen.bmj.com/site/about/guidelines.xhtml

		analysis. Describe other non-monetary sources of support.	
Conflicts of interest	24	Describe any potential for conflict of interest of study contributors in accordance with journal policy. In the absence of a journal policy, we recommend authors comply with International Committee of Medical Journal Editors recommendations.	p.15

For peer review only

For peer review only - http://bmjopen.bmj.com/site/about/guidelines.xhtml

BMJ Open

Cost-effectiveness of providing university students with a mindfulness-based intervention to reduce psychological distress: economic evaluation of a pragmatic randomised controlled trial

Journal:	*BMJ Open*
Manuscript ID	bmjopen-2023-071724.R1
Article Type:	Original research
Date Submitted by the Author:	18-Sep-2023
Complete List of Authors:	Wagner, Adam; NIHR Applied Research Collaboration (ARC) East of England (EoE), ; University of East Anglia, Norwich Medical School<br>Galante, Julieta; University of Cambridge, Department of Psychiatry; University of Melbourne, Contemplative Studies Centre, Melbourne School of Psychological Sciences, Faculty of Medicine, Dentistry, and Health Sciences<br>Dufour, Géraldine; Therapeutic Consultations Ltd ; European Association for International Education<br>Barton, Garry; University of East Anglia, Norwich Medical School<br>Stochl, Jan; University of Cambridge, Department of Psychiatry; Charles University, Department of Kinanthropology<br>Vainre, Maris; Cambridge University, MRC Cognition and Brain Sciences Unit<br>Jones, Peter; University of Cambridge, Department of Psychiatry; NIHR Applied Research Collaboration (ARC) East of England (EoE)
**Primary Subject Heading**:	Health economics
Secondary Subject Heading:	Mental health
Keywords:	HEALTH ECONOMICS, MENTAL HEALTH, PSYCHIATRY

SCHOLARONE™ Manuscripts

I, the Submitting Author has the right to grant and does grant on behalf of all authors of the Work (as defined in the below author licence), an exclusive licence and/or a non-exclusive licence for contributions from authors who are: i) UK Crown employees; ii) where BMJ has agreed a CC-BY licence shall apply, and/or iii) in accordance with the terms applicable for US Federal Government officers or employees acting as part of their official duties; on a worldwide, perpetual, irrevocable, royalty-free basis to BMJ Publishing Group Ltd ("BMJ") its licensees and where the relevant Journal is co-owned by BMJ to the co-owners of the Journal, to publish the Work in this journal and any other BMJ products and to exploit all rights, as set out in our licence.

The Submitting Author accepts and understands that any supply made under these terms is made by BMJ to the Submitting Author unless you are acting as an employee on behalf of your employer or a postgraduate student of an affiliated institution which is paying any applicable article publishing charge ("APC") for Open Access articles. Where the Submitting Author wishes to make the Work available on an Open Access basis (and intends to pay the relevant APC), the terms of reuse of such Open Access shall be governed by a Creative Commons licence – details of these licences and which Creative Commons licence will apply to this Work are set out in our licence referred to above.

Other than as permitted in any relevant BMJ Author's Self Archiving Policies, I confirm this Work has not been accepted for publication elsewhere, is not being considered for publication elsewhere and does not duplicate material already published. I confirm all authors consent to publication of this Work and authorise the granting of this licence.

For peer review only

Cost-effectiveness of providing university students with a mindfulness-based intervention to reduce psychological distress: economic evaluation of a pragmatic randomised controlled trial

Adam P Wagner, Julieta Galante, Géraldine Dufour, Garry Barton, Jan Stochl, Maris Vainre, and Peter B Jones

12/09/2023

Adam P Wagner, PhD, (Corresponding author) Senior Research Fellow, Norwich Medical School, University of East Anglia, Norwich Research Park, Norwich, NR4 7TJ, United Kingdom (second affiliation: National Institute for Health Research (NIHR) Applied Research Collaboration (ARC) East of England (EoE)). adam.wagner@uea.ac.uk

Julieta Galante, PhD, Honorary Senior Visiting Research Fellow, Department of Psychiatry, University of Cambridge, Douglas House, 18b Trumpington Road, Cambridge, CB2 8AH, United Kingdom (second affiliation: Contemplative Studies Centre, Melbourne School of Psychological Sciences, Faculty of Medicine, Dentistry, and Health Sciences, University of Melbourne). mjg231@cam.ac.uk phone (+44) 7986 172834

Géraldine Dufour, MA, Geraldine Dufour Therapeutic Consultations Ltd https://www.geraldinedufour.com/ (second affiliation: Associate Executive Member, European Association for International Education (EAIE)).

Garry Barton, PhD, Professor of Health Economics, Norwich Medical School, University of East Anglia, Norwich Research Park, Norwich, NR4 7TJ, United Kingdom.

Jan Stochl, PhD, Senior Research Associate, Department of Psychiatry, University of Cambridge, Cambridge Biomedical Campus, Box 189, Cambridge, CB2 0QQ, United Kingdom (second affiliation: Department of Kinanthropology, Charles University, Jose Martiho 31, 162 52 Prague, Czech Republic; third affiliation: NIHR ARC EoE).

Maris Vainre, MA, PhD Student, MRC Cognition and Brain Sciences Unit, University of Cambridge, 15 Chaucer Rd, Cambridge, CB2 7EF, United Kingdom (former affiliation when doing this work: NIHR ARC EoE). Maris.Vainre@mrc-cbu.cam.ac.uk

Peter B Jones, PhD, Professor of Psychiatry, Department of Psychiatry, University of Cambridge, Herchel Smith Building, Cambridge, CB2 0SZ, United Kingdom (second affiliation: NIHR ARC EoE).

Word count: 4,742

For peer review only

Abstract

Objective: Increasing numbers of young people attending university has raised concerns about the capacity of student mental health services to support them. We conducted a randomised controlled trial (RCT) to explore whether provision of an 8-week mindfulness course adapted for university students (Mindfulness Skills for Students – MSS), compared to university mental health support as usual (SAU), reduced psychological distress during the exam period. Here, we conduct an economic evaluation of MSS+SAU compared to SAU.

Design and setting: Economic evaluation conducted alongside a pragmatic, parallel, single-blinded randomised controlled trial (RCT) comparing provision of MSS+SAU to SAU.

Participants: 616 university students randomised.

Primary and secondary outcome measures: The primary economic evaluation assessed the cost per quality-adjusted life year (QALY) gained from the perspective of the university counselling service. Costs relate to staff time required to deliver counselling service offerings. QALYs were derived from the CORE-6D preference based tool, which uses responses to six items of the Clinical Outcomes in Routine Evaluation Outcome Measure (CORE-OM; primary clinical outcome measure). Primary follow-up duration was 5 and 7 months for the two recruitment cohorts.

Results: It was estimated to cost £1584.48 (2022 prices) to deliver an MSS course to 30 students, £52.82 per student. Both costs(adjusted mean difference: £48.04, 95%CI £39.92-£56.16) and QALYs (adjusted mean difference: 0.014, 95%CI 0.008-0.021) were significantly higher in the MSS arm compared to SAU. The incremental cost-effectiveness ratio (ICER) was £3,355, with a very high (99.99%) probability of being cost-effective at a willingness-to-pay threshold of £20,000 per QALY.

Conclusions: MSS leads to significantly improved outcomes at a moderate additional cost. The ICER of £3,355 per QALY suggests that MSS is cost-effective when compared to the UK's National Institute for Health and Care Excellence (NICE) thresholds of £20K per QALY.

Trial registration: Australian and New Zealand Clinical Trials Registry, ACTRN12615001160527

300/300 words

Strengths and limitations of this study

- Contrasting with many evaluations of mindfulness, a key strength of this study/economic evaluation is that it drew on data from a large ($n > 600$) RCT that followed-up participants for up to 12 months.
- A key limitation is the narrow costing perspective adopted – the university counselling service – that does not capture all costs of mental health support and will miss wider impacts.
- Additionally, we have focused exclusively on wage costs (including on-costs) – we have not considered wider overheads or other resources (such as room hire), subsequently underestimating costs.

For peer review only - http://bmjopen.bmj.com/site/about/guidelines.xhtml

For peer review only

1 Background

Depression is among the top causes of morbidity, generating a huge burden on populations all over the world [1]. This trend is also seem in youth, with the prevalence of a probable mental health disorder among those aged 17–19 in England reaching almost one in four in 2022 [2]. University counselling services in the UK have seen a rising trend in the proportion of students asking for mental health support [3]; indeed, it has been noted that the increase in student numbers seeking counselling is greater than the growth of overall student numbers [4]. Similar issues have been noted in the university sector beyond England [5,6]. Thus, an effective preventative intervention is needed to address this growing demand and need [7].

A widely used working definition of mindfulness is `the awareness that emerges through paying attention on purpose, in the present moment, and non-judgmentally to the unfolding of experience moment by moment' [8]. It is popular among students [9], and some of the popularity may be because it seeks to teach skills, rather than directly addressing mental health issues – it is non-stigmatising [10].

Given the growing demand for counselling services and limited university funding, it is important to have evidence for the effectiveness and cost implications of associated interventions [11]. Thus, when the University of Cambridge's (UoC's) University Counselling Service (UCS) developed the 'Mindfulness Skills for Students' (MSS) programme, they evaluated it in the Mindful Student Study: a large randomised controlled trial (RCT) [12]. It has been demonstrated that, compared to support as usual (SAU), MSS + SAU reduces students' psychological distress during the academic examination period, three to six months after randomisation [13]. MSS participants were followed up for 12 months post randomisation: compared to support as usual (SAU), MSS + SAU continued to have small but significant reductions in distress at this time [14]. This result suggests that the MSS programme, via reducing psychological distress, could prevent future cases of mental ill-health [15]. Here, we conduct an economic evaluation of MSS to explore its costs.

2 Method

2.1 Participants

The Mindful Student Study was a pragmatic randomised trial at the / UoC in the United Kingdom (UK).

The trial inclusion criteria were: (a) undergraduate or postgraduate UoC students; (b) who were interested in attending at least seven sessions of the course. The exclusion criteria were: (a) severe anxiety or depression at that time; (b) severe mental illness such as psychosis; (c) recent bereavement; and (d) any other serious health problem that would affect their ability to engage with the course. The selection criteria were assessed by the students themselves.

For peer review only - http://bmjopen.bmj.com/site/about/guidelines.xhtml

The study was advertised widely to the student community, using physical (posters), social media (eg Facebook and Twitter) and information sessions – see [12] for further detail. Where students agreed to take part, they were emailed a personal link to an online baseline questionnaire. Upon completing this questionnaire, participants underwent 1:1 randomisation to receive either: an eight week mindfulness course adapted for university students plus support as usual (MSS+SAU); or SAU alone. SAU consisted of the possibility of accessing, if the student desired, comprehensive UCS support in addition to other health support available from the UoC and its colleges, and from health services including the National Health Service, external to the UoC. Participants randomised to SAU were guaranteed a space in the following year's mindfulness courses and were requested to inform the team if they decided to learn mindfulness elsewhere during the follow-up period. Further detail on the trial can be found in [12] and [13].

Students were recruited in two cohorts: at the beginning of the autumn term (Michaelmas) term and at the beginning of the spring (Lent) term. Both cohorts were followed-up at: point of recruitment (T0); after the delivery of the mindfulness courses (SAU having *not* received the course) (T1); during the exam term (T2); and 12 months after recruitment (T3). The date of exam term follow-up, T2, was common across cohorts, but the date of other follow-up points differed by cohort: the relationships are depicted in Figure 1. Thus, the follow-up duration at T2 was approximately 7 months for the Michaelmas cohort and 5 months for the later recruited Lent cohort.

This study was approved by the Cambridge Psychology Research Ethics committee on August 25, 2015 (reference: PRE.2015.060). The study is registered with the Australia and New Zealand Clinical Trials Registry (ACTRN12615001160527). The Health Economic Analysis Plan (HEAP) that this economic evaluation follows was pre-specified and is available online [16].

2.2 Costs

Costs were estimated from the perspective of the UoC's operating budget (primarily covering salaries of staff; excludes room hire) - hereafter referred to as the `UCS perspective'. Use of SAU UCS offerings were extracted from the UCS record system (consequently, there is no missing data on UCS service use) following participant agreements and strict confidentiality of access protocols.

At the time of this evaluation, routine UCS services were categorised into three broad groups: 'individual' sessions providing support to one student (includes assessment sessions); 'workshops' - one-off offerings that more than one student attends; and 'groups' - a number of sessions attended by multiple students (where a student stops attending a group, their place cannot be used by another student). In consultation with the then UCS head (GD), who consulted the UCS senior management team, we determined the usual staff grade delivering each offering and the associated typical time taken to prepare, deliver and record (e.g. completing notes etc) each of these session types. To calculate the cost to the UCS of a given offering, we multiplied the time taken to prepare, deliver and record it by the hourly rate of employing staff at the required grade. For workshops and groups, the cost per student (unit cost) of an offering is determined by dividing its total cost by the

For peer review only - http://bmjopen.bmj.com/site/about/guidelines.xhtml

number of students typically attending each offering (details given in the Supplementary file).

The total cost of delivering a course of mindfulness (the intervention) to 30 students (maximum capacity of a course) was determined through consultation with the then UCS head (GD) and the course teacher. Accordingly, the activities involved in delivering a course were identified, along with the total time, and grade of staff, delivering each activity. Total times were multiplied by appropriate hourly rates of staff delivering each course activity and summed to give the total cost of delivering a Mindfulness course. The cost per student (unit cost) of delivering the course was determined by dividing this total cost by the capacity of each class ($n = 30$ students). This cost was ascribed to each of the students in the MSS+SAU arm (multiple courses were being delivered in parallel for each cohort of MSS+SAU). This cost was irrespective of student attendance: the nature of the mindfulness course is such that a student cannot join part way through, so a non-attending student cannot be `replaced'; this is in line with other UCS groups.

Hourly cost of employing staff were taken from the UoC Human Resources website and include on-costs (such as pension and national insurance contribution – see Supplementary file) but no further overheads. They were the rates as applied from March 2022, and so can be considered to apply to the 2022/2023 financial year. Costs are reported in pound sterling.

2.3 Outcomes

The primary outcome measure was the participant completed Clinical Outcomes in Routine Evaluation Outcome Measure (CORE-OM), a 34-item generic questionnaire designed to evaluate efficacy and effectiveness across multiple disciplines delivering psychological therapies [17]. The CORE-OM has been found to have strong convergent validity, internal and test-retest reliability and sensitivity to change [18], and has been extensively used with UK university students [19]. Each CORE-OM item is scored on a five-point scale ranging from 0 (not at all) to 4 (most or all the time). The corresponding sum-total score ranges 0–136; this is usually divided by the number of completed items to form a total mean score. A higher mean-score indicates more distress. CORE-OM was collected at each of the data collection points (T0-T3); a comparison of CORE-OM between arms during the exam term (T2) was the primary (clinical effectiveness) outcome [12,13].

The CORE-6D is a preference-based tool to derive mental health specific health state utility, based on a subset of six questions/dimensions from the CORE-OM [20]: using time trade-off methods, [21] a sample of the general public were surveyed for their preferences for different health states as described by the CORE-6D [22]. Scores generated from the survey are converted to an index score, that can be used as a valuation of all health states represented by the CORE-6D: details of the steps for these calculations are provided in an Appendix to the HEAP [16]. Here, utility scores were calculated for T0-T3 using this valuation. In line with the primary clinical-effectiveness outcome, the base-case (primary) economic analysis uses quality adjusted life years (QALYs) from T0 to T2 as outcome; these are calculated via the area under the curve approach [23] with linear interpolation between points. Given different periods between cohorts in which to accrue QALYs (see

For peer review only - http://bmjopen.bmj.com/site/about/guidelines.xhtml

Figure 1: Michaelmas cohort – 227 days; Lent – 147 days), QALYs are reported separately for each cohort (and subsequent regressions adjust for cohort). Additionally, we also calculated QALYs from T0 to T3.

The Warwick-Edinburgh Mental Wellbeing Scale (WEMWBS) was used at each time point to measure a broad conception of subjective well-being [24]. The WEMWBS has 14 items, each scored from one ('none of the time') to five ('all of the time'). A higher total score (ranging from 14-70) indicates higher wellbeing. The WEMWBS has good validity, internal consistency and test-retest reliability in samples of both UK students and general population [25].

2.4 Analyses

To reduce the risk of bias [26] and improve precision [27], we sought to address missing data, rather than conducting a complete case analysis (CCA). Following recommendations for within trial analyses of cost-effectiveness, patterns of missing data were explored to understand the potential missing data mechanisms [27]. Resource use information was complete as it came from internal UCS records. Where missingness occurred on the outcome scales, it nearly always meant that *no* items had been completed (see Supplementary file). Thus, we focused on summary measures of the outcomes (e.g. utilities, CORE-OM mean-score and WEMWBS total) in further missing data considerations. Details of missingness by outcome at each time point, overall and by arm, are given in Section 6.1: missingness increased by time point, and was between 5-10% higher in the SAU at T1 and T2, but were similar at T3. As advised in [27], we explored associations between missingness and both i) baseline variables (see Section 7.2) and observed outcomes (see Section 7.2.1). The associations indicated that the data was not missing completely at random (MCAR) – meaning that CCA would not be appropriate. In line with the analysis devised in [12] and conducted in [13], we assumed the data was missing at random (MAR), and used multiple-imputation (MI) to create fifty datasets (following the rule-of-thumb from [28] that the number of data-sets should equal the percentage of missing data). Note that the level of missing data at T3 ($\approx 45\%$) is notably higher than at T2 ($\approx 27\%$), with the latter time point being the most crucial for the base case which draws data from T0-T2 (the impact of imputing missing data is explored in the sensitivity analyses - see below).

The *R* package *mice* [29] was used to conduct MI, separately for each arm (as advised in [27]) using the significant ($p < 0.1$) predictors of missingness and those variables included in the final regression models (as advised in [28]): see Supplementary file for details. The separate data-sets were combined using Rubin's rules [30].

Costs and outcomes were analysed simultaneously with bivariate regression models, allowing for correlations between costs and outcomes to be incorporated (in contrast to separate regressions). Such regressions are generally robust to skewed data [31]. Data was analysed using an intention-to-treat (ITT) approach: participants were analysed in the group to which they were randomised. All regressions (both cost and outcome) included covariates for: arm; sex; and cohort. Outcome regressions additionally included the baseline measure of the corresponding outcome (or utility, when analysing QALYs).

For peer review only - http://bmjopen.bmj.com/site/about/guidelines.xhtml

The estimated coefficient of arm in these regressions allowed the incremental cost-effectiveness ratio (ICER) to be estimated [32]: in this context, this corresponded to the mean incremental cost divided by the mean incremental effect (for the QALYs, COME-OM and WEMWBS outcomes) for MSS + SAU compared to SAU. However, were either alternative – MSS + SAU or SAU – both less costly and more effective, that alternative would be categorised as 'dominant' and the ICER would not be calculated. Otherwise, the ICER can be used to measure whether the extra cost associated with MSS + SAU is considered to constitute value for money. In other contexts, such as economic evaluations of new interventions for use in the United Kingdom's (UK's) National Health Service (NHS), the National Institute for Health and Care Excellence (NICE; [33]) uses a cost-effectiveness threshold value (λ) of £20,000-30,000 per QALY [34]. There is no agreed threshold for the perspective of costs to the UCS.

Uncertainty in the mean incremental costs and mean incremental effect (for each outcome), which determines estimated levels of cost-effectiveness, were explored using bootstrap re-sampling [35]: 200 replications were taken from each of the 50 imputations (in line with [27]), with re-sampling stratified by sex and cohort. From the replicates, cost-effectiveness planes were constructed for the base case and each scenario; each plane (one per outcome) displays the estimated mean incremental cost and mean incremental effect for each replicate [36], with location and spread of points showing uncertainty. Additionally, cost-effectiveness acceptability curves (CEACs) were constructed for the base case and each scenario, and show the probability of MSS + SAU being cost-effective compared to SAU at a range of willingness-to-pay thresholds [37].

In the base-case (primary) economic analysis, the QALYs and costs up to T2 – in line with the primary clinical-effectiveness outcome in [13] – are considered. To check the robustness of the corresponding conclusions, a number of sensitivity analyses [32] were conducted in which the assumptions of the base-case analysis were varied:

1. CCA – repeats the base case, but only including participants for whom we have complete data; investigates the impact of MI;
2. Per-protocol analysis – investigates how results differ if we only consider those attending at least 50% (four out of eight) of the mindfulness sessions;
3. Conduct evaluation at 12 months – extend the base-case analysis to include costs and benefits up to T3, investigating whether benefits continue beyond the examination term;
4. Conduct a cost-effectiveness analysis (CEA) for CORE-OM – as for the base-case, but measure benefits in terms of CORE-OM at T2 (e.g., calculate the cost per CORE-OM point);
5. Conduct a CEA for WEMWBS – as for the base-case, but measure benefits in terms of WEMWBS at T2 (e.g., calculate the cost per WEMWBS point).

All analysis were conducted in *R* [38]. No costs or benefits were considered beyond 12 months, so no discounting was undertaken [32,34].

For peer review only

2.5 Patient and Public Involvement statement

Study plans were reviewed by a group comprising representatives from the UCS, the university Academic Division, student representatives and college tutors. A focus group with students who had completed mindfulness courses taught before the trial was conducted to consult about study plans prior to submission for ethics approval.

Subsequently, an advisory reference group was put together comprising student representatives, members of the UCS and other student welfare staff. This group met approximately three times per year during the trial. Study researchers attended these meetings and presented updates. The group also advised on appropriate dissemination strategies.

3 Results

3.1 Participants

A total of 616 students were recruited to the trial during the academic year 2016/2017 (see Figure 1): 342 were recruited during September 2016 (Michaelmas cohort), 172 to MSS + SAU and 170 to SAU; 274 were during January 2017 (Lent cohort), 137 students to each arm. A CONSORT diagram of participants is given in Section 6.3. Both arms (see Table 1) had an approximate equal age of 23 years, but MSS + SAU had proportionately fewer females: 61% versus 65%, a non-significant difference. Other variables (including: ethnic origin; disability; degree level; year of study and department) were well balanced across arms of the trial (see [13]).

At baseline, participants reported more psychological distress than the general UK student population (mean (total mean score)=0.76, SD=0.59) [17], but lower levels that those who attend university counselling services in the UK (total mean score =1.85, SD=0.51) [39]. Our sample's mean score is just below the CORE-OM's recommended clinical cut-off score of 1 point, selected as a threshold to discriminate optimally between a clinical sample and a general population sample [40]. Participants also reported lower wellbeing scores than the general student population [24].

For peer review only

Table 1: Univariate comparisons of variables and outcomes by arm. Utilities and QALYs separated by cohort given differing durations to accrue QALYs.

		MSS+SAU (n=309)			SAU (n=307)			
		n	n/Mean	SD	n	n/Mean	SD	P-value
	Gender female (n)	309	187		307	201		0.2115
	Age	306	23	5	306	23	6	0.9219
Michaelmas cohort (172, 170)	Utility T0	172	0.744	0.114	168	0.750	0.121	0.6656
	Utility T1	138	0.782	0.093	135	0.754	0.115	0.0257
	Utility T2	126	0.786	0.098	122	0.735	0.127	0.0005
	Utility T3	92	0.784	0.100	103	0.769	0.122	0.3244
	QALYs T0-T2	116	0.483	0.045	109	0.464	0.058	0.0064
	QALYs T0-T3	86	0.778	0.071	88	0.749	0.094	0.0237
Lent cohort (137, 137)	Utility T0	137	0.767	0.114	136	0.782	0.104	0.2415
	Utility T1	115	0.783	0.101	90	0.771	0.107	0.4313
	Utility T2	109	0.796	0.111	94	0.759	0.112	0.0205
	Utility T3	77	0.794	0.092	66	0.779	0.120	0.4044
	QALYs T0-T2	102	0.316	0.030	85	0.312	0.032	0.3513
	QALYs T0-T3	70	0.853	0.066	60	0.828	0.081	0.0595
CORE-OM mean score	T0	309	1.0	0.5	305	1.0	0.5	0.4102
	T1	255	0.9	0.5	227	1.0	0.5	0.0022
	T2	237	0.9	0.5	216	1.1	0.6	0.0000
	T3	169	0.8	0.5	169	0.9	0.6	0.0312
WEMWBS sum score	T0	307	48.0	8.6	307	48.6	8.5	0.3900
	T1	254	49.6	8.9	221	46.9	9.0	0.0009
	T2	235	48.9	9.0	214	46.4	9.1	0.0031
	T3	168	51.1	9.6	167	48.8	8.9	0.0243

For peer review only - http://bmjopen.bmj.com/site/about/guidelines.xhtml

3.2 Resource use

The UCS has a wide range of offerings: use of the most common individual offerings (individual assessment and individual counselling sessions) and aggregates across the different types of UCS offerings between T0 and T2 are compared by arm in Table 2. Relatively small proportions in each arm used routine UCS offerings: 16% in MSS + SAU and 18% in SAU, a non-significant difference ($p = 0.5199$). Compared to SAU, the MSS + SAU arm used on average fewer routine UCS offerings, but none of the differences were significant ($p > 0.18$). More detailed resource use comparisons between arms are given in the Supplementary file.

Table 2: Aggregated UCS resource use by arm between T0 to T2. Means are the amount of each activity by student. SD= standard deviation

Resource use T0 to T2	MSS+SAU (n=309)		SAU (n=307)		P-value
	n/Mean	SD	n/Mean	SD	
Used UCS services: N=	49		55		
Individual assessment sessions	0.09	0.30	0.11	0.33	0.5937
Individual counselling sessions	0.30	1.31	0.47	1.70	0.1875
Total other individual activity	0.03	0.18	0.05	0.41	0.3637
Total workshops	0.07	0.33	0.04	0.25	0.2748
Total groups	0.03	0.16	0.02	0.14	0.5978

3.3 Unit costs

Hourly costs (including employer on-costs) of employing grade 4 and 7 staff respectively are (see Supplementary file for detailed information): £17.71 and £25.45. Grade 4 staff were administrators and support staff; grade 7 staff corresponded to UCS counsellors, or the mindfulness teacher when in relation to MSS. Unit costs for all UCS offerings are given in Section 6.5. The two most commonly delivered UCS activities are individual assessment and counselling sessions, both delivered by grade 7 staff, with estimated unit costs per student respectively of: £42.42 and £27.57. The resources and costs involved in delivering a course (for 30 students) of mindfulness are given in Table 3; the resulting cost (£52.82) per student is included as a cost for all MSS+SAU students.

For peer review only - http://bmjopen.bmj.com/site/about/guidelines.xhtml

Table 3: Resources and costs used to deliver a course of mindfulness, with capacity for 30 students.

Activities	Times per course	Duration	Staff grade	Cost (£)
Administrative support	1	23.25 hours	4	407
Mindfulness teacher project management	1	20.8 hours	7	509
Venue preparation	8	60 mins	7	204
Pre-session individual student contact time	8	15 mins	7	51
Group session - 1st	1	90 mins	7	38
Group session - 2nd-8th	7	75 mins	7	223
Post-session individual student contact time	8	15 mins	7	51
Post-session note recording	8	15 mins	7	51
Venue `tidy-up'	8	15 mins	7	51
Total				1,584
Total by student (n=30)				52.82

3.4 Costs

Aggregate costings per student for UCS and mindfulness course use from T0 to T2 by arm are given in Table 4; detailed costing by each activity are given in the Supplementary file. Excluding costs of the mindfulness course, costs are lower in the MSS + SAU arm, but not significantly so ($p > 0.18$). Overall, mean costs in the MSS + SAU arm (£66.91) are significantly ($p < 0.0001$) higher than in the SAU (£19.42), and this is primarily driven by the costs of the mindfulness course.

For peer review only - http://bmjopen.bmj.com/site/about/guidelines.xhtml

Table 4: Mean cost (per student) incurred by the UCS. SD= standard deviation

Costs T0 to T2	MSS+SAU (n=309)		SAU (n=307)		P-value
	n/Mean (£)	SD (£)	n/Mean (£)	SD (£)	
Individual assessment sessions	3.98	12.85	4.56	14.02	0.5937
Individual counselling sessions	8.39	36.15	12.84	46.92	0.1875
Total other individual activity	0.60	5.00	1.24	11.33	0.3712
Total workshops	0.30	1.49	0.16	0.94	0.1781
Total groups	0.83	6.12	0.62	4.77	0.6288
MSS course	52.82	0.00	0.00	0.00	
Total	66.91	46.28	19.42	56.35	0.0000

3.5 Outcomes

Unadjusted outcome comparisons are reported in Table 1.

Given the different periods each cohort has to accrue QALYs, we consider utilities and QALYs separately by cohort. Utilities are plotted by arm and cohort in the Supplementary file (within each cohort/arm group, N varies by time point – see Table 1). Irrespective of cohort, utilities for MSS + SAU (black) are higher (better) at T1-T3 than SAU (red); they are significantly higher at T2 (the primary outcome time-point) for both cohorts ($p < 0.02$). Compared to SAU, MSS + SAU accrued more QALYs by both T2 and T3, but the means were significantly ($p = 0.01$ and $p = 0.02$ respectively) higher in the Michaelmas cohort; in the Lent cohort, MSS+SAU arm approached significantly ($p = 0.06$) higher QALYs at T3.

At baseline (T0), CORE-OM mean scores were similar between arms, but for all subsequent points (T1-T3), MSS + SAU had significantly ($p < 0.03$) lower (better) mean scores than SAU. Mean WEMWBS sum-scores were similar at baseline (T0), but MSS + SAU scores were all significantly ($p < 0.02$) better (higher) than SAU (T1-T3).

3.6 Cost-effectiveness analysis

Estimates of the adjusted mean incremental costs and mean incremental outcomes (QALYs, CORE-OM improvement or WEMWBS improvement) generated from the bivariate regressions are shown in Table 5, alongside the corresponding ICERs (note that CORE-OM scores have been reversed, so direction of improvement aligns with other outcomes). CEACs for the base-case and scenarios S1-5 are shown in Figure 2.

In the base-case and all scenarios, MSS+SAU achieves significantly (95% CIs exclude zero) higher (better) outcomes, but is also significantly more expensive, than SAU. In this setting,

For peer review only - http://bmjopen.bmj.com/site/about/guidelines.xhtml

there are no established thresholds for what ICERs would be considered cost-effective. However, at NICE threshold of £20,000 per QALY, used in economic evaluations for the NHS, MSS+SAU would be considered cost-effective for the base-case and scenarios S1-3 (where outcomes are measured in terms of QALYs).

The uncertainty surrounding cost-effectiveness conclusions are depicted in the cost-effectiveness planes in the Supplementary file. All bootstrap replicates fell in the north-east quadrant for the base case and all scenarios S1-5: this means that when evaluating uncertainty using bootstrap methods, *all* bootstrap replicates indicated the MSS+SAU was both more costly and more effective than SAU.

The probability of cost-effectiveness, illustrated by the CEACs (Figure 2), rapidly moves to near certainty as the willingness-to-pay thresholds exceeds £6,000 per outcome unit (base case and S1-S3: QALYs; S4: CORE-OM unit; S5: WEMWBS unit). Considering the base case and scenarios S1-S3 at the NICE thresholds noted above, there is near certainty of MSS+SAU being more cost-effective than SAU: there is very little probability of making the wrong decision about cost-effectiveness at this threshold.

Table 5: UCS costs (£), outcomes (base case and S1-S3: QALYs; S4: CORE-OM unit; S5: WEMWBS unit) and ICER for base-case and scenarios, where SAU is the reference. Costs and outcomes adjusted for sex and cohort; additionally, outcomes adjusted by corresponding baseline outcome measurement. CORE-OM scores reversed to align direction of improvement with other outcomes: an increase in CORE-OM here indicates an improvement (reduction) on the original scale. Incre. dif. = incremental difference. CI = confidence interval.

Scenario	Costs (£)			Outcome			ICER
	Incre. dif.	95% CI		Incre. dif.	95% CI		
Base case	48	40	56	0.0143	0.0079	0.0207	3,355
S1: CCA	54	45	63	0.0140	0.0073	0.0208	3,842
S2: Per-protocol	50	40	60	0.0160	0.0090	0.0229	3,112
S3: Up to T3 (1 year)	47	35	58	0.0305	0.0179	0.0432	1,525
S4: CORE-OM T2 CE	48	40	56	0.3	0.3	0.2	188
S5: WEMWBS T2 CE	48	40	56	3.0	1.6	4.5	16

For peer review only - http://bmjopen.bmj.com/site/about/guidelines.xhtml

4 Discussion

4.1 Main findings

There is strong evidence from here and elsewhere [13,14] that MSS leads to significantly improved outcomes. These improved outcomes come at a moderate cost: a mean additional cost of around £50 per user if mindfulness is delivered as it was here, with 30 participants per group. There are no agreed thresholds for this setting to determine whether the gains and additional costs would be considered cost-effective; however, compared to NICE thresholds (eg £20,000/QALY), MSS+SAU would be considered cost-effective compared to SAU. The uncertainty around this decision again depends on the chosen threshold: at NICE thresholds, there is near certainty that MSS+SAU is cost-effective; also, near certainty is reached for what most would likely consider low willingness-to-pay values (see Figure 2).

However, as covered below in the Study strengths and weaknesses section, this economic evaluation does not measure some of the wider costs and benefits of improving student health – such as potentially reduced use of university healthcare services (less cost to the university) and increasing security of tuition fee income (fees not lost through student drop-out). The importance of the latter point is touched on by Simpson: "As the costs and benefits of higher education become an increasingly important economic issue for students, institutions and governments, student retention will correspondingly gather increasing importance as a vital aspect of the economic analysis of higher education" [p.34-35, 41]. Additionally, though perhaps less tangibly, a stronger student mental health offering, may promote student satisfaction and institutional reputation, key factors for student recruitment, in the increasingly competitive higher education setting.

4.2 Comparison with other studies

Systematic reviews of mindfulness based interventions for students have broadly been in favour of mindfulness improving student mental health [42–44]. Evaluations of such interventions have generally been in RCTs, but have been noted to vary in quality, typically with a high risk of bias, limited follow-up and with little consideration of safety [42–44]. In contrast, the RCT from which this economic evaluation draws data has a lower than average risk of bias, followed students for up to 12 months, and reported on safety [13]. A more recent study has also found benefits in favour of mindfulness based interventions improving student mental health [45].

Turning to economic evaluations of mindfulness based interventions, we are unaware of any others in university settings. Recent systematic reviews of economic evaluations [46,47] of mindfulness-related interventions show a near exclusive focus on clinical populations, particularly for relapse prevention in major depression (but other populations are also considered eg [48]). These reviews consider a range of interventions which incorporate mindfulness training to varying degrees; most comparable to this trial's interventions are mindfulness-based cognitive therapy (MBCT) and mindfulness-based stress reduction (MBSR). Both reviews conclude similarly for MBCT that 'findings are inconclusive because of the presence of very positive results in some trials … and modest

For peer review only - http://bmjopen.bmj.com/site/about/guidelines.xhtml

results in others' (p.143) [46]. There was only one economic evaluation of an MBSR intervention [49], with an ICER of $22,200/QALY (international dollars at 2014 prices) which the authors considered within the threshold of cost-effectiveness.

We are aware of only two articles that explore the economic impact of mindfulness outside of clinical populations [50,51]. Dongel et al. [50] conducted a 'cost-effectiveness analysis and return-on-investment analysis comparing mindfulness based work site intervention to usual practice' (p.550) for governmental research institute employees. Usual practice 'dominated' the mindfulness intervention: for all outcomes considered (work engagement, general vitality and work ability) scores were statistically significant in favour of usual practice, and costs lower for this group from both employer and societal perspectives. Accordingly, the return on investment was negative. These negative findings are suggested to be due to the 'participants' low compliance with some of the intervention components' (p.555). MSS participants' intervention attendance was not dissimilar to theirs, although MSS participants appear to have been slightly more compliant with homework [13].

Over five years, Klatt et al. [51] compares healthcare costs (to insurers) and utilisation by university staff who received one of two interventions (one of them mindfulness) compared to matched controls: compared to controls, costs were lower for both of the interventions. There was no economic evaluation in the original trial [52], but the interventions were suggested to be 'low-cost' [52] while unlikely to be funded by the insurer [51].

4.3 Study strengths and weaknesses

Contrasting with many studies in this area (see Section 4.2, a key strength of this economic evaluation is that it drew on data from a large ($n > 600$) RCT that followed-up participants for up to 12 months. Additionally, to our knowledge, it is the only economic evaluation of a mindfulness intervention for students. The choice of measures used allowed QALYs to be calculated.

A key weakness of the study is the very narrow costing perspective adopted – that of the UCS. This perspective likely captures the majority of costs of directly delivering the intervention, but may well miss wider impacts. A natural perspective to adopt would be that of the university, but collecting required information for such a wide and multi-domain perspective (for example, details on tuition fees and use of other student support services) would be very challenging.

A further weakness is that in costing we have focused exclusively on the costs of staff time, as measured by salary plus 'on-costs' (e.g. such as employers' pension and national insurance costs – see £ Section 6.5). We have not considered wider overheads or other resources (such as room hire), which will underestimate costs. In contrast to the NHS (for instance [53]), we are not aware of any pre-existing cost sources. A potentially key cost exclusion, particularly given the group based nature of the intervention, is the cost of room hire. While the UCS' operating budget does not incur a cost for room hire, there is of course a potential 'opportunity cost', by which we mean that if the rooms were not being used for MSS, they could potentially be used for something else, such as teaching. We would expect

For peer review only - http://bmjopen.bmj.com/site/about/guidelines.xhtml

the opportunity cost to be lower where there is less 'competition' for rooms - for example, if mindfulness were delivered in the evenings, when rooms are not required for teaching.

The primary analysis was a cost-utility analysis [32] where the ICER corresponds to cost per additional QALY. Interpreting the ICER is difficult in this sector as there are no established cost-effectiveness thresholds (see Section 2.4). As argued by [54], policy makers may benefit from a different analysis such as cost-benefit analysis (CBA) which 'is a comparison of interventions and their consequences in which both costs and resulting benefits (health outcomes and others) are expressed in monetary terms' [55]. Alternatives can then compared using the net monetary benefit 'which is the difference between the benefit of each treatments (expressed in monetary units) less the cost of each' [55]. However, valuing benefits in monetary terms as required by CBA is demanding, and arguably particularly so in this setting where benefits occur in many diverse domains.

5 Conclusions

This economic evaluation has shown MSS+SAU accrue more benefits than SAU, but these increased benefits come at a greater cost. The main driver of these increased costs were the costs of delivering the MSS course. Whether these benefits are considered to be worth the additional cost depends on the cost-effectiveness threshold adopted - this is not established for the university sector. Compared to the NICE threshold for the NHS, MSS+SAU would be considered highly cost-effective compared to SAU.

Contributorship statement

GD conceived the mindfulness intervention pilot on behalf of the UCS and concurrent research into the effectiveness of the provision. PBJ and GD applied for funding for the underlying randomised controlled trial. JG and PBJ produced an initial draft of the overall study protocol that was revised through discussion with all the authors. APW and GB produced an initial draft of the health economic plan which was revised through discussion with all authors. JG, GD, MV, and PBJ did the study, with input from the other authors. APW led the health economic analysis, with input from JG, GB & JS. APW led drafting of this article, which was read, reviewed and approved by the other authors. PBJ is study guarantor.

Competing interests

The authors have no conflicts of interest to declare.

Funding

This is a summary of research funded by the University of Cambridge Vice-Chancellor's Endowment Fund, the University Counselling Service and the National Institute for Health and Care Research (NIHR) Applied Research Collaboration East of England (ARC EoE) programme. The views expressed are those of the authors and not necessarily those of the University of Cambridge, NHS, NIHR or Department of Health and Social Care.

For peer review only - http://bmjopen.bmj.com/site/about/guidelines.xhtml

Data sharing statement

De-identified individual participant data and dictionary are available for researchers upon request from the corresponding author after approval of a proposal, with a signed data access agreement.

Ethics approval statement

This study was approved by the Cambridge Psychology Research Ethics committee on August 25, 2015 (reference: PRE.2015.060).

Acknowledgements

We thank the study participants, the mindfulness teacher Elizabeth English for her development of the intervention independently of the researchers, the administrative and senior management team at the UCS, Alice Benton, and Emma Howarth.

References

1 Global Burden of Disease 2019 Diseases and Injuries Collaborators. Global burden of 369 diseases and injuries in 204 countries and territories, 1990–2019: A systematic analysis for the global burden of disease study 2019. *Lancet* 2020;**369**:1204–22. doi:10.1016/S0140-6736(20)30925-9

2 NHS Digital. Mental health of children and young people in england 2022 - wave 3 follow up to the 2017 survey. 2022.https://digital.nhs.uk/data-and-information/publications/statistical/mental-health-of-children-and-young-people-in-england/2022-follow-up-to-the-2017-survey

3 Mair D. Short-term counselling in higher education—context, theory and practice. Abingdon: Routledge 2016. 7–26.

4 Thorley C. Not by degrees: Improving student mental health in the UK's universities. 2017. https://www.ippr.org/research/publications/not-by-degrees

5 Rith-Najarian LR, Boustani MM, Chorpita BF. A systematic review of prevention programs targeting depression, anxiety, and stress in university students. *Journal of Affective Disorders* 2019;**257**:568–84. http://www.sciencedirect.com/science/article/pii/S016503271832754X

6 Lattie EG, Lipson SK, Eisenberg D. Technology and college student mental health: Challenges and opportunities. *Frontiers in psychiatry* 2019;**10**:246–6. https://www.ncbi.nlm.nih.gov/pmc/articles/PMC6476258/

7 Lo Moro G, Soneson E, Jones PB, *et al.* Establishing a theory-based multi-level approach for primary prevention of mental disorders in young people. *International Journal of Environmental Research and Public Health* 2020;**17**. doi:10.3390/ijerph17249445

For peer review only - http://bmjopen.bmj.com/site/about/guidelines.xhtml

8 Kabat-Zinn J. Mindfulness-based interventions in context: Past, present, and future. *Clinical Psychology: Science and Practice* 2003;**10**:144–56. doi:10.1093/clipsy.bpg016

9 Mindfulness for Students. Mindfulness courses at UK universities. 2018. http://mindfulnessforstudents.co.uk/mindfulness-courses-at-uk-universities

10 Mindfulness All-Party Parliamentary Group. Mindful Nation UK Report: The Mindfulness Initiative. 2015. https://www.themindfulnessinitiative.org/mindful-nation-report

11 Dufour G. Current challenges in student mental health and counselling provision: How practice-based research can help demonstrate effectiveness. *Counselling and Psychotherapy Research* 2020;1–6. doi:10.1002/capr.12310

12 Galante J, Dufour G, Benton A, *et al.* Protocol for the mindful student study: A randomised controlled trial of the provision of a mindfulness intervention to support university students' well-being and resilience to stress. *BMJ Open* 2016;**6**:e012300. doi:10.1136/bmjopen-2016-012300

13 Galante J, Dufour G, Vainre M, *et al.* A mindfulness-based intervention to increase resilience to stress in university students (the mindful student study): A pragmatic randomised controlled trial. *The Lancet Public Health* 2018;**3**:e72–81. doi:10.1016/s2468-2667(17)30231-1

14 Galante J, Stochl J, Dufour G, *et al.* Effectiveness of providing university students with a mindfulness-based intervention to increase resilience to stress: 1-year follow-up of a pragmatic randomised controlled trial. *Journal of Epidemiology & Community Health* 2021;**75**:151–60. doi:10.1136/jech-2020-214390

15 Russ TC, Stamatakis E, Hamer M, *et al.* Association between psychological distress and mortality: Individual participant pooled analysis of 10 prospective cohort studies. *BMJ* 2012;**345**. doi:10.1136/bmj.e4933

16 Wagner AP, Barton G. Mindfulness student study (MSS): Health economic analysis plan (HEAP). University of East Anglia; Internet 2018. https://www.anzctr.org.au/Steps11and12/369222-(Uploaded-23-08-2019-20-02-35)-Study-related document.pdf

17 CORE System Group. CORE system user manual. 2015. http://www.coreims.co.uk/index.html

18 Evans C, Connell J, Barkham M, *et al.* Towards a standardised brief outcome measure: Psychometric properties and utility of the CORE-OM. *The British journal of psychiatry : the journal of mental science* 2002;**180**:51–60.

19 Connell J, Barkham M, Mellor-Clark J. The effectiveness of UK student counselling services: An analysis using the CORE system. *British Journal of Guidance & Counselling* 2008;**36**:1–18. doi:10.1080/03069880701715655

For peer review only - http://bmjopen.bmj.com/site/about/guidelines.xhtml

20 York Health Economics Consortium. Preference-based measures. 2016. https://yhec.co.uk/glossary/preference-based-measures/

21 Mavranezouli I, Brazier JE, Rowen D, *et al.* Estimating a preference-based index from the clinical outcomes in routine evaluation-outcome measure (CORE-OM): Valuation of CORE-6D. *Medical decision making : an international journal of the Society for Medical Decision Making* 2013;**33**:381–95. https://www.ncbi.nlm.nih.gov/pmc/articles/PMC4107796/

22 Mavranezouli I, Brazier JE, Young TA, *et al.* Using rasch analysis to form plausible health states amenable to valuation: The development of CORE-6D from a measure of common mental health problems (CORE-OM). *Quality of life research* 2011;**20**:321–33.

23 Manca A, Hawkins N, Sculpher MJ. Estimating mean QALYs in trial-based cost-effectiveness analysis: The importance of controlling for baseline utility. *Health economics* 2005;**14**:487–96.

24 Stewart-Brown S, Janmohamed K. Warwick-Edinburgh Mental Well-Being Scale (WEMWBS). User guide: version 1. 2008. http://www.mentalhealthpromotion.net/resources/user-guide.pdf

25 Tennant R, Hiller L, Fishwick R, *et al.* The warwick-edinburgh mental well-being scale (WEMWBS): Development and UK validation. *Health and quality of life outcomes* 2007;**5**:63.

26 Noble SM, Hollingworth W, Tilling K. Missing data in trial-based cost-effectiveness analysis: The current state of play. *Health Econ* 2012;**21**:187–200. doi:10.1002/hec.1693

27 Faria R, Gomes M, Epstein D, *et al.* A guide to handling missing data in cost-effectiveness analysis conducted within randomised controlled trials. *PharmacoEconomics* 2014;**32**:1157–70.

28 White IR, Royston P, Wood AM. Multiple imputation using chained equations: Issues and guidance for practice. *Statist Med* 2011;**30**:377–99. doi:10.1002/sim.4067

29 van Buuren S, Groothuis-Oudshoorn K. mice: Multivariate imputation by chained equations in r. *Journal of Statistical Software* 2011;**45**:1–67. https://www.jstatsoft.org/v45/i03/

30 Little RJA, Rubin DB. *Statistical analysis with missing data*. 2nd ed. Hoboken, NJ: : Wiley 2002.

31 Willan AR, Briggs AH, Hoch JS. Regression methods for covariate adjustment and subgroup analysis for non-censored cost-effectiveness data. *Health economics* 2004;**13**:461–75.

32 Drummond MF, Sculpher MJ, Claxton K, *et al. Methods for the economic evaluation of health care programmes*. 4th ed. Oxford University Press, Oxford 2015.

For peer review only

For peer review only - http://bmjopen.bmj.com/site/about/guidelines.xhtml

33 NICE. NICE - the national institute for HEalth and care excellence. 2020.

34 NICE. *Guide to the methods of technology appraisal*. National Institute of Health; Clincal Excellence, London 2013.

35 Briggs AH, O'Brien BJ, Blackhouse G. Thinking outside the box: Recent advances in the analysis and presentation of uncertainty in cost-effectiveness studies. *Annual review of public health* 2002;**23**:377–401.

36 Black WC. The CE plane: A graphic representation of cost-effectiveness. *Medical decision making : an international journal of the Society for Medical Decision Making* 1990;**10**:212–4.

37 Fenwick E, O'Brien BJ, Briggs A. Cost-effectiveness acceptability curves–facts, fallacies and frequently asked questions. *Health economics* 2004;**13**:405–15.

38 R Core Team. *R: A language and environment for statistical computing*. Vienna, Austria: : R Foundation for Statistical Computing 2019. https://www.R-project.org/

39 Stiles W, Barkham M, Wheeler S. Duration of psychological therapy: Relation to recovery and improvement rates in UK routine practice [corrected]. *Br J Psychiatry* 2015;**207**:115–22. doi:10.1192/bjp.bp.114.145565

40 Connell J, Barkham M, Stiles WB, *et al.* Distribution of CORE–OM scores in a general population, clinical cut-off points and comparison with the CIS–r. *The British Journal of Psychiatry* 2007;**190**:69–74. doi:10.1192/bjp.bp.105.017657

41 Simpson O. The costs and benefits of student retention for students, institutions and governments. *Studies in Learning, Evaluation Innovation and Development* 2005;**2**:34–43.https://oro.open.ac.uk/6761/1/SLEID_costs_and_benefits.pdf

42 Chiodelli R, Mello LTN de, Jesus SN de, *et al.* Mindfulness-based interventions in undergraduate students: A systematic review. *Journal of American College Health* 2020;1–10. doi:10.1080/07448481.2020.1767109

43 Breedvelt JJF, Amanvermez Y, Harrer M, *et al.* The effects of meditation, yoga, and mindfulness on depression, anxiety, and stress in tertiary education students: A meta-analysis. *Frontiers in psychiatry* 2019;**10**:193–3. https://www.ncbi.nlm.nih.gov/pmc/articles/PMC6491852/

44 Dawson AF, Brown WW, Anderson J, *et al.* Mindfulness-based interventions for university students: A systematic review and meta-analysis of randomised controlled trials. *Applied psychology Health and well-being* 2019.

45 Juul L, Brorsen E, Gøtzsche Katinka, *et al.* The effects of a mindfulness program on mental health in students at an undergraduate program for teacher education: A randomized controlled trial in real-life. *Frontiers in Psychology* 2021;**12**. doi:10.3389/fpsyg.2021.722771

For peer review only - http://bmjopen.bmj.com/site/about/guidelines.xhtml

46 Duarte R, Lloyd A, Kotas E, *et al.* Are acceptance and mindfulness-based interventions "value for money"? Evidence from a systematic literature review. *British Journal of Clinical Psychology* 2019;**58**:187–210. doi:10.1111/bjc.12208

47 Feliu-Soler A, Cebolla A, McCracken LM, *et al.* Economic impact of third-wave cognitive behavioral therapies: A systematic review and quality assessment of economic evaluations in randomized controlled trials. *Behavior Therapy* 2018;**49**:124–47. http://www.sciencedirect.com/science/article/pii/S000578941730076X

48 Fjorback LO, Carstensen T, Arendt M, *et al.* Mindfulness therapy for somatization disorder and functional somatic syndromes: Analysis of economic consequences alongside a randomized trial. *Journal of Psychosomatic Research* 2013;**74**:41–8. doi:https://doi.org/10.1016/j.jpsychores.2012.09.010

49 Lengacher CA, Kip KE, Reich RR, *et al.* A cost-effective mindfulness stress reduction program: A randomized control trial for breast cancer survivors. *Nursing economic* 2015;**33**:210–8, 232.

50 Dongen JM van, Berkel J van, Boot CRL, *et al.* Long-term cost-effectiveness and return-on-investment of a mindfulness-based worksite intervention: Results of a randomized controlled trial. *Journal of occupational and environmental medicine* 2016;**58**:550–60.

51 Klatt MD, Sieck C, Gascon G, *et al.* A healthcare utilization cost comparison between employees receiving a worksite mindfulness or a diet/exercise lifestyle intervention to matched controls 5 years post intervention. *Complementary therapies in medicine* 2016;**27**:139–44.

52 Malarkey WB, Jarjoura D, Klatt M. Workplace based mindfulness practice and inflammation: A randomized trial. *Brain, behavior, and immunity* 2013;**27**:145–54.

53 Curtis L, Burns A, editors. *Unit costs of health and social care 2019*. Canterbury: : Personal Social Services Research Unit, University of Kent 2018. doi:10.22024/UniKent/01.02.79286

54 Tudor Edwards R, Bryning L, Crane R. Design of economic evaluations of mindfulness-based interventions: Ten methodological questions of which to be mindful. *Mindfulness* 2015;**6**:490–500. https://www.ncbi.nlm.nih.gov/pmc/articles/PMC4432017/

55 York Health Economics Consortium. Cost-benefit analysis. 2016. https://yhec.co.uk/glossary/cost-benefit-analysis/

For peer review only - http://bmjopen.bmj.com/site/about/guidelines.xhtml

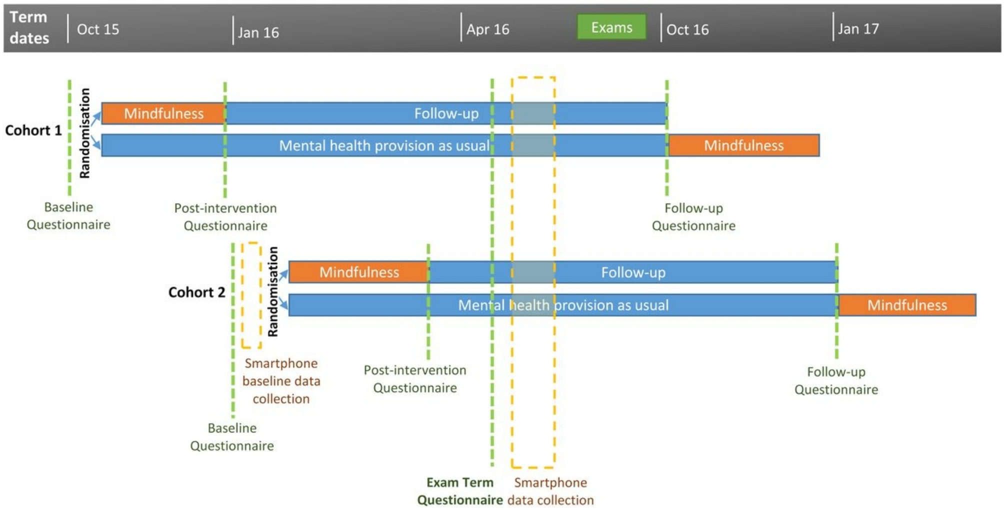

Participant timeline. Reproduced from Galante et al. (2016)

108x54mm (300 x 300 DPI)

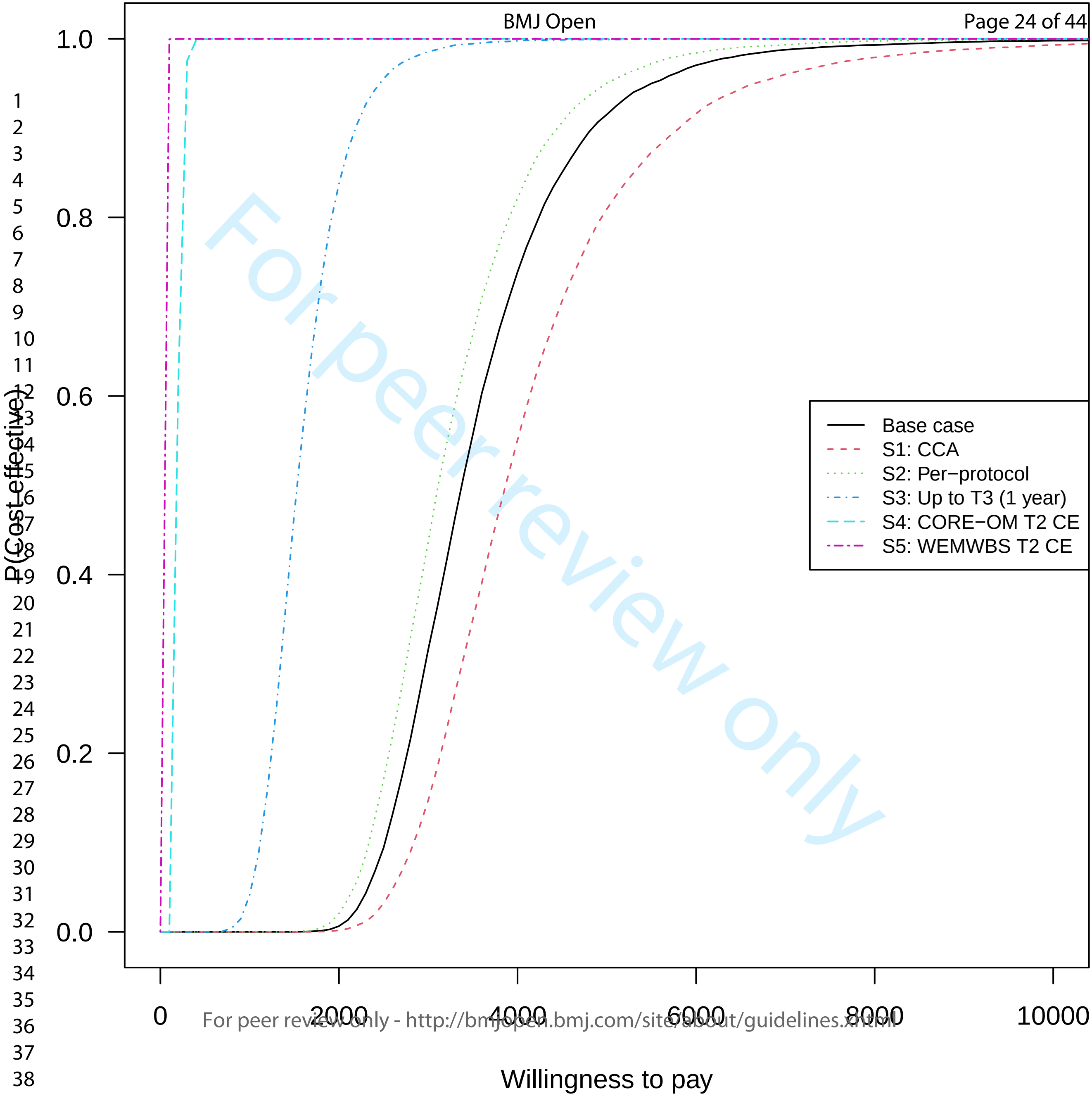

For peer review only

Cost-effectiveness of providing university students with a mindfulness-based intervention to reduce psychological distress: economic evaluation of a pragmatic randomised controlled trial
SUPPLEMENTARY FILE

Adam P Wagner, Julieta Galante, Géraldine Dufour, Garry Barton, Jan Stochl, Maris Vainre, and Peter B Jones

12/09/2023

Adam P Wagner, PhD, (Corresponding author) Senior Research Fellow, Norwich Medical School, University of East Anglia, Norwich Research Park, Norwich, NR4 7TJ, United Kingdom (second affiliation: National Institute for Health Research (NIHR) Applied Research Collaboration (ARC) East of England (EoE)). adam.wagner@uea.ac.uk

Julieta Galante, PhD, Honorary Senior Visiting Research Fellow, Department of Psychiatry, University of Cambridge, Douglas House, 18b Trumpington Road, Cambridge, CB2 8AH, United Kingdom (second affiliation: Contemplative Studies Centre, Melbourne School of Psychological Sciences, Faculty of Medicine, Dentistry, and Health Sciences, University of Melbourne). mjg231@cam.ac.uk phone (+44) 7986 172834

Géraldine Dufour, MA, Geraldine Dufour Therapeutic Consultations Ltd https://www.geraldinedufour.com/ (second affiliation: Associate Executive Member, European Association for International Education (EAIE)).

Garry Barton, PhD, Professor of Health Economics, Norwich Medical School, University of East Anglia, Norwich Research Park, Norwich, NR4 7TJ, United Kingdom.

Jan Stochl, PhD, Senior Research Associate, Department of Psychiatry, University of Cambridge, Cambridge Biomedical Campus, Box 189, Cambridge, CB2 0QQ, United Kingdom (second affiliation: Department of Kinanthropology, Charles University, Jose Martiho 31, 162 52 Prague, Czech Republic; third affiliation: NIHR ARC EoE).

Maris Vainre, MA, PhD Student, MRC Cognition and Brain Sciences Unit, University of Cambridge, 15 Chaucer Rd, Cambridge, CB2 7EF, United Kingdom (former affiliation when doing this work: NIHR ARC EoE). Maris.Vainre@mrc-cbu.cam.ac.uk

Peter B Jones, PhD, Professor of Psychiatry, Department of Psychiatry, University of Cambridge, Herchel Smith Building, Cambridge, CB2 0SZ, United Kingdom (second affiliation: NIHR ARC EoE).

For peer review only - http://bmjopen.bmj.com/site/about/guidelines.xhtml

6 Supplementary materials

6.1 Missingness by scale and time point

Table 6.1: Missingness (percentage) by outcomes at each time, for each arm and overall.

	Percentage missing		
Outcome	MSS+SAU	SAU	Overall
CORE-6D, T0	0	1	0
CORE-6D, T1	18	27	22
CORE-6D, T2	24	30	27
CORE-6D, T3	45	45	45
CORE-OM, T0	3	4	3
CORE-OM, T1	19	28	23
CORE-OM, T2	26	30	28
CORE-OM, T3	46	46	46
WEMWBS, T0	1	0	0
WEMWBS, T1	18	28	23
WEMWBS, T2	24	30	27
WEMWBS, T3	46	46	46

For peer review only - http://bmjopen.bmj.com/site/about/guidelines.xhtml

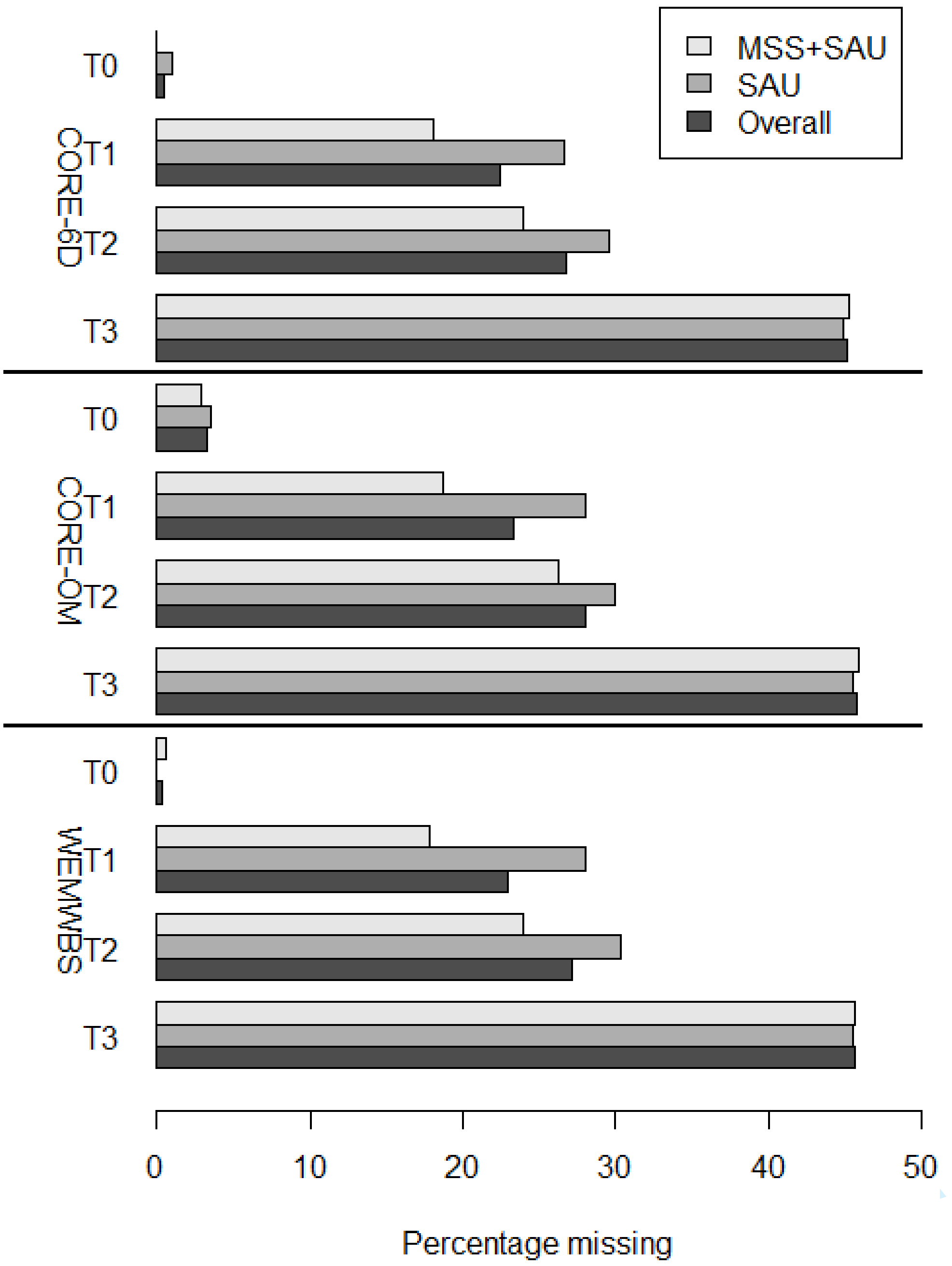

Figure 6.1: Missingness (percentage) by outcomes at each time, for each arm and overall.

For peer review only - http://bmjopen.bmj.com/site/about/guidelines.xhtml

6.2 Patterns of missingness and imputation model

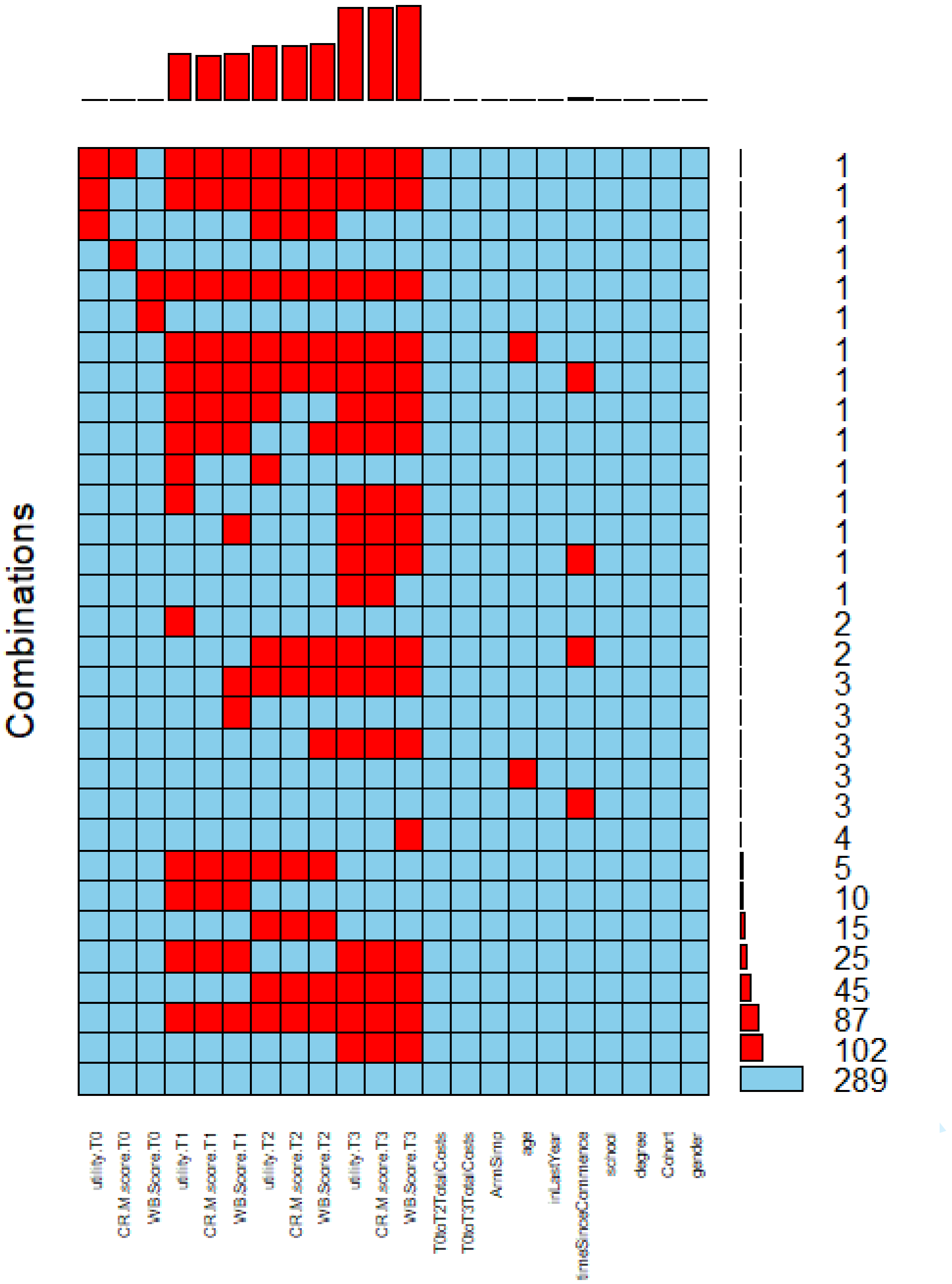

Figure 6.2: Patterns of missingness (red indicates missing) and variables included in imputation model.

6.3 Trial CONSORT diagram

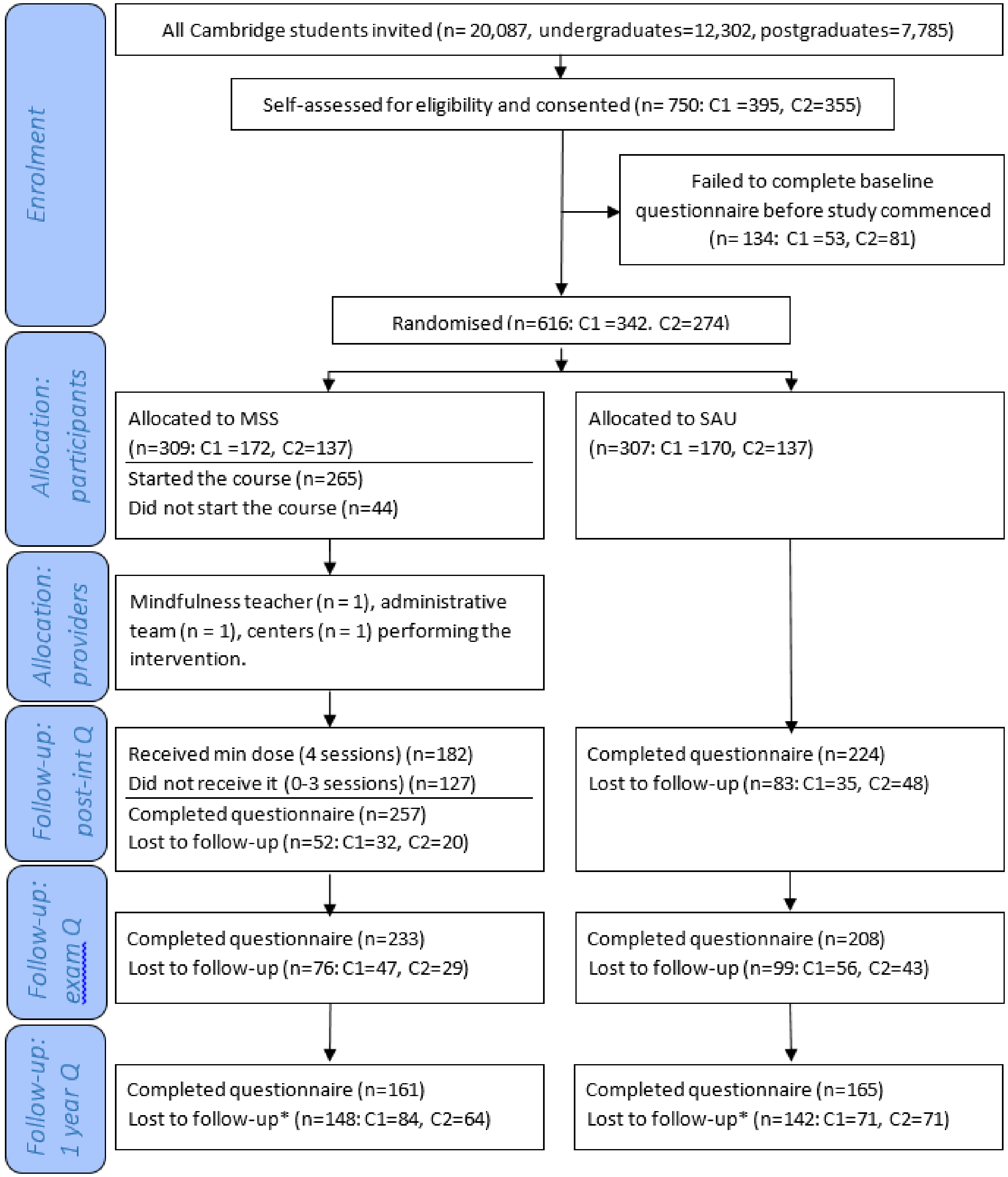

Figure 6.3: CONSORT 2010 diagram taken from Galante et al. (2021)

For peer review only - http://bmjopen.bmj.com/site/about/guidelines.xhtml

6.4 Resource use T0 to T2: Further detail

Table 6.2: Resource use across all participants, T0 to T2 (mean use per student)

	MSS+SAU (n=309)		SAU (n=307)		
Resource use T0 to T2	n/Mean	SD	n/Mean	SD	P-value
Assessment	0.09	0.30	0.11	0.33	0.5937
Counselling	0.30	1.31	0.47	1.70	0.1875
Client Contact by Phone or Email	0.00	0.00	0.00	0.00	-
MHA Assessment	0.01	0.08	0.01	0.08	0.9948
MHA Ongoing	0.00	0.06	0.03	0.36	0.2741
Pre Group	0.02	0.13	0.02	0.13	0.9918
Can't Work Group	0.01	0.08	0.00	0.00	0.1576
Returners - Anxiety	0.00	0.00	0.00	0.00	-
Returners - Self Compassion	0.00	0.00	0.00	0.00	-
Returners - Time Management	0.00	0.00	0.00	0.00	-
Workshop - Anxiety	0.01	0.10	0.01	0.08	0.6591
Workshop - CBT for self-help	0.00	0.06	0.00	0.06	0.9963
Workshop - Exam Preparation	0.00	0.06	0.00	0.06	0.9963
Workshop - Food and mood	0.01	0.11	0.00	0.00	0.0453
Workshop - Procrastination	0.01	0.11	0.02	0.13	0.7302
Workshop - Self-compassion	0.01	0.11	0.01	0.14	0.9934
Workshop - Sleep	0.00	0.00	0.00	0.00	-
Workshop - Social anxiety	0.00	0.00	0.00	0.00	-
Workshop - Panic attacks	0.01	0.08	0.00	0.00	0.1576
Assertiveness Group	0.01	0.08	0.00	0.06	0.5669
Bereavement Group	0.00	0.06	0.01	0.08	0.5601
M Phil Group	0.00	0.00	0.00	0.00	-
Managing Mood Group	0.00	0.06	0.00	0.00	0.3181
Perfectionism Group	0.00	0.00	0.00	0.06	0.3181
Post Graduate Group	0.00	0.00	0.00	0.00	-
Returners Group	0.01	0.08	0.00	0.06	0.5669
Self-esteem Group	0.00	0.06	0.00	0.06	0.9963
UG Group	0.00	0.06	0.00	0.00	0.3181

For peer review only - http://bmjopen.bmj.com/site/about/guidelines.xhtml

*Table 6.3: Resource use across participants *using* a particular service T0 to T2 (eg means here exclude participants not using that UCS offering)*

	MSS+SAU (n=309)		SAU (n=307)	
	Used (N)	Used mean	Used (N)	Used mean
Assessment	28	1.04	31	1.06
Counselling	27	3.48	37	3.86
Client Contact by Phone or Email	0	-	0	-
MHA Assessment	2	1.00	2	1.00
MHA Ongoing	1	1.00	2	4.00
Pre Group	5	1.00	5	1.00
Can't Work Group	2	1.00	0	-
Returners - Anxiety	0	-	0	-
Returners - Self Compassion	0	-	0	-
Returners - Time Management	0	-	0	-
Workshop - Anxiety	3	1.00	2	1.00
Workshop - CBT for self-help	1	1.00	1	1.00
Workshop - Exam Preparation	1	1.00	1	1.00
Workshop - Food and mood	4	1.00	0	-
Workshop - Procrastination	4	1.00	5	1.00
Workshop - Self-compassion	4	1.00	3	1.33
Workshop - Sleep	0	-	0	-
Workshop - Social anxiety	0	-	0	-
Workshop - Panic attacks	2	1.00	0	-
Assertiveness Group	2	1.00	1	1.00
Bereavement Group	1	1.00	2	1.00
M Phil Group	0	-	0	-
Managing Mood Group	1	1.00	0	-
Perfectionism Group	0	-	1	1.00
Post Graduate Group	0	-	0	-
Returners Group	2	1.00	1	1.00
Self-esteem Group	1	1.00	1	1.00
UG Group	1	1.00	0	-

For peer review only - http://bmjopen.bmj.com/site/about/guidelines.xhtml

6.5 Detailed unit costs

Table 6.4: Details of wage costs

Grade	Spine point	Salary (£)	Pension (£)	NI (£)	Apprent levy (£)	Total (£)	Rate (£/hr)
4	32	25,627	5,535	2,487	128	33,777	17.71
7	44	36,382	7,859	4,106	181	48,528	25.45

For peer review only

Table 6.5: Unit costs for each of the UCS offerings.

Type	Activity	Duration (mins)	Student contact (mins)	Occurrences	Group size	Total cost (£)	Unit cost (£)
Individual	Assessment	100	60	1	1	42.42	42.42
	Counselling	65	60	1	1	27.57	27.57
	Client Contact by Phone or Email	25	20	1	1	10.60	10.60
	MHA Assessment	100	60	1	1	42.42	42.42
	MHA Ongoing	65	60	1	1	27.57	27.57
	Pre Group	35	30	1	1	14.85	14.85
Workshop	Can't Work Group	135	120	1	6	57.26	9.54
	Returners - Anxiety	135	90	1	20	57.26	2.86
	Returners - Self Compassion	135	120	1	20	57.26	2.86
	Returners - Time Management	135	120	1	20	57.26	2.86
	Workshop - Anxiety	135	120	1	15	57.26	3.82
	Workshop - CBT for self-help	135	120	1	15	57.26	3.82
	Workshop - Exam Preparation	135	120	1	15	57.26	3.82
	Workshop - Food and mood	135	120	1	15	57.26	3.82
	Workshop - Procrastination	135	90	1	15	57.26	3.82
	Workshop - Self-compassion	135	120	1	15	57.26	3.82
	Workshop - Sleep	135	90	1	15	57.26	3.82
	Workshop - Social anxiety	135	120	1	15	57.26	3.82
	Workshop - Panic attacks	135	120	1	15	57.26	3.82
Group	Assertiveness Group	135	120	4	8	229.05	28.63
	Bereavement Group	135	120	6	8	343.57	42.95
	M Phil Group	135	120	5	8	286.31	35.79
	Managing Mood Group	135	120	6	8	343.57	42.95
	Perfectionism Group	135	120	5	8	286.31	35.79
	Post Graduate Group	135	120	14	8	801.67	100.21
	Returners Group	135	120	1	20	57.26	2.86
	Self-esteem Group	135	90	5	8	286.31	35.79
	UG Group	135	120	10	8	572.62	71.58

For peer review only - http://bmjopen.bmj.com/site/about/guidelines.xhtml

6.6 Costs T0 to T2: Further detail

Table 6.6: Mean costs per student across all participants, T0 to T2

	MSS+SAU (n=309)		SAU (n=307)		
UCS activity costs T0 to T2	n/Mean (£)	SD (£)	n/Mean (£)	SD (£)	P-value
Assessment	3.98	12.85	4.56	14.02	0.5937
Counselling	8.39	36.15	12.84	46.92	0.1875
Client Contact by Phone or Email	0.00	0.00	0.00	0.00	-
MHA Assessment	0.27	3.41	0.28	3.42	0.9948
MHA Ongoing	0.09	1.57	0.72	9.94	0.2741
Pre Group	0.24	1.88	0.24	1.88	0.9918
Can't Work Group	0.06	0.77	0.00	0.00	0.1576
Returners - Anxiety	0.00	0.00	0.00	0.00	-
Returners - Self Compassion	0.00	0.00	0.00	0.00	-
Returners - Time Management	0.00	0.00	0.00	0.00	-
Workshop - Anxiety	0.04	0.37	0.02	0.31	0.6591
Workshop - CBT for self-help	0.01	0.22	0.01	0.22	0.9963
Workshop - Exam Preparation	0.01	0.22	0.01	0.22	0.9963
Workshop - Food and mood	0.05	0.43	0.00	0.00	0.0453
Workshop - Procrastination	0.05	0.43	0.06	0.48	0.7302
Workshop - Self-compassion	0.05	0.43	0.05	0.53	0.9934
Workshop - Sleep	0.00	0.00	0.00	0.00	-
Workshop - Social anxiety	0.00	0.00	0.00	0.00	-
Workshop - Panic attacks	0.02	0.31	0.00	0.00	0.1576
Assertiveness Group	0.19	2.30	0.09	1.63	0.5669
Bereavement Group	0.14	2.44	0.28	3.46	0.5601
M Phil Group	0.00	0.00	0.00	0.00	-
Managing Mood Group	0.14	2.44	0.00	0.00	0.3181
Perfectionism Group	0.00	0.00	0.12	2.04	0.3181
Post Graduate Group	0.00	0.00	0.00	0.00	-
Returners Group	0.02	0.23	0.01	0.16	0.5669
Self-esteem Group	0.12	2.04	0.12	2.04	0.9963
UG Group	0.23	4.07	0.00	0.00	0.3181

For peer review only - http://bmjopen.bmj.com/site/about/guidelines.xhtml

6.7 Resource use T0 to T3

Table 6.7: Aggregated UCS resource use by arm between T0 to T2. Means are the amount of each activity by student. SD= standard deviation

	MSS+SAU (n=309)		SAU (n=307)		
Resource use T0 to T3	n/Mean	SD	n/Mean	SD	P-value
Used UCS services: N=	64		73		
Individual assessment sessions	0.15	0.39	0.17	0.40	0.6607
Individual counselling sessions	0.52	1.76	0.72	2.68	0.2773
Total other individual activity	0.04	0.20	0.08	0.54	0.1375
Total workshops	0.07	0.34	0.05	0.26	0.3611
Total groups	0.03	0.18	0.03	0.16	0.6428

For peer review only - http://bmjopen.bmj.com/site/about/guidelines.xhtml

Table 6.8: Resource use across all participants, T0 to T3 (mean use per student)

	MSS+SAU (n=309)		SAU (n=307)		
Resource T0 to T3	n/Mean	SD	n/Mean	SD	P-value
Assessment	0.15	0.39	0.17	0.40	0.6607
Counselling	0.52	1.76	0.72	2.68	0.2773
Client Contact by Phone or Email	0.00	0.00	0.00	0.06	0.3181
MHA Assessment	0.01	0.10	0.02	0.14	0.3104
MHA Ongoing	0.00	0.06	0.04	0.47	0.1454
Pre Group	0.02	0.15	0.02	0.14	0.7887
Can't Work Group	0.01	0.11	0.01	0.08	0.4169
Returners - Anxiety	0.00	0.00	0.00	0.00	-
Returners - Self Compassion	0.00	0.00	0.00	0.00	-
Returners - Time Management	0.00	0.00	0.00	0.00	-
Workshop - Anxiety	0.01	0.10	0.01	0.08	0.6591
Workshop - CBT for self-help	0.00	0.06	0.00	0.06	0.9963
Workshop - Exam Preparation	0.00	0.06	0.00	0.06	0.9963
Workshop - Food and mood	0.01	0.11	0.00	0.00	0.0453
Workshop - Procrastination	0.01	0.11	0.02	0.13	0.7302
Workshop - Self-compassion	0.01	0.11	0.01	0.14	0.9934
Workshop - Sleep	0.00	0.00	0.00	0.00	-
Workshop - Social anxiety	0.00	0.00	0.00	0.00	-
Workshop - Panic attacks	0.01	0.08	0.00	0.06	0.5669
Assertiveness Group	0.01	0.10	0.01	0.08	0.6591
Bereavement Group	0.00	0.06	0.01	0.08	0.5601
M Phil Group	0.00	0.00	0.00	0.00	-
Managing Mood Group	0.00	0.06	0.00	0.00	0.3181
Perfectionism Group	0.00	0.00	0.00	0.06	0.3181
Post Graduate Group	0.00	0.06	0.00	0.06	0.9963
Returners Group	0.01	0.08	0.00	0.06	0.5669
Self-esteem Group	0.00	0.06	0.00	0.06	0.9963
UG Group	0.00	0.06	0.00	0.00	0.3181

For peer review only - http://bmjopen.bmj.com/site/about/guidelines.xhtml

*Table 6.9: Resource use across participants *using* a particular service T0 to T3 (eg means here exclude participants not using that UCS offering)*

	MSS+SAU (n=309)		SAU (n=307)	
	Used (N)	Used mean	Used (N)	Used mean
Assessment	43	1.09	48	1.06
Counselling	41	3.93	49	4.51
Client Contact by Phone or Email	0	-	1	1.00
MHA Assessment	3	1.00	6	1.00
MHA Ongoing	1	1.00	3	4.33
Pre Group	7	1.00	6	1.00
Can't Work Group	4	1.00	2	1.00
Returners - Anxiety	0	-	0	-
Returners - Self Compassion	0	-	0	-
Returners - Time Management	0	-	0	-
Workshop - Anxiety	3	1.00	2	1.00
Workshop - CBT for self-help	1	1.00	1	1.00
Workshop - Exam Preparation	1	1.00	1	1.00
Workshop - Food and mood	4	1.00	0	-
Workshop - Procrastination	4	1.00	5	1.00
Workshop - Self-compassion	4	1.00	3	1.33
Workshop - Sleep	0	-	0	-
Workshop - Social anxiety	0	-	0	-
Workshop - Panic attacks	2	1.00	1	1.00
Assertiveness Group	3	1.00	2	1.00
Bereavement Group	1	1.00	2	1.00
M Phil Group	0	-	0	-
Managing Mood Group	1	1.00	0	-
Perfectionism Group	0	-	1	1.00
Post Graduate Group	1	1.00	1	1.00
Returners Group	2	1.00	1	1.00
Self-esteem Group	1	1.00	1	1.00
UG Group	1	1.00	0	-

6.8 Costs T0 to T3

Table 6.10: Mean cost (per student) incurred by the UCS, T0 to T3

	MSS+SAU (n=309)		SAU (n=307)		
Costs T0 to T3	n/Mean (£)	SD (£)	n/Mean (£)	SD (£)	P-value
Individual assessment sessions	6.45	16.72	7.05	16.89	0.6607
Individual counselling sessions	14.37	48.65	19.85	73.82	0.2773
Total other individual activity	0.84	5.65	2.32	15.71	0.1201
Total workshops	0.36	1.66	0.24	1.22	0.2991
Total groups	1.25	8.48	1.04	7.59	0.7448
MSS course	52.82	0.00	0.00	0.00	
Total	76.07	63.27	30.49	84.97	0.0000

For peer review only - http://bmjopen.bmj.com/site/about/guidelines.xhtml

Table 6.11: Mean costs per student across all participants, T0 to T3

	MSS+SAU (n=309)		SAU (n=307)		
UCS activity costs T0 to T3	n/Mean (£)	SD (£)	n/Mean (£)	SD (£)	P-value
Assessment	6.45	16.72	7.05	16.89	0.6607
Counselling	14.37	48.65	19.85	73.82	0.2773
Client Contact by Phone or Email	0.00	0.00	0.03	0.61	0.3181
MHA Assessment	0.41	4.17	0.83	5.88	0.3104
MHA Ongoing	0.09	1.57	1.17	12.85	0.1454
Pre Group	0.34	2.21	0.29	2.06	0.7887
Can't Work Group	0.12	1.08	0.06	0.77	0.4169
Returners - Anxiety	0.00	0.00	0.00	0.00	-
Returners - Self Compassion	0.00	0.00	0.00	0.00	-
Returners - Time Management	0.00	0.00	0.00	0.00	-
Workshop - Anxiety	0.04	0.37	0.02	0.31	0.6591
Workshop - CBT for self-help	0.01	0.22	0.01	0.22	0.9963
Workshop - Exam Preparation	0.01	0.22	0.01	0.22	0.9963
Workshop - Food and mood	0.05	0.43	0.00	0.00	0.0453
Workshop - Procrastination	0.05	0.43	0.06	0.48	0.7302
Workshop - Self-compassion	0.05	0.43	0.05	0.53	0.9934
Workshop - Sleep	0.00	0.00	0.00	0.00	-
Workshop - Social anxiety	0.00	0.00	0.00	0.00	-
Workshop - Panic attacks	0.02	0.31	0.01	0.22	0.5669
Assertiveness Group	0.28	2.81	0.19	2.31	0.6591
Bereavement Group	0.14	2.44	0.28	3.46	0.5601
M Phil Group	0.00	0.00	0.00	0.00	-
Managing Mood Group	0.14	2.44	0.00	0.00	0.3181
Perfectionism Group	0.00	0.00	0.12	2.04	0.3181
Post Graduate Group	0.32	5.70	0.33	5.72	0.9963
Returners Group	0.02	0.23	0.01	0.16	0.5669
Self-esteem Group	0.12	2.04	0.12	2.04	0.9963
UG Group	0.23	4.07	0.00	0.00	0.3181

6.9 CORE-6D utilities by arm and cohort

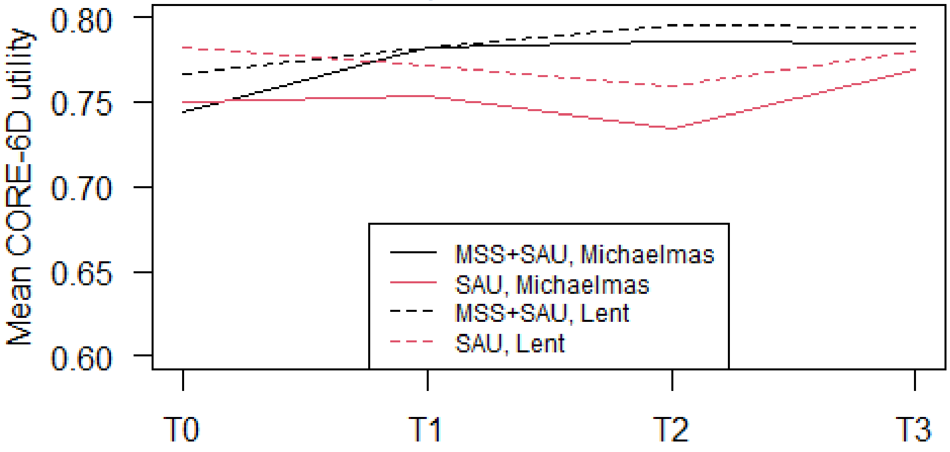

Figure 6.4: Mean (per student) CORE-6D utilities by arm and cohort (N within each group varies by time point – see Table 1).

For peer review only - http://bmjopen.bmj.com/site/about/guidelines.xhtml

6.10 Cost-effectiveness planes

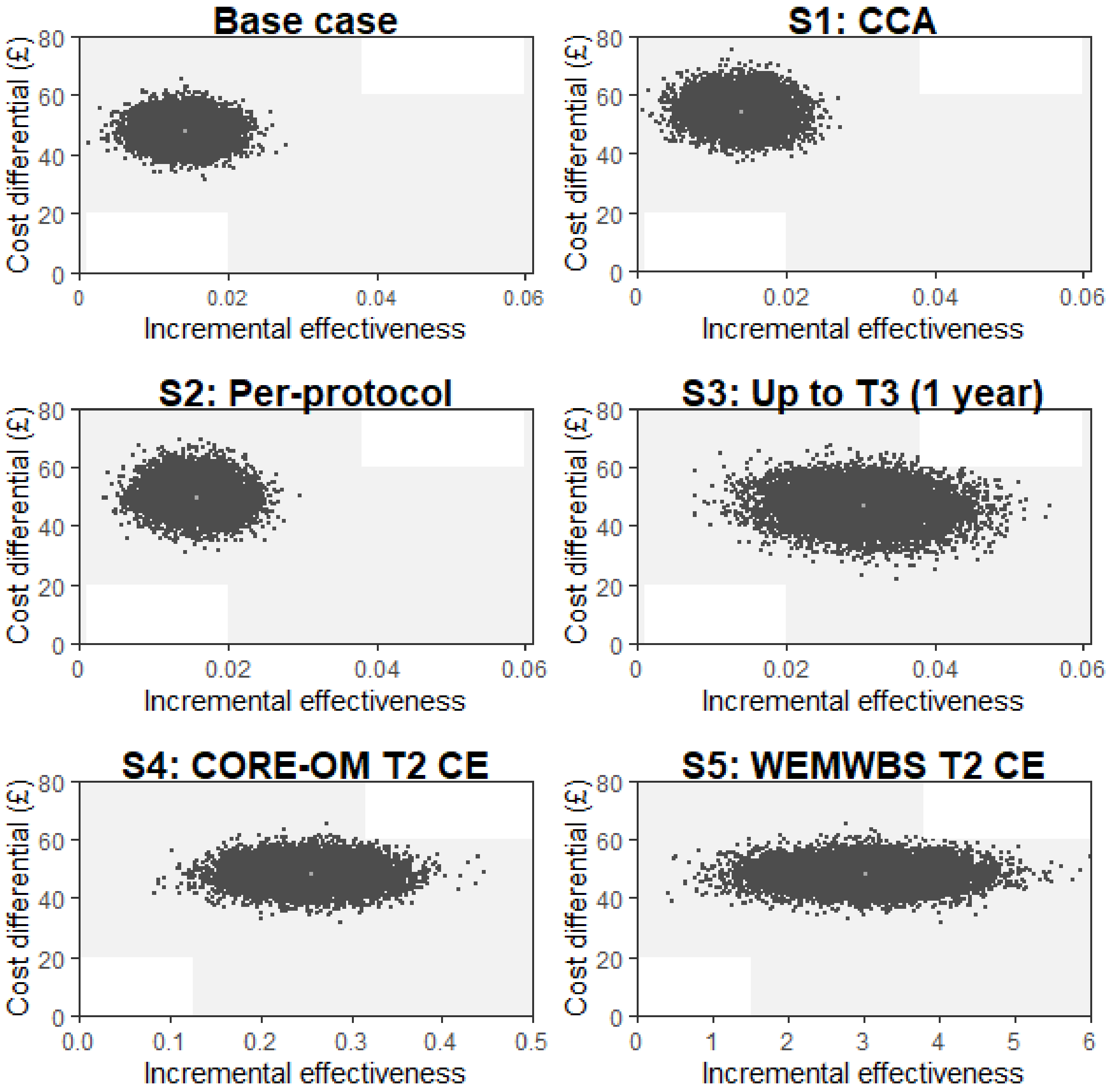

Figure 6.5: Cost-effectiveness planes resulting from the bootstrap re-sampling

For peer review only - http://bmjopen.bmj.com/site/about/guidelines.xhtml

Husereau D, Drummond M, Petrou S, Carswell C, Moher D, Greenberg D, et al. Consolidated Health Economic Evaluation Reporting Standards (CHEERS)--explanation and elaboration: a report of the ISPOR Health Economic Evaluation Publication Guidelines Good Reporting Practices Task Force. Value Health. 2013;16(2):231-50.

CHEERS checklist. Unless indicated otherwise, page numbers relate to the main text file.

**Section/item**	**Item No**	**Recommendation**	**Reported on page/section**
**Title and abstract**			
Title	1	Identify the study as an economic evaluation or use more specific terms such as "cost-effectiveness analysis", and describe the interventions compared.	p.1
Abstract	2	Provide a structured summary of objectives, perspective, setting, methods (including study design and inputs), results (including base case and uncertainty analyses), and conclusions.	p.2
**Introduction**			
Background and objectives	3	Provide an explicit statement of the broader context for the study.	p.3, Section 1
		Present the study question and its relevance for health policy or practice decisions.	p.3, Section 1
**Methods**			
Target population and subgroups	4	Describe characteristics of the base case population and subgroups analysed, including why they were chosen.	p.3, Section 2.1
Setting and location	5	State relevant aspects of the system(s) in which the decision(s) need(s) to be made.	p.3, Section 2.1
Study perspective	6	Describe the perspective of the study and relate this to the costs being evaluated.	p.4, Section 2.2
Comparators	7	Describe the interventions or strategies being compared and state why they were chosen.	p.3, Section 2.1
Time horizon	8	State the time horizon(s) over which costs and consequences are being evaluated and say why appropriate.	p.6-7, Section 2.4
Discount rate	9	Report the choice of discount rate(s) used for costs and outcomes and say why appropriate.	Noted not applicable, p.7, Section 2.4
Choice of health outcomes	10	Describe what outcomes were used as the measure(s) of benefit in the evaluation	p.5-6, Section 2.3

For peer review only - http://bmjopen.bmj.com/site/about/guidelines.xhtml

		and their relevance for the type of analysis performed.	
Measurement of effectiveness	11a	*Single study-based estimates:* Describe fully the design features of the single effectiveness study and why the single study was a sufficient source of clinical effectiveness data.	p.5, Section 2.3
	11b	*Synthesis-based estimates:* Describe fully the methods used for identification of included studies and synthesis of clinical effectiveness data.	Not applicable
Measurement and valuation of preference based outcomes	12	If applicable, describe the population and methods used to elicit preferences for outcomes.	(Not elicited in this study, but relevant details p.5, Section 2.3)
Estimating resources and costs	13a	*Single study-based economic evaluation:* Describe approaches used to estimate resource use associated with the alternative interventions. Describe primary or secondary research methods for valuing each resource item in terms of its unit cost. Describe any adjustments made to approximate to opportunity costs.	p.4, Section 2.2, p.8, Section 3.3, Table 2-4<br><br>Supplementary materials
	13b	*Model-based economic evaluation:* Describe approaches and data sources used to estimate resource use associated with model health states. Describe primary or secondary research methods for valuing each resource item in terms of its unit cost. Describe any adjustments made to approximate to opportunity costs.	Not applicable
Currency, price date, and conversion	14	Report the dates of the estimated resource quantities and unit costs. Describe methods for adjusting estimated unit costs to the year of reported costs if necessary. Describe methods for converting costs into a common currency base and the exchange rate.	As applicable, p.4, Section 2.2
Choice of model	15	Describe and give reasons for the specific type of decision-analytical model used. Providing a figure to show model structure is strongly recommended.	Not applicable
Assumptions	16	Describe all structural or other assumptions underpinning the decision-analytical model.	Not applicable
Analytical methods	17	Describe all analytical methods supporting the evaluation. This could include methods for dealing with skewed, missing, or censored data; extrapolation methods; methods for pooling data;	p.6-7, Section 2.4<br><br>Supplementary materials

For peer review only - http://bmjopen.bmj.com/site/about/guidelines.xhtml

		approaches to validate or make adjustments (such as half cycle corrections) to a model; and methods for handling population heterogeneity and uncertainty.	
**Results**			
Study parameters	18	Report the values, ranges, references, and, if used, probability distributions for all parameters. Report reasons or sources for distributions used to represent uncertainty where appropriate. Providing a table to show the input values is strongly recommended.	Not applicable
Incremental costs and outcomes	19	For each intervention, report mean values for the main categories of estimated costs and outcomes of interest, as well as mean differences between the comparator groups. If applicable, report incremental cost-effectiveness ratios.	p.10-12, Sections 3.2-3.5 Tables 2-5<br><br>Supplementary materials
Characterising uncertainty	20a	*Single study-based economic evaluation:* Describe the effects of sampling uncertainty for the estimated incremental cost and incremental effectiveness parameters, together with the impact of methodological assumptions (such as discount rate, study perspective).	p.12, Section 3.6 Figure 2<br><br>Supplementary materials
	20b	*Model-based economic evaluation:* Describe the effects on the results of uncertainty for all input parameters, and uncertainty related to the structure of the model and assumptions.	Not applicable
Characterising heterogeneity	21	If applicable, report differences in costs, outcomes, or cost-effectiveness that can be explained by variations between subgroups of patients with different baseline characteristics or other observed variability in effects that are not reducible by more information.	Not applicable
**Discussion**			
Study findings, limitations, generalisability, and current knowledge	22	Summarise key study findings and describe how they support the conclusions reached. Discuss limitations and the generalisability of the findings and how the findings fit with current knowledge.	p.14-16 Sections 4-5
**Other**			
Source of funding	23	Describe how the study was funded and the role of the funder in the identification, design, conduct, and reporting of the	p.16

For peer review only - http://bmjopen.bmj.com/site/about/guidelines.xhtml

		analysis. Describe other non-monetary sources of support.	
Conflicts of interest	24	Describe any potential for conflict of interest of study contributors in accordance with journal policy. In the absence of a journal policy, we recommend authors comply with International Committee of Medical Journal Editors recommendations.	p.16

For peer review only

For peer review only - http://bmjopen.bmj.com/site/about/guidelines.xhtml
